# Organic synthesis and anti-influenza A virus activity of cyclobakuchiols A, B, C, and D

**DOI:** 10.1371/journal.pone.0248960

**Published:** 2021-03-26

**Authors:** Masaki Shoji, Tomoyuki Esumi, Narue Tanaka, Misa Takeuchi, Saki Yamaji, Mihiro Watanabe, Etsuhisa Takahashi, Hiroshi Kido, Masayuki Yamamoto, Takashi Kuzuhara

**Affiliations:** 1 Faculty of Pharmaceutical Sciences, Laboratory of Biochemistry, Tokushima Bunri University, Tokushima, Japan; 2 Faculty of Pharmaceutical Sciences, Institute of Pharmacognosy, Tokushima Bunri University, Tokushima, Japan; 3 Division of Pathology and Metabolome Research for Infectious Disease and Host Defense, Institute for Enzyme Research, University of Tokushima, Tokushima, Japan; 4 Department of Integrative Genomics, Tohoku University Tohoku Medical Megabank Organization, Sendai, Japan; 5 Department of Medical Biochemistry, Tohoku University Graduate School of Medicine, Sendai, Japan; Cairo University, EGYPT

## Abstract

Novel antiviral agents for influenza, which poses a substantial threat to humans, are required. Cyclobakuchiols A and B have been isolated from *Psoralea glandulosa*, and cyclobakuchiol C has been isolated from *P*. *corylifolia*. The structural differences between cyclobakuchiol A and C arise due to the oxidation state of isopropyl group, and these compounds can be derived from (+)-(*S*)-bakuchiol, a phenolic isoprenoid compound present in *P*. *corylifolia* seeds. We previously reported that bakuchiol induces enantiospecific anti-influenza A virus activity involving nuclear factor erythroid 2-related factor 2 (Nrf2) activation. However, it remains unclear whether cyclobakuchiols A–C induce anti-influenza A virus activity. In this study, cyclobakuchiols A, B, and C along with cyclobakuchiol D, a new artificial compound derived from cyclobakuchiol B, were synthesized and examined for their anti-influenza A virus activities using Madin-Darby canine kidney cells. As a result, cyclobakuchiols A–D were found to inhibit influenza A viral infection, growth, and the reduction of expression of viral mRNAs and proteins in influenza A virus-infected cells. Additionally, these compounds markedly reduced the mRNA expression of the host cell influenza A virus-induced immune response genes, interferon-β and myxovirus-resistant protein 1. In addition, cyclobakuchiols A–D upregulated the mRNA levels of NAD(P)H quinone oxidoreductase 1, an Nrf2-induced gene, in influenza A virus-infected cells. Notably, cyclobakuchiols A, B, and C, but not D, induced the Nrf2 activation pathway. These findings demonstrate that cyclobakuchiols have anti-influenza viral activity involving host cell oxidative stress response. In addition, our results suggest that the suitably spatial configuration between oxidized isopropyl group and phenol moiety in the structure of cyclobakuchiols is required for their effect.

## Introduction

**Fig 1:**
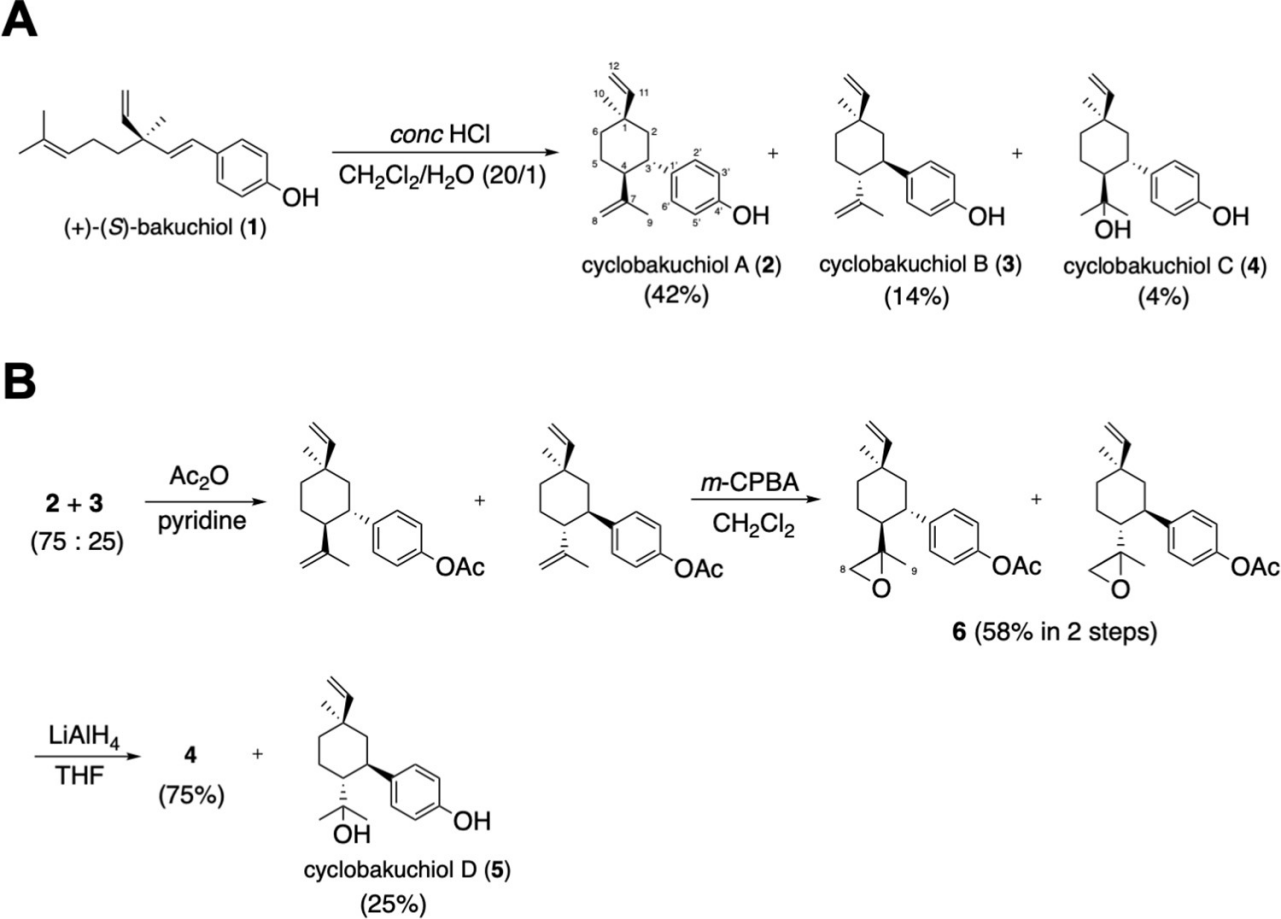
Chemical structures and synthetic schemes of cyclobakuchiols A, B, C, and D. (A) Transformation of (+)-(*S*)-bakuchiol (**1**) to cyclobakuchiols A (**2**), B (**3**), and C (**4**) under acidic condition. (B) Transformation of **2** and **3** to **4** and cyclobakuchiol D (**5**) by acetylation, epoxidation, and reduction.

The World Health Organization (WHO) estimates that approximately 3 to 5 million cases of severe influenza infection occur annually, with approximately 290,000 to 650,000 mortalities [1]. Thus, influenza poses a substantial threat to public health. M2 ion channel inhibitors, adamantane antiviral drugs, such as amantadine and rimantadine, and neuraminidase (NA) inhibitors, such as oseltamivir, zanamivir, and peramivir, have been approved as anti-influenza viral drugs [2]. However, strains of influenza that are resistant to M2-ion channel inhibitors continue to circulate, and oseltamivir-resistant influenza strains have been detected in some cases of H1N1 influenza in 2009 and seasonal H1N1 influenza between 2007 and 2009, whereas a low prevalence of H3N2 and H5N1 viruses has been reported [2–7]. Additionally, baloxavir, which targets the cap-dependent endonuclease activity of polymerase acid (PA) protein, was approved in 2018 [8]. However, baloxavir-resistant influenza A H1N1 and H3N2 viruses in I38T/M/F-substituted PA have been detected, with records of human-to-human transmission of a PA-I38 mutant H3N2 virus [9,10]. Therefore, there is an urgent need to develop novel anti-influenza drugs to prevent and control potential influenza epidemics and pandemics. In particular, anti-influenza viral drugs targeting host factors related to the viral life cycle, but not those of influenza viral proteins, are required.

(+)-(*S*)-bakuchiol (**1**) (Fig 1A) is a phenolic isoprenoid with a chiral tetra-alkylated (all-carbon) quaternary center that has been isolated from the seeds of *Psoralea corylifolia* [11]. Cyclobakuchiols A (**2**) (Fig 1A) and B (**3**) (Fig 1A) were isolated as a mixture of diastereomers from the bioactive guide fraction of the dichloromethane extract of *P*. *glandulosa* and found to possess anti-inflammatory and anti-pyretic activities [12,13]. Cyclobakuchiol C (**4**) (Fig 1A) was isolated from the non-polar fraction of *P*. *corylifolia* seeds [14]. The structural differences between cyclobakuchiols arise from the distinct configurations and the oxidation state of isopropyl group, as shown in Fig 1A. **2**, **3**, and **4** can be synthesized from **1** in a few steps, and their absolute structures determined using NOESY spectra data were found to analogous to the absolute configuration of the chiral tetra-alkylated quaternary center of bakuchiol [15] (Fig 1A). Backhouse et al. reported that a mixture of **2** and **3** possesses a higher degree of anti-inflammatory and antipyretic activities than **1** alone. Bakuchiol has been reported to exert a range of biological and pharmacological effects, including anti-microbial [16], antioxidant [17,18], anti-inflammatory [19,20], and anti-tumor [21,22] activities. Although we recently reported that bakuchiol inhibits enantiospecific influenza A H1N1 viral infection and growth involving nuclear factor erythroid 2-related factor 2 (Nrf2) activation [23], it remains unclear whether **2**, **3**, **4**, and their derivatives possess anti-influenza A virus activity. Therefore, the aim of this study was to determine whether **2**, **3** and **4**, and cyclobakuchiol D (**5**) (Fig 1B), a new artificial compound derived from **2** and **3** (Fig 1B), induce anti-influenza A virus activity and investigate the structure required for anti-viral activity.

## Materials and methods

### Chemicals and instruments

Commercial-grade reagents and solvents were used as obtained, without further purification. *conc*. HCl, CH_2_Cl_2_, hexane, 2-propanol, benzene, *m*-chloroperbenzoic acid (*m*-CPBA), LiAlH_4_, NaHCO_3_, and Na_2_S_2_O_3_ were obtained from Nacalai Tesque (Kyoto, Japan). Anisaldehyde, acetic anhydride, and pyridine were procured from Tokyo Chemical Industry (Tokyo, Japan). EtOH, CDCl_3_, and CD_3_OD were procured from Kanto Chemical (Tokyo, Japan). High-pressure liquid chromatography (HPLC) system consisted of a pump (PU-2089, JASCO, Tokyo, Japan), UV detector (UV-2075, JASCO, Tokyo, Japan), chromato-integrator (D-2500, HITACHI, Tokyo, Japan), column (CHIRALPAK^®^ IA-3, 4.6 × 250 mm, 3 μm, DAICEL, Osaka, Japan), and solvent (hexane/2-propanol = 100/1, flow speed = 3 mL/min). Fourier-transform infrared (FTIR) spectra were measured using a JASCO FT/IR-410 infrared spectrophotometer. Nuclear magnetic resonance (NMR) spectra were recorded on a Varian Unity600 NMR400 spectrometer. Chemical shifts are reported in parts per million (ppm). For the ^1^H NMR spectra, tetramethylsilane was used as an internal reference (0.0 ppm), while for the ^13^C NMR spectra (CDCl_3_), the residual solvent peak was used as the reference (77.1 ppm). For CD_3_OD, the reference used for the ^1^H and ^13^C NMR spectra was the residual solvent peak (3.30 ppm and 49.0 ppm, respectively). The mass spectra were recorded using a JEOL JMS-700 instrument. Analytical thin layer chromatography (TLC) was performed using E. Merck pre-coated TLC plates (silica gel 60F-254, layer thickness 0.25 mm; Merck KGaA, Darmstadt, Germany) and visualized by ultraviolet irradiation or 5% anisaldehyde/5% H_2_SO_4_ in EtOH. Silica gel chromatography was performed using silica gel 60 N (63–210 μm, Kanto Chemical).

### Preparation of cyclobakuchiols A–D

The synthesis of cyclobakuchiols A (**2**), B (**3**), C (**4**), and D (**5**) was initiated from the synthetic (*S*)-(+)-bakuchiol (**1**) [24], which was used as the starting material, as shown in Fig 1. Various conditions for the transformation of **1** into **2** and **3**, including under acidic conditions using protic or Lewis acids, were examined. It was found that the treatment of **1** with an excess amount of *conc*. HCl in CH_2_Cl_2_/H_2_O (20/1) gave rise to a diastereomeric mixture of **2** and **3** (56%, **2**:**3** = 75:25) with a small amount of **4** (3%). Although **2** and **3** were inseparable by flash silica gel column chromatography, separation by HPLC allowed for **2** and **3** single isomers to be obtained. Their structures were confirmed by comparing the spectral data (IR, HRMS, ^1^H NMR and ^13^C NMR) to previous reports [12,15]. Subsequently, the direct transformation from **2** and **3** to a mixture of **4** and **5** under acidic conditions was attempted, but was unsuccessful because of the unstable properties of phenol moiety. Therefore, the phenolic hydroxy groups of **2** and **3** (75:25) were protected using an acetyl group, followed by treatment with *m*-CPBA to produce a diastereomeric mixture of epoxide **6** in moderate yield. Finally, the reduction of the epoxy and acetyl groups of **6** by LiAlH_4_ resulted in a diastereomeric mixture of **4** and **5** in satisfactory yields. Diastereomers **4** and **5** were not separable by flash column chromatography. Thus, separation was performed under conditions similar to those described previously using HPLC, resulting in **4** as the major product and **5** as the minor product. The structure of **4** was confirmed by comparing the spectral data ([α]_D_, IR, EI-MS, and ^1^H and ^13^C NMR) to a previous report [14]. Similarly, the absolute configuration of **5** was determined based on the quantitative relationship between **4** and **5**. The ^1^H and ^13^C NMR spectra of compounds **2**–**5** and **6** are shown in S1 Fig.

For the synthesis of **2** and **3** started, *conc*. HCl (0.4 mL) was added to a solution of **1** (101.5 mg, 0.396 mmol) in CH_2_Cl_2_/H_2_O (20/1, 2 mL) at room temperature. The mixture was stirred overnight, quenched with saturated NaHCO_3_ (2 mL) and extracted with CH_2_Cl_2_ (3 × 10 mL) before drying over anhydrous MgSO_4_, filtering, and concentrating. The crude mixture was purified by silica gel column chromatography (SiO_2_ = 3 g, hexane/ethyl acetate = 10/1) to obtain a diastereomeric mixture of **2**, **3** (57.1 mg, 56%, **2**: **3** = 75: 25), and **4** (3.1 mg, 3%) (Fig 1A). Furthermore, a part of the diastereomeric mixture of **2** and **3** was separated by HPLC to afford **2** (RT: 9.08 min) and **3** (RT: 11.22 min), respectively. **Data for 2:** [α]_D_^21^–8.0˚ (*c* 0.1, CHCl_3_); IR (neat) 3332, 1642, 1605, 1509, 1447, 1369 cm^–1^; HRMS (EI) *m/z calc for* C_18_H_24_O, 256.1827 [M^+^]; found 256.1833; ^1^H NMR (400 MHz, CDCl_3_) δ 0.98 (s, 3H, 10-CH_3_), 1.34–1.45 (m, 2H, 2-CH_2_(a), 5-CH_2_(a)), 1.48 (s, 3H, 9-CH_3_), 1.53–1.70 (m, 2H, 2-CH_2_(b), 5-CH_2_(b)), 1.78–1.84 (m, 2H, 6-CH_2_), 2.21 (dt, *J* = 4.0, 11.6 Hz, 1H, 4-CH), 2.66 (dt, *J* = 3.6, 11.6 Hz, 1H, 3-CH), 4.51, (brs, 1H, 8-CH_2_(a)), 4.53 (brs, 1H, 8-CH_2_(b)), 4.60 (brs, 1H, OH), 5.07 (dd, *J* = 1.2, 18.0 Hz, 1H, 12-CH_2_(a)), 5.13 (dd, *J* = 1.6, 10.8 Hz, 1H, 12-CH_2_(b)), 5.85 (dd, *J* = 11.2, 17.6 Hz, 1H, 11-CH), 6.72 (d, *J* = 8.8 Hz, 2H, 3’,5’-CH), 6.99 (d, *J* = 8.8 Hz, 2H, 2’,6’-CH); ^13^C NMR (100 MHz, CDCl_3_) δ 19.7 (C9), 29.0 (C5), 31.6 (C10), 37.6 (C1), 37.8 (C6), 42.8 (C3), 47.5 (C2), 51.5 (C4), 111.2 (C8), 112.6 (C12), 115.0 (C3’,5’), 128.5 (C2’,6’), 138.3 (C1’), 146.3 (C11), 148.6 (C7), 153.4 (C4’). **Data for 3:** [α]_D_^21^–34.8˚ (*c* 0.05, CHCl_3_); IR (neat) 3338, 2959, 2925, 1644, 1512, 1450, 1372 cm^–1^; HRMS (EI) *m/z calc for* C_18_H_24_O, 256.1827 [M^+^]; found 256.1828; ^1^H NMR (400 MHz, CDCl_3_) δ 1.14 (s, 3H, 10-CH_3_), 1.45–1.77 (m, 6H, 2,5,6-CH_2_), 1.53 (s, 3H, 9-CH_3_), 2.19 (dt, *J* = 4.4, 11.6 Hz, 1H, 4-CH), 2.72 (dt, *J* = 3.6, 12.0 Hz, 1H, 3-CH), 4.54, (brs, 1H, OH), 4.55 (brs, 1H, 12-CH_2_(a)), 4.57 (brs, 1H, 12-CH_2_(b)), 4.86 (dd, *J* = 4.0, 10.8 Hz, 1H, 8-CH_2_(a)), 4.93 (dd, *J* = 4.1, 17.6 Hz, 1H, 8-CH_2_(b)), 5.81 (dd, *J* = 10.8, 17.6 Hz, 1H, 11-CH), 6.72 (d, *J* = 8.4 Hz, 2H, 3’,5’-CH), 7.01 (d, *J* = 8.4 Hz, 2H, 2’,6’-CH); ^13^C NMR (100 MHz, CDCl_3_) δ 19.8 (C9), 22.2 (C10), 28.3 (C5), 36.6 (C6), 37.0 (C1), 42.3 (C3), 46.5 (C2), 51.6 (C4), 109.2 (C12), 111.3 (C8), 115.0 (C3’,5’), 128.7 (C2’,6’), 138.1 (C1’), 148.5 (C7), 150.7 (C11), 153.5 (C4’).

For the synthesis of **6**, acetic anhydride (ca. 50 μL) was added to a solution of diastereomeric mixture of **2** and **3** (**2**: **3** = 75: 25, 10.2 mg, 0.0398 mmol) in pyridine (0.5 mL) at room temperature under argon atmosphere and the mixture was stirred for 1 h. Pyridine was removed through azeotropy by benzene, and the resin was dissolved in CH_2_Cl_2_ (0.2 mL). Then, NaHCO_3_ (34.4 mg) and *m*-CPBA (ca 70% with water; 20.3 mg, 0.0818 mmol) were added to the mixture at 0°C and stirred for 2 h at same temperature. The reaction mixture was quenched with saturated NaHCO_3_−10% Na_2_S_2_O_3_ (1: 1, 10 mL), extracted with ethyl acetate (3 × 10 mL), dried over anhydrous MgSO_4_, filtered, and concentrated. The crude mixture was purified by preparative thin layer chromatography (0.5 × 200 × 200 mm, hexane/ethyl acetate = 4/1) to yield a diastereomeric mixture of epoxides **6** (7.2 mg, 0.0229 mmol, 58% in 2 steps). **Data for 6:** IR (KBr) 2924, 1761, 1503, 1204, 1009, 910 cm^-1^; HRMS (EI) *m/z calc for* C_20_H_26_O_3_, 314.1882 [M^+^]; found 314.1883; ^1^H NMR (400 MHz, CDCl_3_) δ 0.98 (s, 3H, 10-CH_3_), 1.13 (s, 3H, 9-CH_3_), 1.17–1.91 (m, 7H, 2,5,6-CH_2_, 4-CH), 2.04 (d, *J* = 4.8 Hz, 1H, 8-CH_2_ (a)), 2.14 (d, *J* = 4.4 Hz, 1H, 8-CH_2_ (b)), 2.29 (s, 3H, CH_3_(CH_3_CO)), 2.62 (m, 1H, 3-CH), 4.87 (dd, *J* = 1.2, 10.8 Hz, 0.23H, 12-CH_2_ (a1)), 4.93 (dd, *J* = 1.2, 17.6 Hz, 0.23H, 12-CH_2_ (b1)), 5.06 (dd, *J* = 1.2, 17.6 Hz, 0.77H, 12-CH_2_ (a2)), 5.14 (dd, *J* = 1.2, 10.8 Hz, 0.77H, 12-CH_2_ (a2)), 5.80 (dd, *J* = 10.8, 17.6 H, 0.23H, 11-CH(1)), 5.81 (dd, *J* = 10.8, 17.6 H, 0.77, 11-CH(2), 7.00 (d, *J* = 8.8 Hz, 2H, 3’,5’-CH), 7.15 (d, *J* = 8.8 Hz, 2H, 2’,6’-CH); ^13^C NMR (100 MHz, CDCl_3_) δ 16.6 (C9a), 16.7 (C9b), 21.3 (CH(a)_3_CO), 22.1(CH(b)_3_CO), 23.5 (C10b), 24.3 (C10a), 31.6 (C6a), 35.8 (C6b), 36.7 (C5b), 36.8 (C5a), 37.6 (C2a), 37.7 (C2b), 42.2 (C4b), 42.7 (C4a), 45.3 (C3b), 46.1 (C3a), 50.61 (C8a), 50.65 (C8b), 56.8 (C7a), 58.3 (C7b), 109.6 (C12b), 112.9 (C12a), 121.5 (C3’,5’), 128.4 (C2’a,6’a), 128.5 (C2’b,6’b), 142.1 (C1’b), 142.3 (C1’a), 145.8 (C11b), 146.0 (C11a), 149.0 (C4’a), 149.1 (C4’b), 150.2 (C4’c), 169.6 (CH_3_CO).

The synthesis procedure of **4** and **5** started with the addition of LiAlH_4_ (71.8 mg, 1.89 mmol) to a solution of epoxide **6** (19.5 mg, 0.0620 mmol) in anhydrous THF (0.5 mL) under argon atmosphere at 0°C. Immediately, the mixture was allowed to warm up to room temperature, stirred for 10 min at the same temperature, and refluxed for 30 min. The reaction mixture was quenched with saturated NaHCO_3_−14% citric acid (1:1, 10 mL) at 0°C, then extracted with ethyl acetate (3 × 10 mL), dried over anhydrous MgSO_4_, filtered, and concentrated. The crude mixture was purified by preparative thin layer chromatography (0.5 × 200 × 200 mm, hexane/ethyl acetate = 1/1) to yield a diastereomeric mixture of **4** and **5** (17.0 mg, 0.0620 mmol, 100%) (Fig 1B). Furthermore, a part of the diastereomer was separated by HPLC to afford **4** (RT: 13.78 min) and **5** (RT: 16.95 min). **Data for 4:** [α]_D_^21^–18.6˚ (*c* 0.54, CH_3_OH); IR (neat) 3522, 3270, 2867, 2953, 1605, 1513, 1450, 1375 cm^–1^; HRMS (EI) *m/z calc for* C_18_H_26_O_2_, 274.1933 [M^+^]; found 274.1937; ^1^H NMR (400 MHz, CD_3_OD) δ 0.72 (s, 3H, 10-CH_3_), 0.94 (s, 3H, 8-CH_3_), 0.99 (s, 3H, 9-CH_3_), 1.28–1.52 (m, 3H, 2-CH_2_(a), 5-CH_2_(a), 6-CH_2_(a)), 1.67 (m, 2H, 2-CH_2_(b), 4-CH), 1.81 (m, 1H, 6-CH_2_(b)), 1.91 (m, 1H, 5-CH_2_(b)), 2.56 (dt, *J* = 3.6, 12.4 Hz, 1H, 3-CH), 5.04 (dd, *J* = 1.2, 17.6Hz, 1H, 12-CH_2_(a)), 5.10 (dd, *J* = 1.2, 10.8H, 1H, 12-CH_2_(b)), 5.82 (dd, *J* = 10.8, 18.0 Hz, 1H, 11-CH), 6.69 (d, *J* = 8.0 Hz, 2H, 3’,5’-CH), 7.00 (d, *J* = 8.0 Hz, 2H, 4’,6’-CH); ^13^C NMR (100 MHz, CD_3_OD) δ 25.2 (C5), 26.1(C9), 30.1 (C8), 31.9 (C10), 38.5 (C1), 38.8 (C6), 43.4 (C3), 49.9 (C2), 54.0 (C4), 74.8 (C7), 113.1 (C12), 116.3 (C3’,5’), 129.8 (C2’,6’), 139.3 (C1’), 147.4 (C11), 156.7 (C4’). **Data for 5:** [α]_D_^21^–5.6˚ (*c* 0.215, CH_3_OH); IR (neat) 3528, 3275, 2965, 2830, 1608, 1512, 1455, 1377 cm^–1^; HRMS (EI) *m/z calc for* C_18_H_26_O_2_, 274.1933 [M^+^]; found 274.1931; ^1^H NMR (400 MHz, CDCl_3_) δ 1.08 (s, 3H, 10-CH_3_), 1.17 (s, 3H, 8-CH_3_), 1.19 (s, 3H, 9-CH_3_), 1.22–1.90 (m, 7H, 2, 5, 6-CH_2_, 4-CH), 2.73 (dt, *J* = 4.1, 10.5 Hz, 1H, 3-CH), 4.86 (dd, *J* = 1.2, 9.6 Hz, 1H, 12-CH_2_(a)), 4.92 (dd, *J* = 1.2, 16.2 Hz, 1H, 12-CH_2_(b)), 5.79 (dd, *J* = 9.6, 16.2 Hz, 1H, 11-CH), 6.59 (d, *J* = 8.1 Hz, 2H, 3’,5’-CH), 7.10 (d, *J* = 8.1 Hz, 2’,6’-CH); ^13^C NMR (100 MHz, CDCl_3_) δ 21.8 (C5), 24.7 (C9), 25.0 (C8), 29.3 (C10), 36.5 (C1), 36.6 (C6), 40.9 (C3), 47.5 (C2), 53.1 (C4), 75.9 (C7), 109.5 (C12), 116.2 (C3’,5’), 129.2 (C2’,6’), 137.0 (C1’), 150.3 (C11), 155.1 (C4’).

Stock solutions (10 mM) were formed by dissolving **1**–**5** in dimethyl sulfoxide (DMSO) (Sigma-Aldrich, MO, USA).

### Cell culture

Madin-Darby canine kidney (MDCK) cells (Cell Bank, Ibaraki, Japan) were cultured in high-glucose Dulbecco’s modified Eagle’s medium (DMEM) (Wako, Osaka, Japan) supplemented with 10% fetal bovine serum (FBS) (Thermo Fisher Scientific, MA, USA), 100 units/mL penicillin, 100 μg/mL streptomycin (P/S; Thermo Fisher Scientific), and 4 mM l-glutamine at 37°C in the presence of 5% CO_2_.

### Viral strains

The Puerto Rico 8/34 (A/PR/8/34), California 7/09 [A/CA/7/09, 2009 pandemic strain (H1N1pdm09)], or Wisconsin 33 (A/WSN/33) strains of the influenza A H1N1 virus provided by Takahashi E. and Kido H. [25] were used for the experiments. Viral titers were determined via immunostaining of the influenza A viral nucleoprotein (NP), as previously described [23,26,27].

### Thiazolyl blue tetrazolium bromide (MTT) assay

MDCK cells were seeded on a 96-well plate at 1 × 10^4^ cells/well. **1**–**5** (0.8–100 μM) were prepared in DMSO (100 μM, 1%; 50 μM, 0.5%; 25 μM, 0.25%; 12.5 μM, 0.125%; 6.3 μM, 0.063%; 3.1 μM, 0.031%; 1.6 μM, 0.016%; 0.8 μM, 0.008%) and mixed with infection medium (DMEM supplemented with 1% bovine serum albumin (BSA; Wako), P/S and 4 mM l-glutamine). The resulting mixture was added to the cells and incubated for 24 or 72 h at 37°C in the presence of 5% CO_2_. After incubation, cell viability was determined using an MTT cell count kit (Nacalai Tesque) according to the manufacturer’s instructions, as previously described [23,27].

### Analysis of cell viability of influenza A virus-infected MDCK cells by naphthol blue-black staining

MDCK cells were seeded on a 96-well plate (1 × 10^4^ cells/well). **2**–**5** (0.4–25 μM) were mixed with A/PR/8/34, A/CA/7/09, or A/WSN/33 viruses in 10% FBS-supplemented growth medium at a multiplicity of infection (MOI) of ten. DMSO (0.004–1%) or **1** (0.4–25 μM) [23] were used as negative or positive controls, respectively. The resulting mixture was added to the cells after incubation for 30 min at 37°C in the presence of 5% CO_2_ and then incubated for 72 h at 37°C in the presence of 5% CO_2_. After incubation, the cells were stained with naphthol blue–black, as previously described [23,27,28]. Viable cells in each well were stained blue, whereas dead cells remained unstained.

### Immunofluorescence staining of influenza A virus-infected MDCK cells

MDCK cells were seeded on a 96-well plate at 1 × 10^4^ cells/well. **25** (3.1‒12.5 μM) were mixed with A/PR/8/34 or A/WSN/33 viruses at an MOI of 0.1 in the infection medium and incubated for 30 min at 37°C in the presence of 5% CO_2_. Each mixture was added to the cells at 37°C in the presence of 5% CO_2_. DMSO (0.031–0.125%) or **1** (3.1–12.5 μM) were used as negative or positive controls, respectively. After influenza A viral infection for 24 h, the cells were fixed with 4% paraformaldehyde in phosphate-buffered saline (PBS) for 30 min at 4°C and subsequently permeabilized by the addition of 0.3% Triton X-100 for 20 min at 25°C. A mouse primary antibody was used to detect the NP of A/PR/8/34 or A/WSN/33 viruses (FluA-NP 4F1; SouthernBiotech, AL, USA). Alexa Fluor488-conjugated goat anti-mouse IgG (H + L) antibody (Thermo Fisher Scientific) was used as the secondary antibody. Cell nuclei were then stained using diamidino-2-phenylindole (DAPI) (Thermo Fisher Scientific). Wells were photographed using a fluorescence microscope (BIOREVO BZ-X700; Keyence, Osaka, Japan). The proportion of influenza A NP-positive cells per DAPI-positive cells was calculated based on measurements recorded using BZ-X Analyzer software (Keyence).

### Influenza A viral growth assay

MDCK cells were seeded on a 24-well plate at a density of 1 × 10^5^ cells/well. Cells were infected with A/PR/8/34 or A/WSN/33 viruses at an MOI of 0.001 in infection medium for 1 h at 37°C in the presence of 5% CO_2_. Infected cells were washed prior to the addition of **2**–**5** (12.5 μM) in infection medium supplemented with 3 μg/mL l-tosylamido-2-phenyl ethyl chloromethyl ketone (TPCK)-treated trypsin (Sigma-Aldrich). DMSO (0.125%) or **1** (12.5 μM) were used as negative or positive controls, respectively. The cells were then incubated for 12, 24, 48, or 72 h at 37°C in the presence of 5% CO_2_. Culture media were collected from each well at the indicated time points. Viral titers (plaque forming units per mL [PFU/mL]) were calculated as previously described [23,27].

### Reverse transcription and quantitative polymerase chain reaction (RT-qPCR)

MDCK cells were seeded on a 24-well plate at a density of 1 × 10^5^ cells/well. **2**–**5** (12.5 μM), **1** (12.5 μM, positive control) or DMSO (0.125%, negative control) were mixed with or without MOI 0.1 of A/PR/8/34 virus and incubated for 30 min prior to the cells being added. Uninfected cells (UI) were used for mock infection. After incubation for 24 h, total RNA was extracted from cell lysates using a RNeasy Mini Kit (Qiagen GmbH, Germany). Total RNA was used to synthesize cDNA using SuperScript VILO (Thermo Fisher Scientific) according to the manufacturer’s instructions. The synthesized cDNA was used as a template for qPCR, which was performed using SYBR Green real-time PCR Master Mix (TOYOBO, Osaka, Japan). The primers used are shown in S1 Table. PCR and data analyses were performed on an Applied Biosystems StepOne Plus Real-time PCR system (Thermo Fisher Scientific). Relative expression was calculated using the ΔΔCT method. The levels of viral mRNAs encoding *NP*, nonstructural protein 1 (*NS1*), polymerase subunits (*PA*, *PB1*, and *PB2*), and matrix 2 (*M2*) genes were normalized to that of 18s ribosomal RNA (rRNA) [23], and the mRNA levels of canine interferon-β (*Ifn-β*), myxovirus-resistant protein 1 (*Mx1*) or NAD(P)H quinone oxidoreductase 1 (*Nqo1*) and *Firefly* or *Renilla* luciferase genes were normalized to that of β-actin.

### Western blotting

MDCK cells were seeded on a 24-well plate at a density of 1 × 10^5^ cells/well. **2**–**5** (12.5 μM), **1** (12.5 μM, positive control), or DMSO (0.125%, negative control) were mixed with MOI 0.1 of A/PR/8/34 virus and incubated for 30 min prior to the cells being added. At 4, 8, 12, or 24 h post-infection, the cells were lysed in a buffer containing 125 mM Tris-HCl, pH 6.8, 5% sodium dodecyl sulfate, 25% glycerol, 0.1% bromophenol blue, and 10% β-mercaptoethanol and boiled for 5 min. The cell lysates were separated on a 10% polyacrylamide gel. The proteins were transferred to a polyvinylidene fluoride microporous membrane (Millipore, MA, USA). FluA-NP 4F1 (SouthernBiotech) or a goat anti-influenza A viral NS1 antibody (vC-20; Santa Cruz Biotechnology, CA, USA) were used as primary antibodies to detect their respective proteins. A rabbit anti-β-ACTIN antibody (13E5; Cell Signaling, MA, USA) was used as an internal control. The secondary antibodies, horseradish peroxidase (HRP)-conjugated goat anti-mouse IgG (SouthernBiotech) or donkey anti-goat IgG (sc-2020; Santa Cruz Biotechnology), were used as appropriate. The signals were detected using Immobilon Western Chemiluminescent HRP Substrate (Millipore). Signal intensities were measured using ImageJ software, and the protein levels of NP and NS1 were normalized to that of β-actin.

### Nrf2 reporter assay

An Nrf2 reporter assay based on the dual luciferase system was performed as previously described [23,29]. The plasmid pNQO1-ARE (antioxidant response element)-Fluc, provided by Yamamoto M., expressed a *Firefly* luciferase gene driven by Nrf2 activation, while the plasmid pRL-TK-Rluc vector (Promega, CA, USA) expressed a *Renilla* luciferase gene driven by the herpes simplex viral thymidine kinase promoter as an internal control. MDCK cells were seeded in a 24-well plate at 1 × 10^5^ cells/well and transfected with pNQO1-ARE-luc (0.25 μg) and pRL-TK-Rluc (0.25 μg). At 24 h post-transfection, the cells were treated with 12.5 μM cyclobakuchiols A–D in the infection medium at 37°C under 5% CO_2_. DMSO (0.125%) and 12.5 μM **1** were used as negative and positive controls, respectively. Total RNA was extracted from the MDCK cell lysates after 24 h of incubation. The levels of *Firefly* and *Renilla* luciferase mRNAs were analyzed by RT-qPCR and normalized to that of *β-actin* mRNA.

### Statistical analysis

All results are expressed as the mean ± standard error of the mean (SEM) and are representative of three independent experiments. Differences between more than two groups were analyzed by one-way analysis of variance (ANOVA). The results were considered significantly different when *p* < 0.05.

## Results

### Cyclobakuchiols A–D increased the survival of infected MDCK cells and suppressed infection by influenza A virus

**Fig 2:**
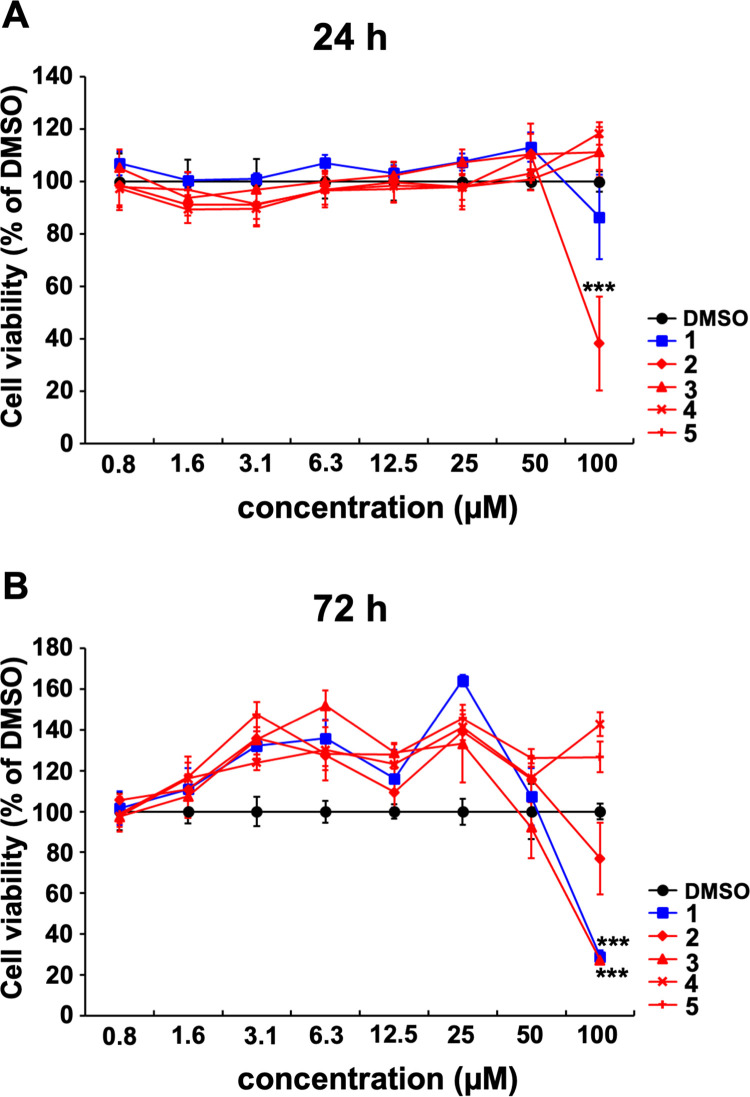
Toxicity of cyclobakuchiols A–D against MDCK cells. The indicated concentrations of cyclobakuchiols A (**2**), B (**3**), C (**4**), and D (**5**) or (+)-(*S*)-bakuchiol (**1**) in DMSO (concentrations of 100 μM, 1%; 50 μM, 0.5%; 25 μM, 0.25%; 12.5 μM, 0.125%; 6.3 μM, 0.063%; 3.1 μM, 0.031%; 1.6 μM, 0.016%; 0.8 μM, 0.008%) were added to the MDCK cells. Cell viabilities were determined by MTT assay after incubation for 24 h (n = 5 each) (A) or 72 h (n = 5 each) (B). Data represent the mean ± SEM and were representative of three independent experiments. ***p < 0.001, for the comparison with DMSO-treatment. The results were reproducible in this experiment.

The cytotoxicity of **2**–**5** against MDCK cells was confirmed using the MTT assay (Fig 2). The viability of cells treated with 100 μM **2** or **1** was reduced after 24 h of incubation (Fig 2A), while the viability of cells treated with 100 μM **3** or **1** was reduced after 72 h of incubation (Fig 2B) compared with that of cells treated with DMSO only. Cells exposed to ≤50 μM **2**–**5** and **1** were not affected at 24 or 72 h (Fig 2A and 2B), respectively. These results indicated that exposure to ≤ 50 μM cyclobakuchiols A–D for 24 or 72 h did not induce toxicity in MDCK cells.

**Fig 3:**
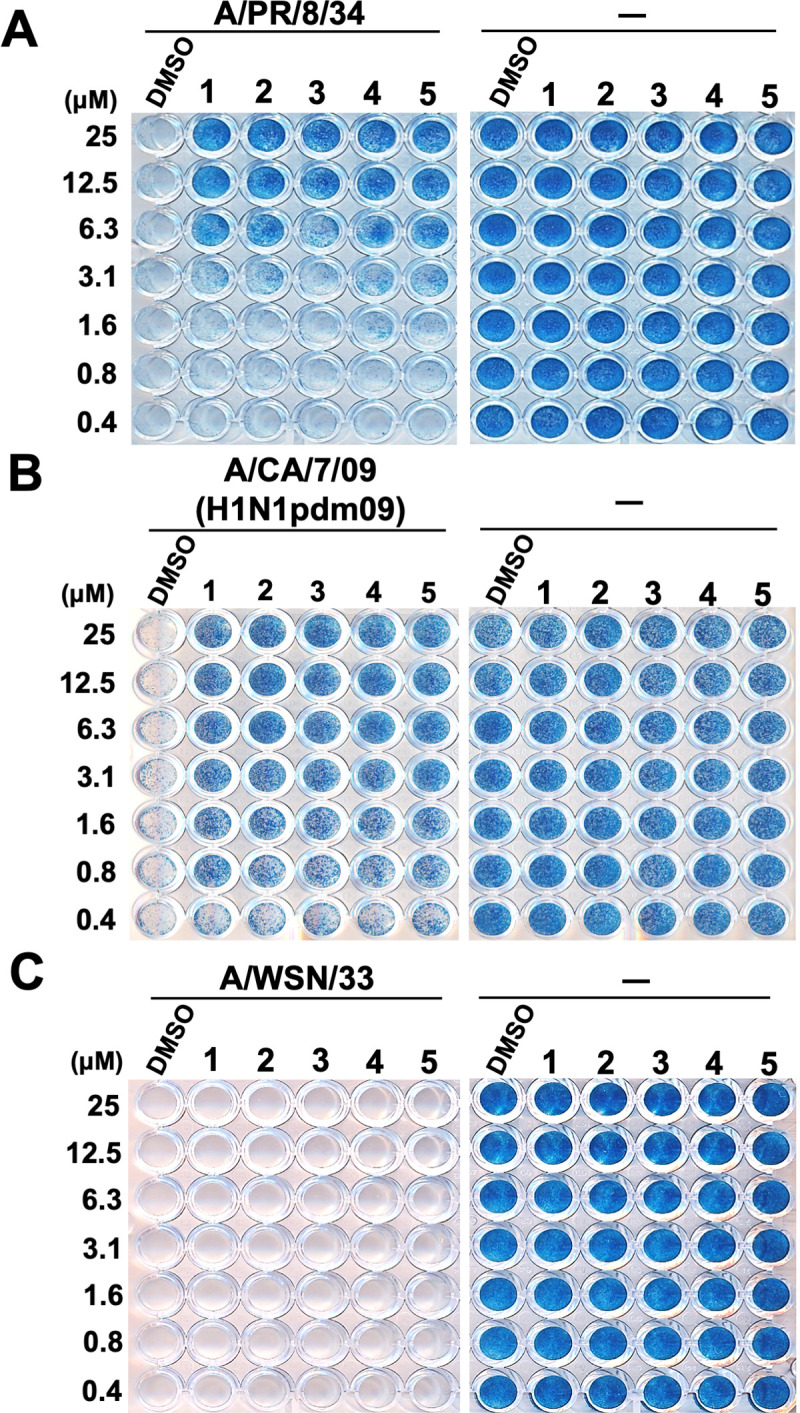
Cyclobakuchiols A–D promotes the viability of MDCK cells infected with influenza A viruses. Effects of cyclobakuchiols A (**2**), B (**3**), C (**4**), and D (**5**) on the viability of MDCK cells infected with influenza A viruses. **2**–**5** (0.4–25 μM) were mixed with or without the A/PR/8/34 (A), A/CA/7/09 (B), or A/WSN/33 (C) viruses and subsequently added to MDCK cells. DMSO (0.004–0.25%) and (+)-(*S*)-bakuchiol (**1**) (0.4–25 μM) were used as negative and positive controls, respectively. Cell viability was determined via naphthol blue-black staining after incubation for three days. Data are representative of three independent experiments. The results were reproducible across all experiments.

Next, to evaluate the anti-influenza virus activities of **2**–**5**, their effect on the survival of influenza A virus-infected MDCK cells were examined. Various concentrations of **2**–**5** were mixed with A/PR/8/34 (Fig 3A), A/CA/7/09 (Fig 3B), or A/WSN/33 (Fig 3C) strains of the influenza A H1N1 viruses (MOI 10) and added to MDCK cells. The cells were then stained after incubation for 72 h. Samples without the addition of viruses were stained blue at all indicated concentrations (Fig 3A–3C, right panels), suggesting that the concentrations used in this experiment did not induce toxicity in MDCK cells. Cells exposed to DMSO and infected with A/PR/8/34, A/CA/7/09, or A/WSN/33 (Fig 3A–3C, left panels) viruses were not stained. However, cells treated with 6.3–25 μM **2**–**5** and infected with A/PR/8/34 virus (Fig 3A, left panel) or 0.8–25 μM **2**–**5** and infected with A/CA/7/09 virus (Fig 3B, left panel) were stained blue, indicating that the cells remained viable following exposure to A/PR/8/34 and A/CA/7/09 viruses. A/WSN/33 virus-infected cells treated with **2**–**5** or **1** were not stained (Fig 3C, left panel). Therefore, these results showed that cyclobakuchiols A–D increased the viability of MDCK cells infected with the influenza A virus H1N1 strain, but the effects varied depending on the viral subtype.

### Cyclobakuchiols A–D inhibited influenza A viral infection and growth

**Fig 4:**
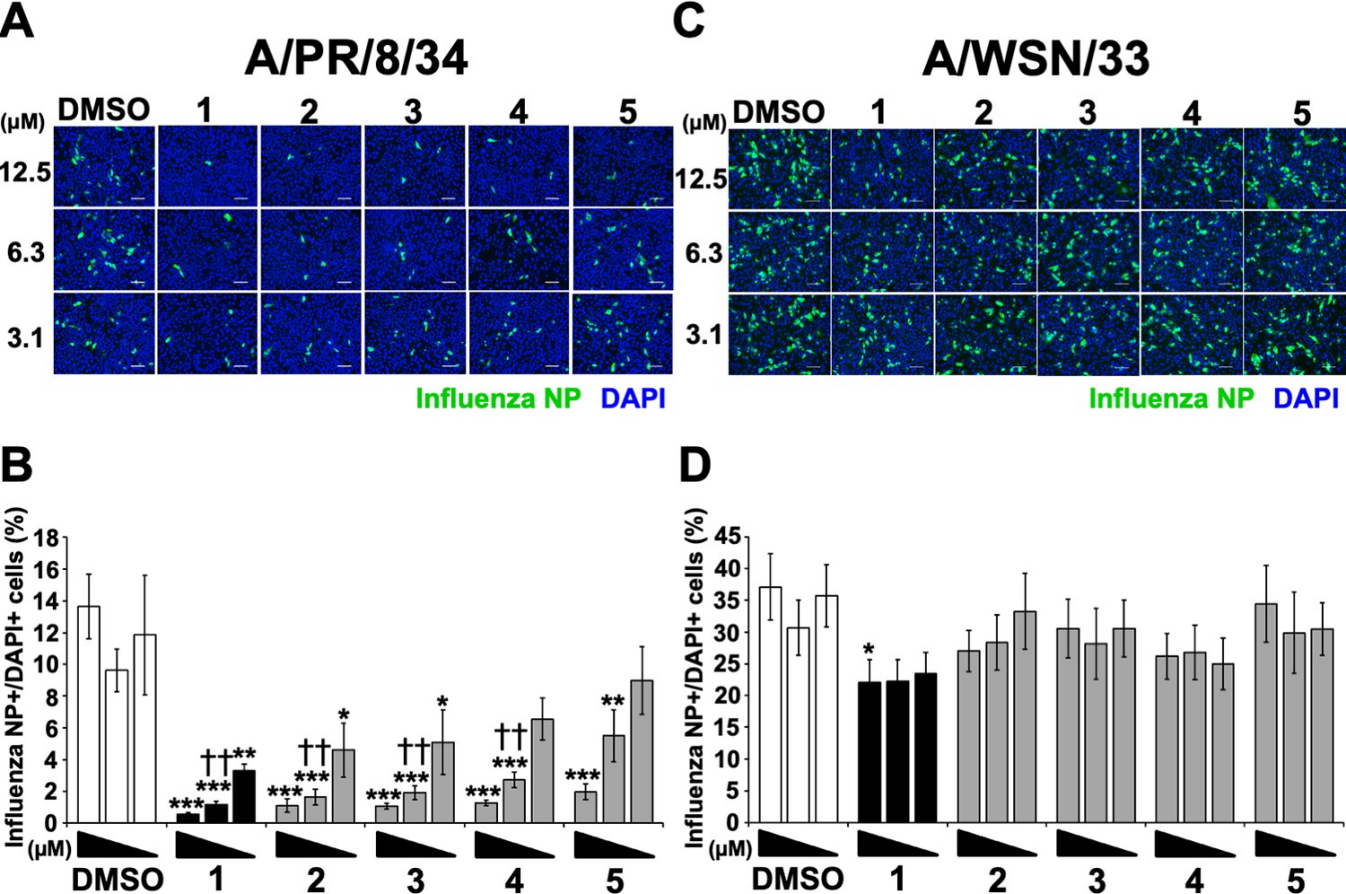
Image analysis of inhibitory effect of cyclobakuchiols A–D on influenza A viral infection. The corresponding concentrations of cyclobakuchiols A (**2**), B (**3**), C (**4**), and D (**5**) (3.1–12.5 μM; n = 9 each), (+)-(*S*)-bakuchiol (**1**) (3.1–12.5 μM; n = 9 each), or DMSO (0.031–0.125%; n = 9 each) were mixed with A/PR/8/34 (A and B) or A/WSN/33 (C and D) viruses and added to MDCK cells for 24 h. The infected MDCK cells were visualized by immunofluorescence staining of influenza A viral NP and then photographed under a microscope (A and C). The percentages of influenza A viral NP-positive cells per DAPI-positive cells were calculated based on the counts of influenza A viral NP-positive and DAPI-positive cells (B and D). The white scale bar in each image represents 100 μm. Data are expressed as the mean ± SEM of three independent experiments. *p < 0.05, **p < 0.01, ***p < 0.001 relative to DMSO-treatment. ^††^*p* < 0.01 relative to cyclobakuchiol D-treatment. The results were reproducible in this experiment.

To investigate whether **2**–**5** inhibited influenza A viral infection, immunofluorescence staining was examined for influenza A viral nucleoprotein (NP) in MDCK cells treated with **2**–**5** or **1** and infected with A/PR/8/34 (Fig 4A and 4B) or A/WSN/33 (Fig 4C and 4D) viruses (MOI 0.1) for a 24-h incubation period. The wells were observed under a microscope and photographed (Fig 4A and 4C). Then, the NP-immunofluorescent stained cells were counted and the percentage of NP-positive cells relative to DAPI-positive cells was calculated (Fig 4B and 4D). The number of NP-stained cells in wells treated with 3.1–12.5 μM **2**–**4**, 6.3–12.5 μM **5**, or 3.1–12.5 μM **1** was reduced compared to the DMSO-treated cells (Fig 4A). Treatment with 6.3–12.5 μM **2**–**4** or **1** and 3.1 μM **2** and **3** or **1** significantly reduced the percentage of influenza A NP-positive cells relative to the DMSO-treated cells (Fig 4B). In addition, treatment with 6.3 μM **2**–**4** or **1** significantly reduced the percentage of influenza A NP-positive cells compared with the **5**-treatment (Fig 4B). In cells infected with A/WSN/33 virus, the number of stained cells in **2**–**5**- or **1**-treated cells was equal to that in the DMSO-treated cells (Fig 4C). Treatment with 3.1–12.5 μM **2**–**5** or 3.1–6.3 μM **1** did not significantly reduce the percentage of influenza A NP-positive cells, but treatment with 12.5 μM **1** significantly reduced this percentage, relative to the treated DMSO cells (Fig 4D). In addition, the half-maximal inhibitory concentration (IC_50_) values of the antiviral effects of cyclobakuchiols A–D against A/PR/8/34 virus based on the number of influenza A virus-infected cells, as shown in the immunofluorescence staining of Fig 4B, were calculated (Table 1). As a result, IC_50_ values of 2.1 ± 1.2, 1.7 ± 0.8, 2.4 ± 0.6, 4.6 ± 2.2, and 8.8 ± 2.4 μM were obtained for bakuchiol and cyclobakuchiols A, B, C, and D, respectively (Table 1). These results indicate that cyclobakuchiols A–D induced an inhibitory effect on influenza A viral infection, which depended on the cyclobakuchiol structure and viral subtypes.

**Table 1:** Anti-viral effects of cyclobakuchiols A–D against A/PR/8/34 virus.

	A/PR/8/34
(+)-(*S*)-bakuchiol (μM)	2.1 ± 1.2
cyclobakuchiol A (μM)	1.7 ± 0.8
cyclobakuchiol B (μM)	2.4 ± 0.6
cyclobakuchiol C (μM)	4.6 ± 2.2
cyclobakuchiol D (μM)	8.8 ± 2.4

The data presented are half maximal (50%) inhibitory concentration (IC_50_) values. Data represent the mean ± SEM and are representative of three independent experiments.

**Fig 5:**
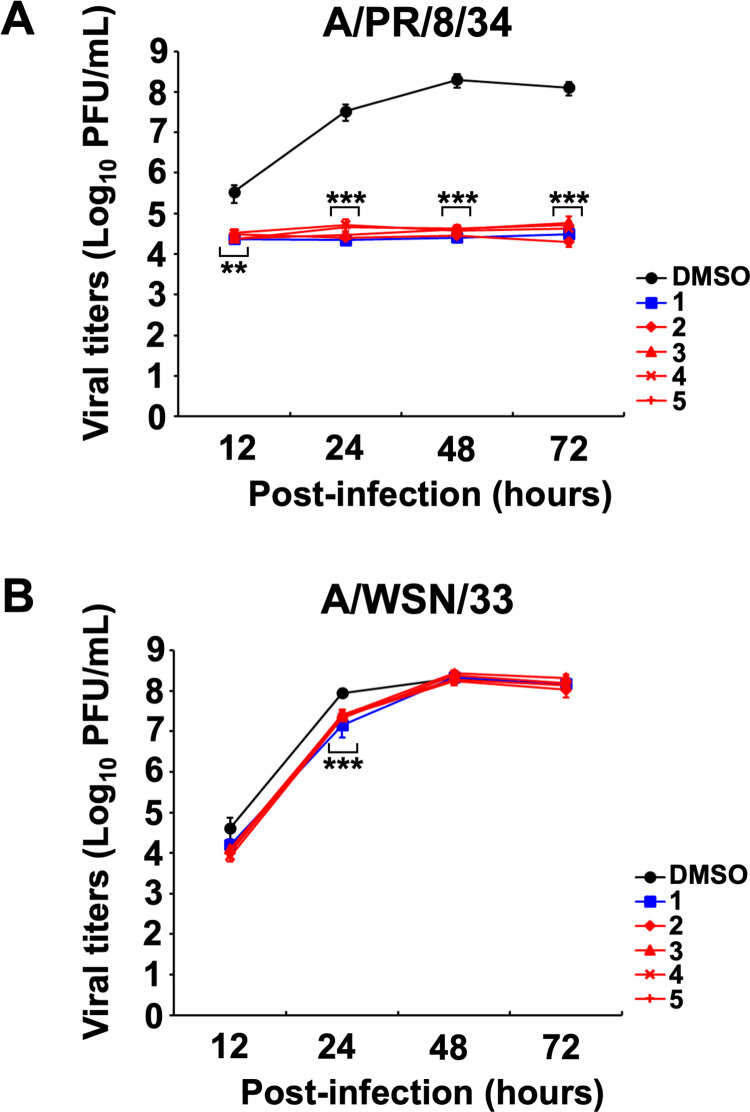
Cyclobakuchiols A–D inhibits influenza A viral growth. MDCK cells were infected with A/PR/8/34 (A) or A/WSN/33 (B) viruses prior to the addition of cyclobakuchiol A (**2**), B (**3**), C (**4**), and D (**5**) (12.5 μM; n = 9), DMSO (0.125%; n = 9) or (+)-(*S*)-bakuchiol (**1**) (12.5 μM; n = 9). The conditioned culture media were collected at the indicated time-points and added to MDCK cells, and the treated cells were immunostained. The viral titers were calculated from the number of stained cells. Data represent the mean ± SEM and are representative of three independent experiments. **p < 0.01, ***p < 0.001 for the comparison of DMSO-treatment. Results were reproducible in this experiment.

In addition, the inhibitory effect on influenza A viral growth following the treatment with A/PR/8/34 (Fig 5A) or A/WSN/33 (Fig 5B) virus-infected MDCK cells with **2**–**5** for 12 to 72 h was investigated. The viral titer of conditioned media from cells infected with A/PR/8/34 virus treated with **2**–**5** or **1** was significantly reduced at 12–72 h (Fig 5A), whereas the viral titer of cells infected with A/WSN/34 virus treated with **2**–**5** or **1** was significantly reduced at 24 h, but was not reduced at 48–72 h, compared with the conditioned media of DMSO-treated cells (Fig 5B).

Taken together, these findings demonstrate that cyclobakuchiols A–D induce an inhibitory effect on influenza A viral infection and growth, however, the effect varies depending on the viral subtype.

### Cyclobakuchiols A–D reduced the expression of influenza A viral mRNAs and proteins

**Fig 6:**
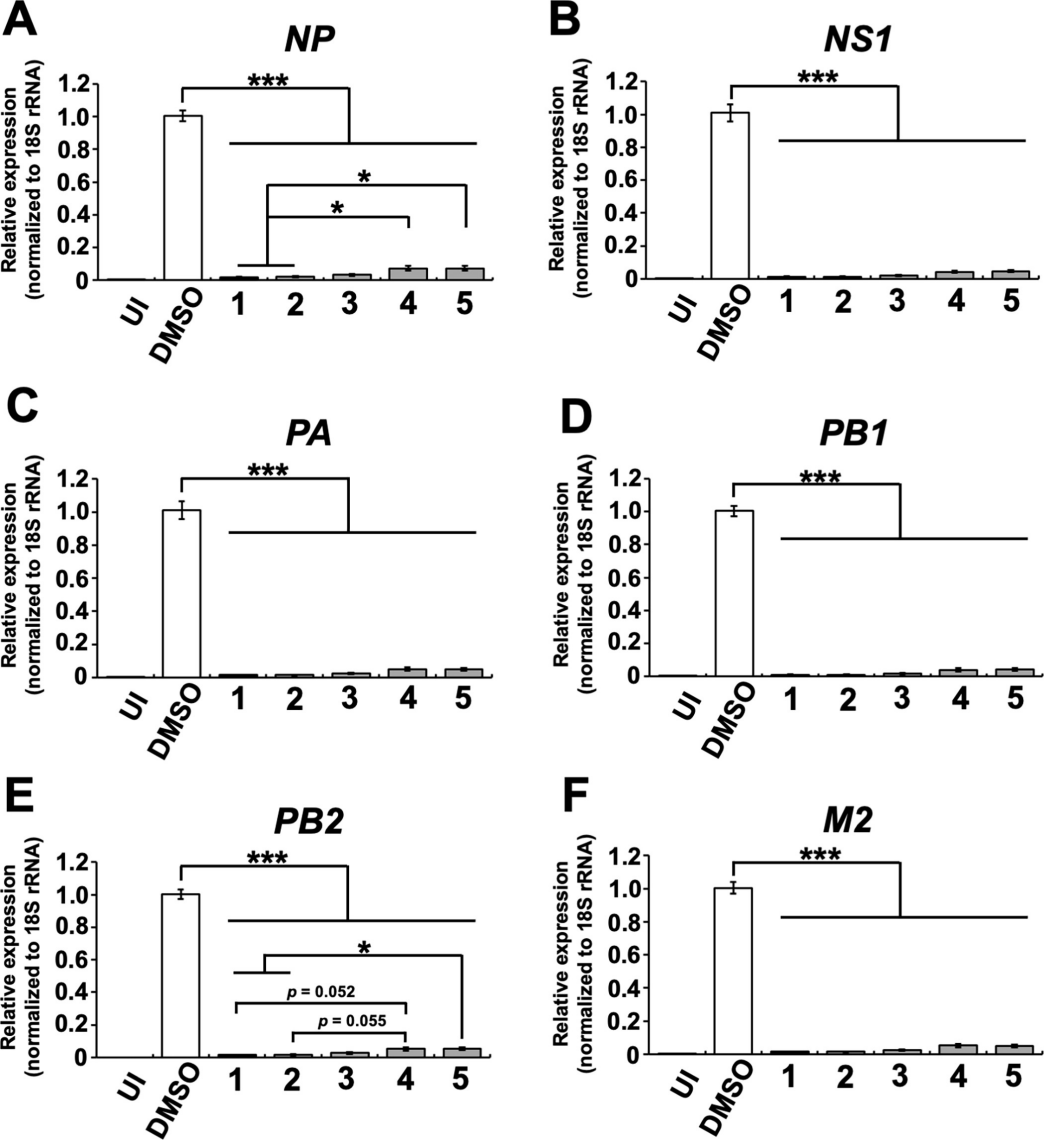
Cyclobakuchiols A–D inhibits the expression of influenza A viral mRNAs. Cyclobakuchiols A (**2**), B (**3**), C (**4**), and D (**5**) (12.5 μM), DMSO (0.125%), or (+)-(*S*)-bakuchiol (**1**) (12.5 μM) were mixed with A/PR/8/34 virus (MOI 0.1) and incubated for 30 min prior to the addition to 1 × 10^5^ MDCK cells. (A–F) Total RNA was extracted from cell lysates 24 h post-infection. The relative expression levels of viral mRNAs [*NP* (A), *NS1* (B), *PA* (C), *PB1* (D), *PB2* (E) or *M2* (F)] (n = 9 each) were determined by RT-qPCR. These mRNA levels were normalized to 18s ribosomal RNA and expressed in relation to the levels in the DMSO-treated cells (set as 1). Data represent the mean ± SEM and are representative of three independent experiments. UI; uninfected cells. *p < 0.05, ***p < 0.001 for the indicated comparisons. Results were reproducible in this experiment.

To evaluate whether **2**–**5** inhibited the expression of influenza A viral mRNAs, RT-qPCR and western blotting in MDCK cells treated with **2**–**5** or **1** and infected with A/PR/8/34 virus and incubated for 24 h were performed. The relative expression levels of viral mRNAs *NP*, *NS1*, *PA*, *PB1*, *PB2*, and *M2* were determined by RT-qPCR (Fig 6). The relative mRNA expression levels of viral genes were found to be significantly decreased in MDCK cells treated with **2**–**5** or **1**, compared with those of the DMSO-treated cells (Fig 6). Additionally, the reduction in the levels of *NP* or *PB2* mRNA in cells treated with **2** or **1** were significantly stronger than those of **4** and **5** (Fig 6A) or **5** (Fig 6E), respectively.

**Fig 7:**
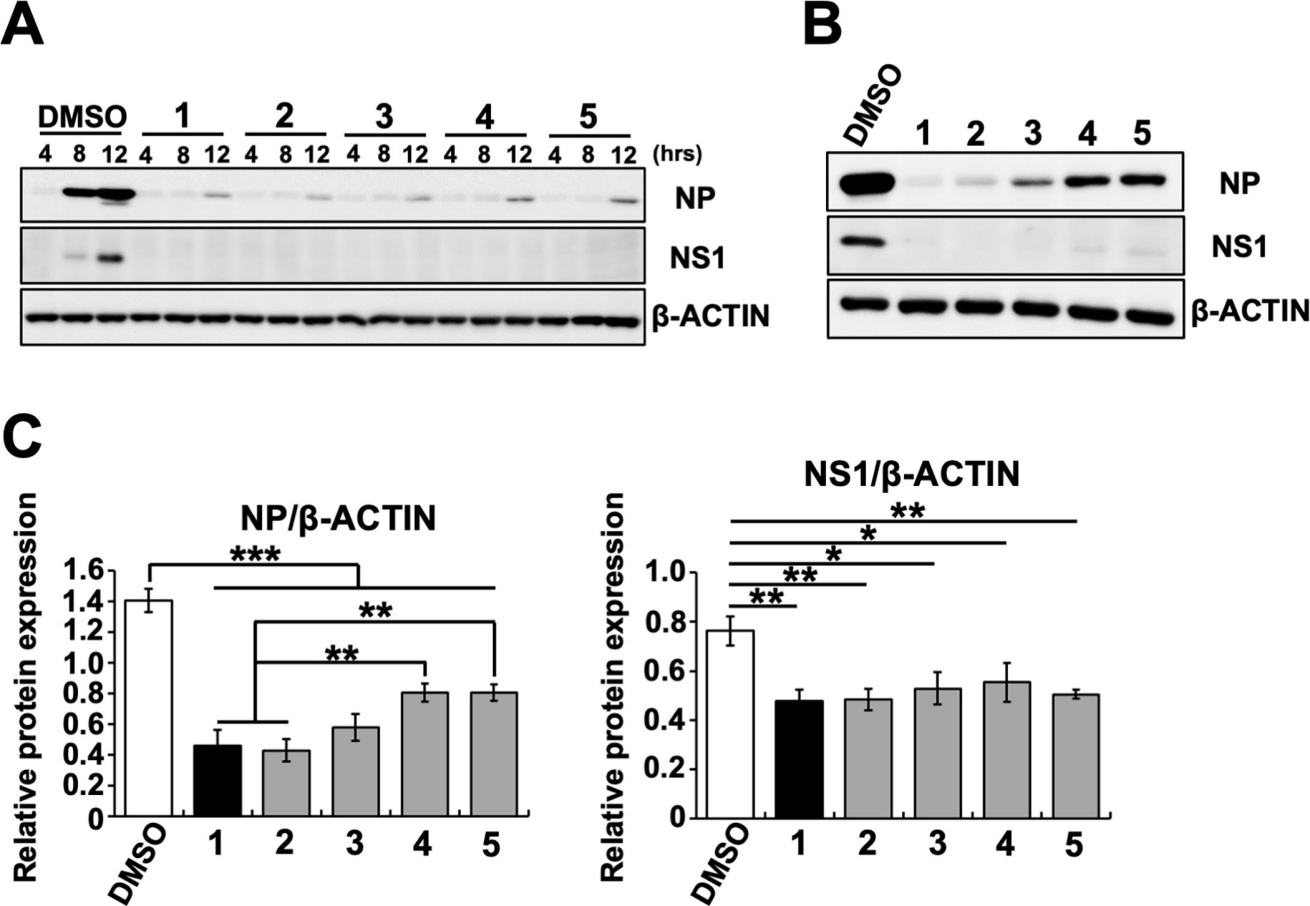
Cyclobakuchiols A–D inhibits the expression of influenza A viral proteins. Cyclobakuchiols A (**2**), B (**3**), C (**4**), and D (**5**) (12.5 μM), DMSO (0.125%), or (+)-(*S*)-bakuchiol (**1**) (12.5 μM) were mixed with A/PR/8/34 virus (MOI 0.1) and incubated for 30 min prior to the addition to 1 × 10^5^ MDCK cells. (A–C) The levels of influenza A viral NP and NS1 proteins in cell lysates were analyzed by western blotting at 4–12 h (A) or 24 h (B) post-infection. β-actin protein was analyzed as an internal control. Signal intensities at 24 h post-infection were measured using ImageJ software, and the protein levels of NP/β-actin or NS1/β-actin were analyzed, while the protein levels of NP (C, left panel) (n = 3 each) and NS1 (C, right panel) (n = 3 each) were normalized to that of β-actin. Data represent the mean ± SEM and are representative of three independent experiments. *p < 0.05, **p < 0.01, ***p < 0.001 for the indicated comparisons. The results were reproducible in this experiment.

Next, the inhibitory effect of **2**–**5** on influenza A viral protein expression was examined. **2**–**5** or **1** were mixed with A/PR/8/34 virus and then added to MDCK cells. The expression of influenza A viral NP and NS1 proteins in cell lysates was analyzed by western blotting after incubation for 4, 8, 12, and 24 h post-infection (Fig 7). As a result, the expression of NP and NS1 proteins was reduced in MDCK cells treated with **2**–**5** or **1** for 8, 12 (Fig 7A), or 24 h (Fig 7B), compared with those treated with DMSO. The relative expression levels of NP or NS1 proteins at 24 h post-infection (Fig 7B) were analyzed from these signal intensities (Fig 7C). The expression levels of NP or NS1 proteins in cells treated with **2**–**5** or **1** were significantly reduced compared with those in the DMSO-treated cells (Fig 7C). Additionally, the expression levels of NP protein in **2** or **1** were significantly reduced compared with those in the **4** or **5** treatment groups, respectively (Fig 7C, left panel). These results indicate that cyclobakuchiols A–D reduced the expression of influenza A viral mRNAs and proteins, an effect that appears to depend on the oxidation state of isopropyl group of the cyclobakuchiol structure.

### Cyclobakuchiols A–D reduced the mRNA expression of IFN-β and Mx1 genes in host cells following influenza A viral infection

**Fig 8:**
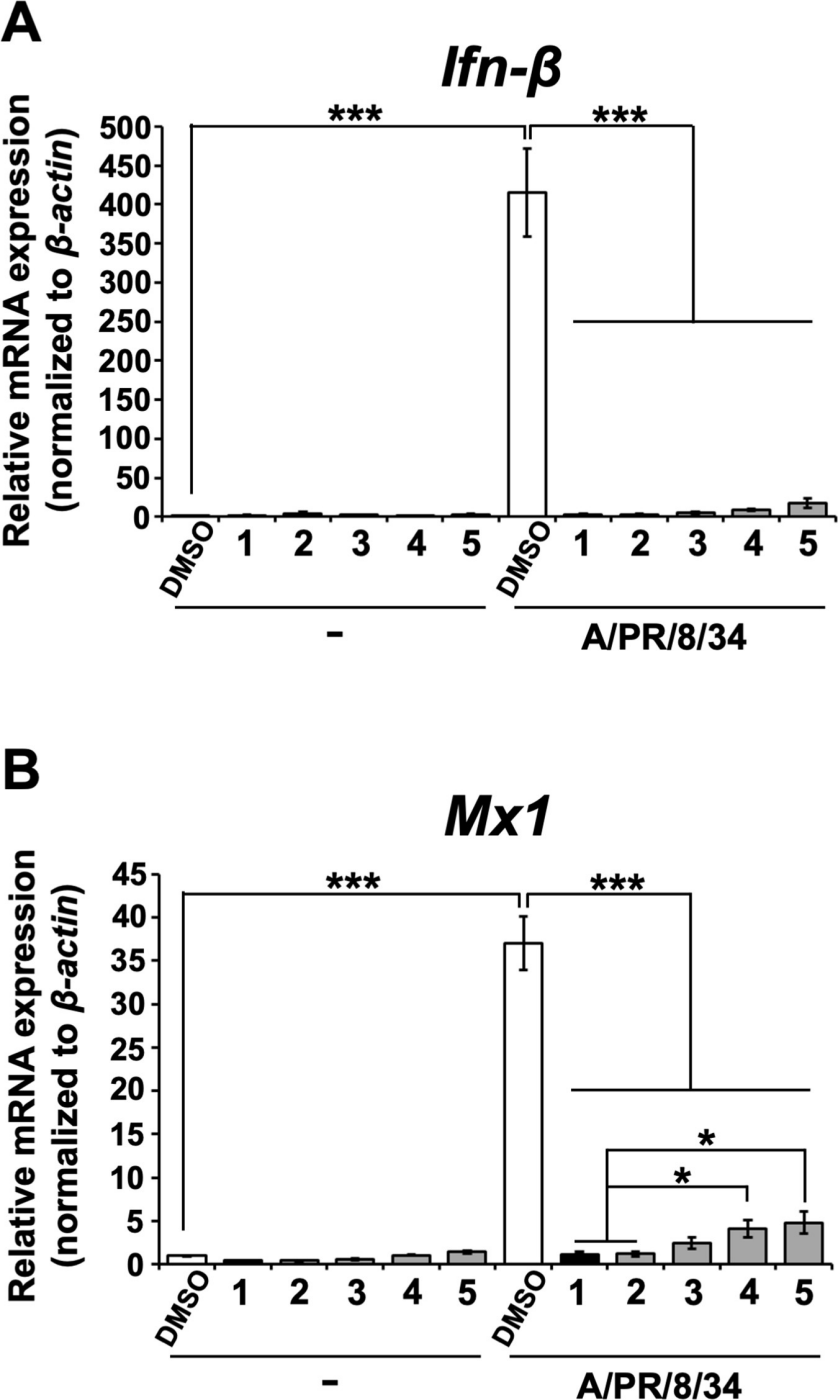
Cyclobakuchiols A–D inhibit the upregulation of *Ifn-β* and *Mx1* mRNAs in influenza A virus-infected cells. Cyclobakuchiols A (**2**), B (**3**), C (**4**), and D (**5**) (12.5 μM), DMSO (0.125%), or (+)-(*S*)-bakuchiol (**1**) (12.5μM) were mixed with or without A/PR/8/34 virus (MOI 0.1) and incubated for 30 min prior to the addition to 1 × 10^5^ MDCK cells. Total RNA was extracted from cell lysates 24 h post-infection. The relative levels of *Ifn-β* (n = 9 each) (A) or *Mx1* (n = 9 each) (B) mRNA were determined by RT-qPCR, normalized to *β-actin* mRNA, and expressed relative to the levels in DMSO-treated non-infected cells (set as 1). Data are presented as the mean ± SEM of three independent experiments. *p < 0.05, ***p < 0.001 for the indicated comparisons. The results were reproducible in this experiment.

IFN-β has been known as an antiviral cytokine in the innate immune response of host cells induced by influenza A viral infection and growth, while Mx1 is known to be an antiviral host cell protein upregulated by IFN-β [30–32]. We previously reported that **1** inhibits the mRNA expression of *Ifn-β* and *Mx1*, an Ifn-induced factor, in the innate immune response of host cells induced by influenza A viral infection and growth [23], indicating that **1** inhibits influenza A viral infection and growth. Based on our findings indicating that **2**–**5** inhibited influenza A viral infection and growth, we hypothesized that **2**–**5** would reduce the mRNA expression of *Ifn-β* and *Mx1* in host cells. Thus, the mRNA levels of *Ifn-β* and *Mx1* in the MDCK cells infected with A/PR/8/34 virus and treated with **2**–**5** or **1** by RT-qPCR were analyzed (Fig 8). The relative mRNA levels of *Ifn-β* (Fig 8A) and *Mx1* (Fig 8B) were found to be upregulated in cells infected with A/PR/8/34 virus and treated with DMSO. However, this upregulation was significantly reduced by treatment with **2**–**5** or **1** (Fig 8). Additionally, the mRNA levels of *Mx1* in cells treated with **2** or **1** were significantly reduced compared with those of **4** or **5**-treatment, respectively (Fig 8B). Taken together, these results provide evidence for the suppression of the innate immune response of host cells induced by influenza A viral infection and growth by cyclobakuchiols A–D, demonstrating that these compounds inhibit influenza A viral infection and growth.

### Cyclobakuchiols A–D upregulated Nqo1 mRNA, and cyclobakuchiols A–C induced Nrf2-activation

In a previous study, the molecular pathways of intracellular activation by **1** (using KeyMolnet and NGS results) were analyzed and found that **1** activated the Nrf pathway [23]. In addition, it has been reported that the mRNA expression of *NQO1* is regulated by the Nrf2 transcription factor and is related to the cellular response to oxidative stress [33–36].

**Fig 9:**
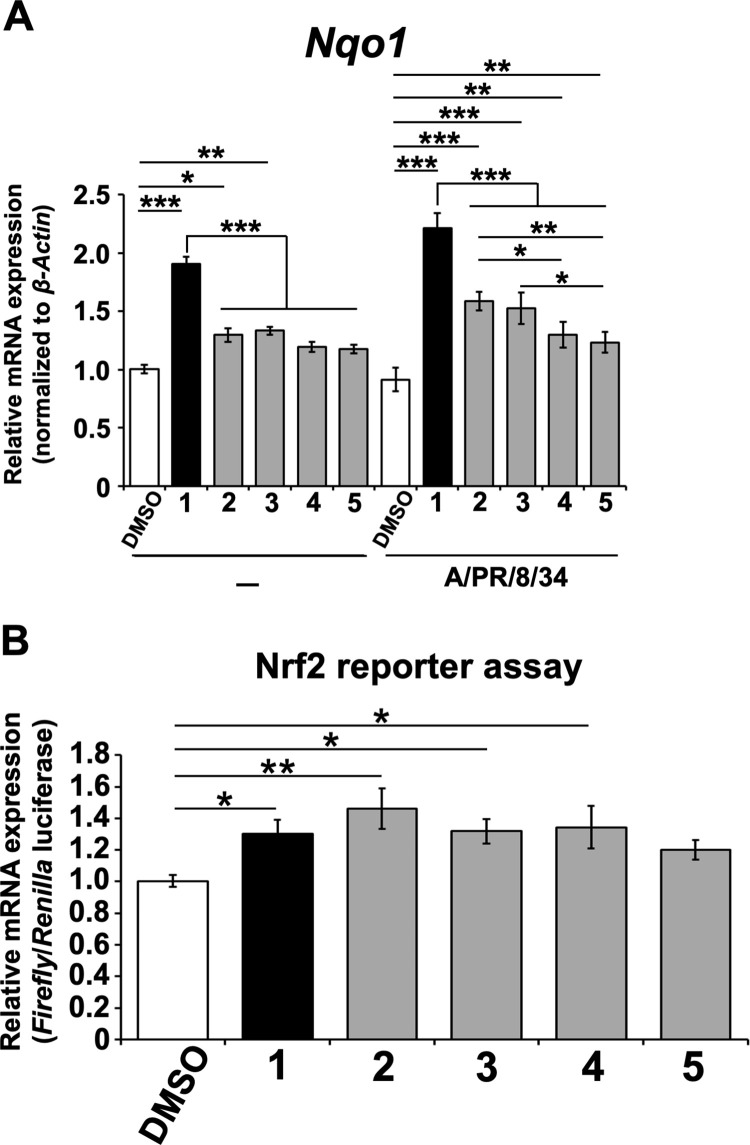
Cyclobakuchiols A–D increase the mRNA expression of NAD(P)H quinone oxidoreductase 1 in MDCK cells, and cyclobakuchiols A–C induce the nuclear factor erythroid 2-related factor 2 activation. (A) Cyclobakuchiols A (**2**), B (**3**), C (**4**), and D (**5**) (12.5 μM), DMSO (0.125%), or (+)-(*S*)-bakuchiol (**1**) (12.5 μM) were mixed with or without A/PR/8/34 virus (MOI 0.1) and added to 1 × 10^5^ MDCK cells for 24 h. Total RNA was extracted from the cell lysates, and the mRNA levels of NAD(P)H quinone oxidoreductase 1 (*Nqo1*), a nuclear factor erythroid 2-related factor 2 (Nrf2)-induced gene (n = 9 each), were determined by RT-qPCR, normalized to *β-actin* mRNA, and expressed relative to the DMSO-treated non-infected cells (set as 1). (B) A Nrf2 reporter assay based on the dual luciferase system was performed in MDCK cells. MDCK cells (1 × 10^5^) were transfected with pNQO1-ARE-Fluc, expressing a *Firefly* luciferase gene driven by Nrf2 activation, and pRL-TK-Rluc, expressing *Renilla* luciferase driven by the herpes simplex viral thymidine kinase promoter. At 24 h post-transfection, the cells were treated with **2**–**5** (12.5 μM) (n = 6 each), DMSO (0.125%) (n = 6), or **1** (12.5μM) (n = 6). The levels of *Firefly* and *Renilla* luciferase mRNA were analyzed by RT-qPCR after 24 h and normalized to *β-actin* mRNA. The relative levels of *Firefly* per *Renilla* luciferase mRNAs were calculated and compared with that observed in the DMSO-treated cells (set as 1) and expressed relative to the DMSO-treated cells (set as 1). Data are presented as the mean ± SEM of three independent experiments. *p < 0.05, **p < 0.01, ***p < 0.001 for the indicated comparisons. The results were reproducible across all experiments.

Thus, the upregulation of *Nqo1* mRNA in MDCK cells infected with or without A/PR/8/34 virus and treated with **2**–**5** or **1** by RT-qPCR was examined (Fig 9A). The relative mRNA levels of *Nqo1* in cells treated with **2**–**5** or **1** and infected with A/PR/8/34 virus were significantly upregulated, whereas the relative mRNA levels of *Nqo1* in cells treated with **2** and **3** or **1** without A/PR/8/34 virus were significantly upregulated, compared with those of DMSO-treated cells (Fig 9A). The relative mRNA levels of *Nqo1* in cells treated with **2**–**5** and infected with or without A/PR/8/34 virus were significantly lower than those treated with **1**. Additionally, the relative mRNA levels of *Nqo1* in cells infected with A/PR/8/34 virus and treated with **2** or **3** were significantly higher than those in the **4**- and **5**-treated or **5**-treated cells, respectively (Fig 9A). Next, it was analyzed whether **2**–**5** induced Nrf2 activation in MDCK cells using the Nrf2 reporter assay (Fig 9B). As a result, treatment with **2**–**4** or **1**, but not **5**, was found to induce Nrf2 activation, in contrast to DMSO treatment (Fig 9B).

Taken together, these results indicate that cyclobakuchiols A–D activate the Nrf2 pathway, and their effect depends on the existence of oxidized isopropyl group in the structure of cyclobakuchiol.

## Discussion

In the present study, cyclobakuchiols A–D enhanced the survival of influenza A virus-infected MDCK cells and inhibited influenza A viral infection and growth, as well as reduced the expression of viral mRNAs and proteins in influenza A virus-infected cells. Cyclobakuchiols A–D reduced the expression of influenza A virus-induced immune response genes in host cells. Cyclobakuchiols A–D also upregulated *Nqo1* mRNAs in influenza A-infected cells. Cyclobakuchiols A–C, but not cyclobakuchiol D, were found to induce Nrf2 activation in host cells, indicating that their effect depends on the oxidation state of isopropyl group in the structure of the cyclobakuchiol. This study is the first to demonstrate that cyclobakuchiols A–D have anti-influenza viral activity involving the host cell oxidative stress response and that their effect is dependent on stereoisomers and structural isomers. These results demonstrated that cyclobakuchiols have anti-influenza virus activity, where the strength of this effect depends on the structure of the cyclobakuchiol. Cyclobakuchiol A and B or cyclobakuchiol C and D are diastereomers, and cyclobakuchiol A and C or cyclobakuchiol B and D are structural isomers, respectively (Fig 1). The anti-influenza virus activity of cyclobakuchiols is in the order of cyclobakuchiols A > B > C > D, suggesting that the chirality of the oxidized isopropyl group and its attached carbon is important (Figs 3–9). Sekine et al. reported that the presence and difference of the isopropyl group in a ring of *p*-menthane monoterpenoids affected termite mortality [37]. Several studies have reported that the isopropyl group in the compound has biological effects, such as anti-mycobacterial [38,39], anti-amoebic [40], and anticancer [39] activities. Therefore, the isopropyl group arrangement is important for the pharmacological activity of the compounds. These results indicate that the suitably isopropyl group of cyclobakuchiols may be required for their anti-influenza virus activity.

The results of RT-qPCR analyses and Nrf2 reporter assays in MDCK cells treated with cyclobakuchiols and infected with A/PR/8/34 virus showed that cyclobakuchiols A–D upregulated the *Nqo1* mRNA levels and cyclobakuchiols A–C induced Nrf2 activation, in comparison with DMSO-treated cells (Fig 9). In a previous study, we found that bakuchiol upregulates *Nqo1* mRNA and activates Nrf2 in MDCK cells [23]. NQO1 catalyzes the reduction of various quinones involving NADH or NADPH, preventing the formation of free radicals and reactive oxygen species (ROS). An increase in ROS levels activates Nrf2 binding to the NQO1 promoter, increasing NQO1 production [41]. Additionally, Chen et al. found that bakuchiol increased p53 expression and induced apoptosis via ROS-dependent reduction of mitochondrial membrane potential in A549 cells [22]. Therefore, the upregulation of *Nqo1* mRNA by cyclobakuchiols is induced by ROS-dependent Nrf2 activation, resulting in an increase in the level of p53 protein in MDCK cells. Nrf2 activation has been shown to reduce influenza A viral entry and replication [42], and the inhibition of p53 expression increases influenza A viral growth [43], suggesting that the upregulation of Nrf2 and p53 inhibits influenza A viral growth. Therefore, Nrf2 activation could represent a mechanism for the anti-influenza A virus H1N1 of cyclobakuchiols. Furthermore, cyclobakuchiol D inhibited influenza A viral infection and growth while upregulating the *Nqo1* mRNA levels, but did not induce Nrf2 activation (Figs 3–9), suggesting that cyclobakuchiols have additional mechanisms of anti-influenza A activity.

Our pre-incubation experiments showed that cyclobakuchiols A–D inhibited the H1N1 strains of influenza virus A/PR/8/34 and A/CA/7/09, but weakly inhibited the H1N1 strain A/WSN/33 virus. This reflects potential differences in viral strain proteins or in the host cell response. The H1N1 strain has various subtypes depending on the amino acid sequence. The WSN strain was established by adapting the A/WSN/33 virus strain isolated from humans to various hosts, followed by the subculture of the intracerebral inoculation of mice [44,45]. In general, differences in HA and NA amino acid sequences between A/WSN/33 and A/PR/8/34 viruses have been reported [46–48]. Although the amino acid sequences are similar, several different amino acid sequences are suspected of being involved in influenza virus specificity. In a previous study, we found that bakuchiol did not inhibit the hemagglutination of chicken red blood cells by A/PR/8/34 or A/CA/7/09 viral HAs, the trypsin digestion of recombinant A/PR/8/34 viral HA protein, or the activities of A/PR/8/34 or A/CA/7/09 viral NAs [23]. Cyclobakuchiols A–D also upregulated *Nqo1* mRNAs in influenza A-infected cells, and cyclobakuchiols A–C, but not cyclobakuchiol D, induced Nrf2 activation in host cells. Therefore, it is necessary to investigate the target factors of cyclobakuchiol, such as the host cell oxidative stress response, based on differences in the amino acid sequences of viral HA and/or NA proteins, which do not involve their activities, between A/PR/8/34 or A/CA/7/09 and A/WSN/33 viruses.

Bakuchiol has been recently reported to exhibit cytotoxic activity against several specific human cancer cell lines [49] while being physically bound to Hck, Blk, and p38 mitogen-activated protein kinase (MAPK), indicating that Hck, Blk, and p38 MAPKs are molecular targets for bakuchiol in skin cancer [50]. In addition, Long et al. reported that, in bakuchiol treatment, PI3K/AKT and MAPK are involved in bakuchiol-induced apoptosis, which leads to increased phosphorylation levels of ERK, JNK and p38 expression in gastric cancer cells [51]. These findings suggest that Nrf2-upstream effector proteins are targets for the anti-cancer activity of bakuchiol, and thus could be considered as targets of bakuchiol or cyclobakuchiols in anti-influenza virus activity. However, the direct target factors of bakuchiol and cyclobakuchiols in their anti-influenza A virus activity remain to be fully elucidated, thus further research will be needed.

## Conclusion

The findings presented in this study demonstrate that cyclobakuchiols A–D inhibit influenza A viral infection and growth in MDCK cells, as well as reducing the expression of viral mRNAs and proteins. Cyclobakuchiols A–D were found to reduce the induction of *Ifn-β* and *Mx1* mRNAs by influenza A virus. RT-qPCR analyses showed that cyclobakuchiols A–D upregulated *Nqo1* mRNAs in influenza A-infected cells, while cyclobakuchiols A–C, but not cyclobakuchiol D, induced Nrf2 activation in host cells. This effect was found to depend on the state of isopropyl group in the structure of cyclobakuchiol. This is the first report to demonstrate that cyclobakuchiols A–D have anti-influenza viral activity involving the host cell oxidative stress response, and that this effect is dependent on stereoisomers and structural isomers. The anti-influenza virus activity of cyclobakuchiols was found to be in the order of cyclobakuchiols A > B > C > D, suggesting that the chirality of oxidized isopropyl group and its attached carbon play an important role. This discovery provides important insights into the structure of compounds that can be used for the development of influenza therapeutics targeting the Nrf2 pathway in host cells. Thus, these results contribute to the determination of the structure of compounds for use in the development of influenza therapeutics.

## Supporting information

S1 Fig ^1^H-NMR and ^13^C-NMR spectra of cyclobakuchiols A–D. (PDF)

S1 Table Quantitative real-time PCR primer sequences. (XLSX)

S1 File Fig 2. (ZIP)

S2 File Fig 3 ABC raw data. (ZIP)

S3 File Fig 4 cyclobakuchiol MDCK PR8 0.1MOI IFA #1,3,4 raw data. (XLSX)

S4 File Fig 4 cyclobakuchiol MDCK WSN 0.1MOI IFA #1–3 raw data. (XLSX)

S5 File Fig 4 PR8 raw data tif files. (ZIP)

S6 File Fig 4 WSN raw data tif files. (ZIP)

S7 File Fig 5 cyclobakuchiol viral growth Titer. (ZIP)

S8 File Fig 6 Cyclobakuchiol MDCK PR8 24h qPCR #1–3 raw data. (XLSX)

S9 File Fig 7 cyclobakuchiol A-D MDCK PR8 24h WB #1–3 raw data. (XLSX)

S10 File Fig 7 cyclobakuchiol PR8 WB raw data. (ZIP)

S11 File Fig 8 cyclobakuchiol raw data. (XLSX)

S12 File Fig 9 cyclobakuchiol NQO1 qPCR + Nrf2 assay. (ZIP)
