# Supplementary material for: Organic synthesis and anti-influenza A virus activity of cyclobakuchiols A, B, C, and D
Source: PLoS One. 2021 Mar 26;16(3):e0248960. doi: 10.1371/journal.pone.0248960 (PMC7997032; doi:10.1371/journal.pone.0248960)

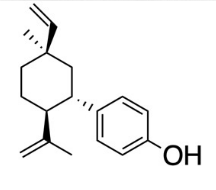

cyclobakuchiol A (**2**)

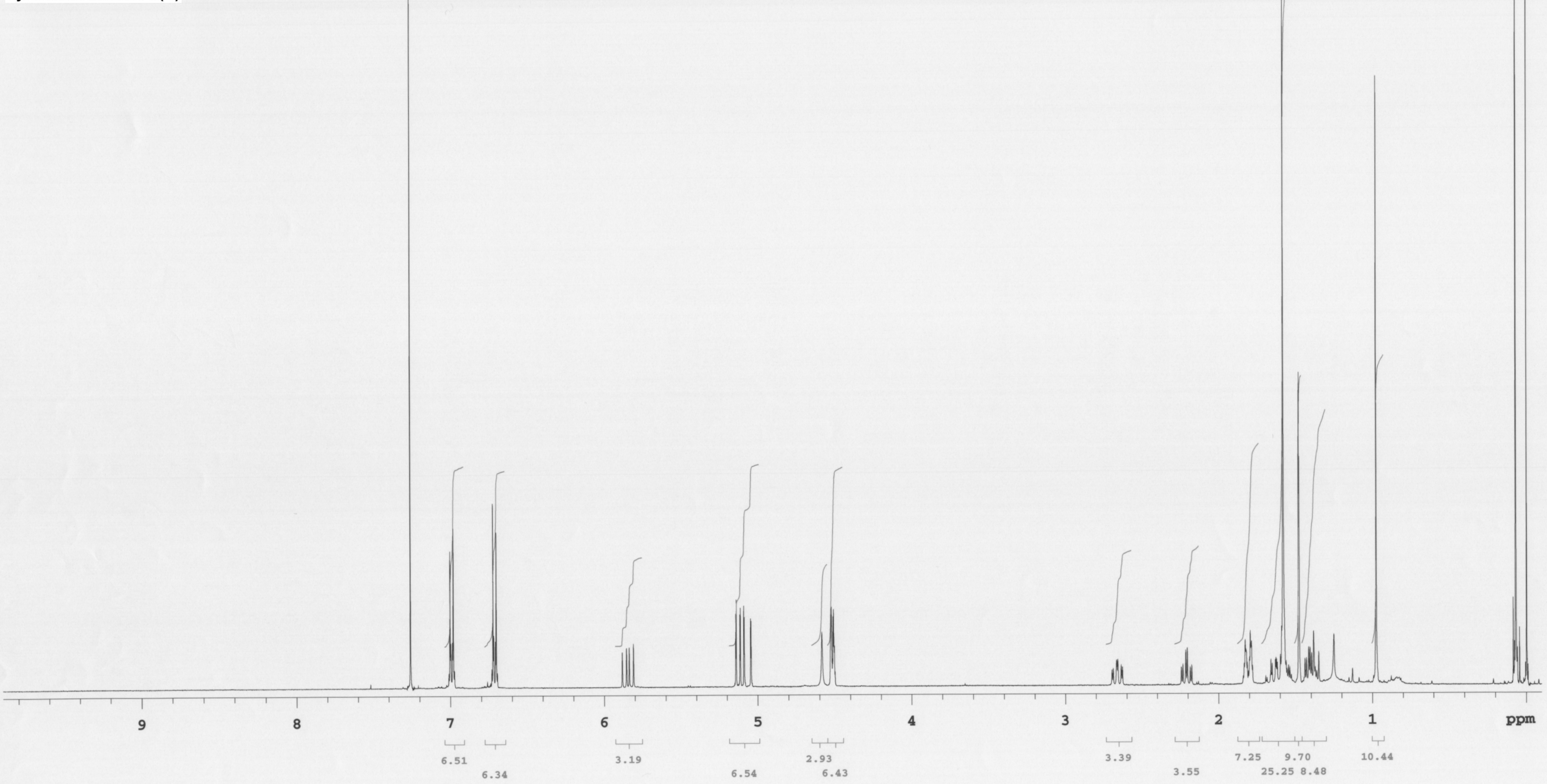

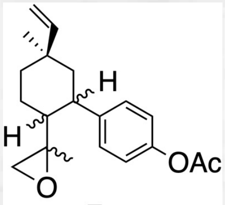
H
H
O
OAc

7

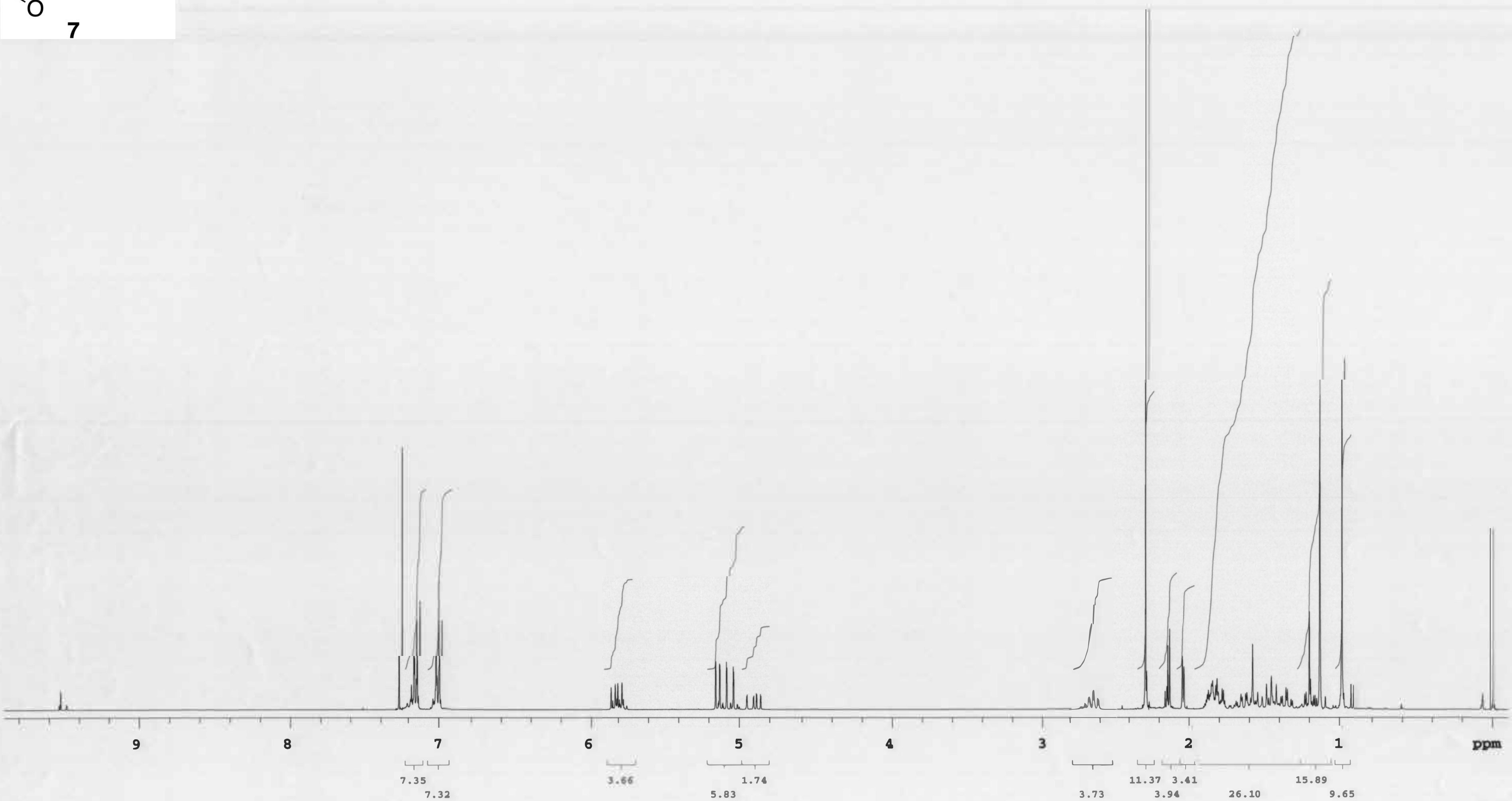
9
8
7
6
5
4
3
2
1
ppm
7.35
7.32
3.66
5.83
1.74
3.73
11.37
3.94
3.41
26.10
15.89
9.65

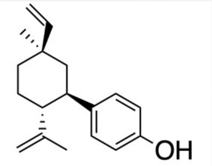

cyclobakuchiol B (**3**)

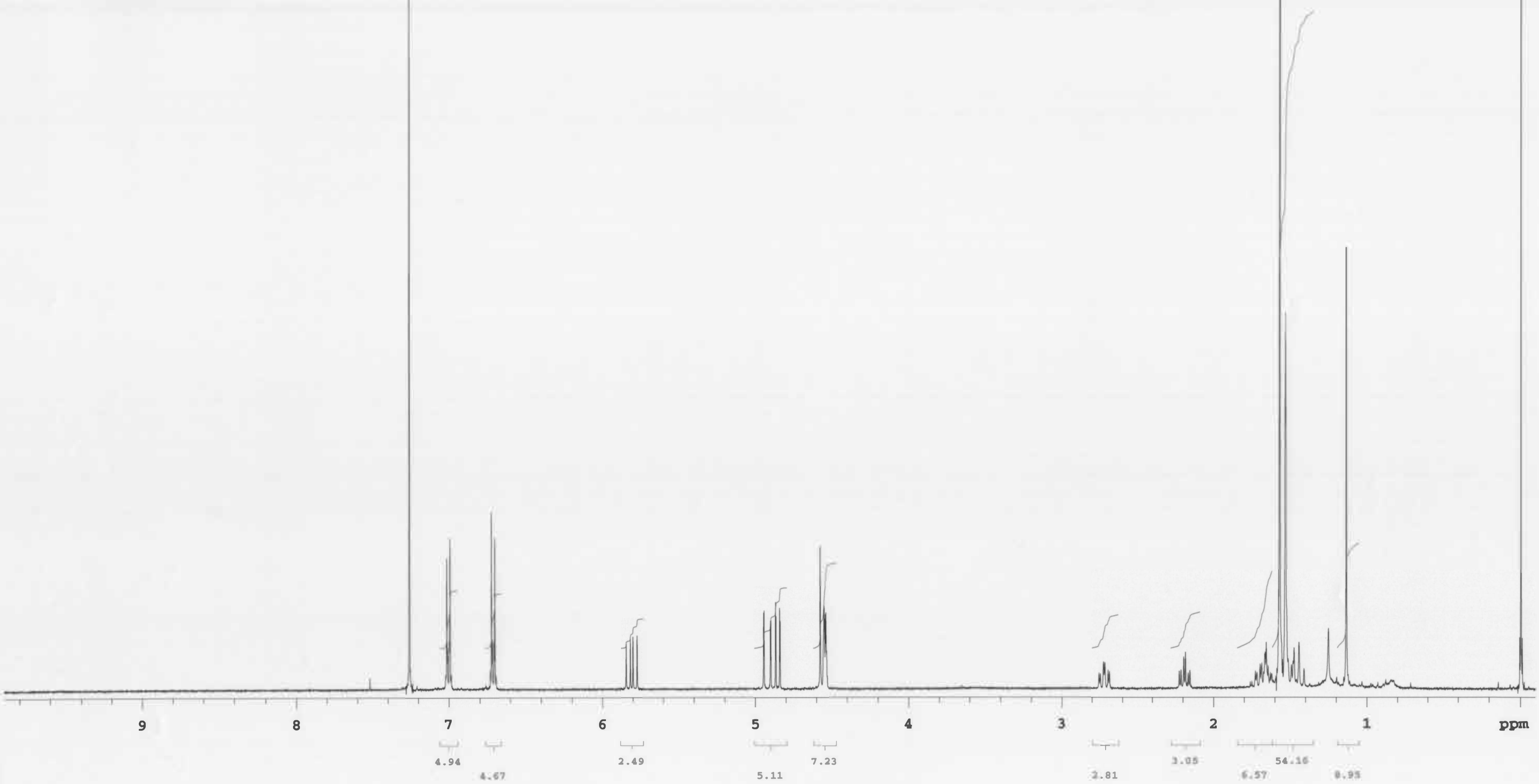

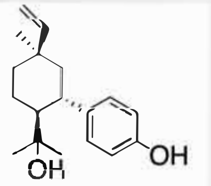

cyclobakuchiol C (**4**)

in $CDCl_3$

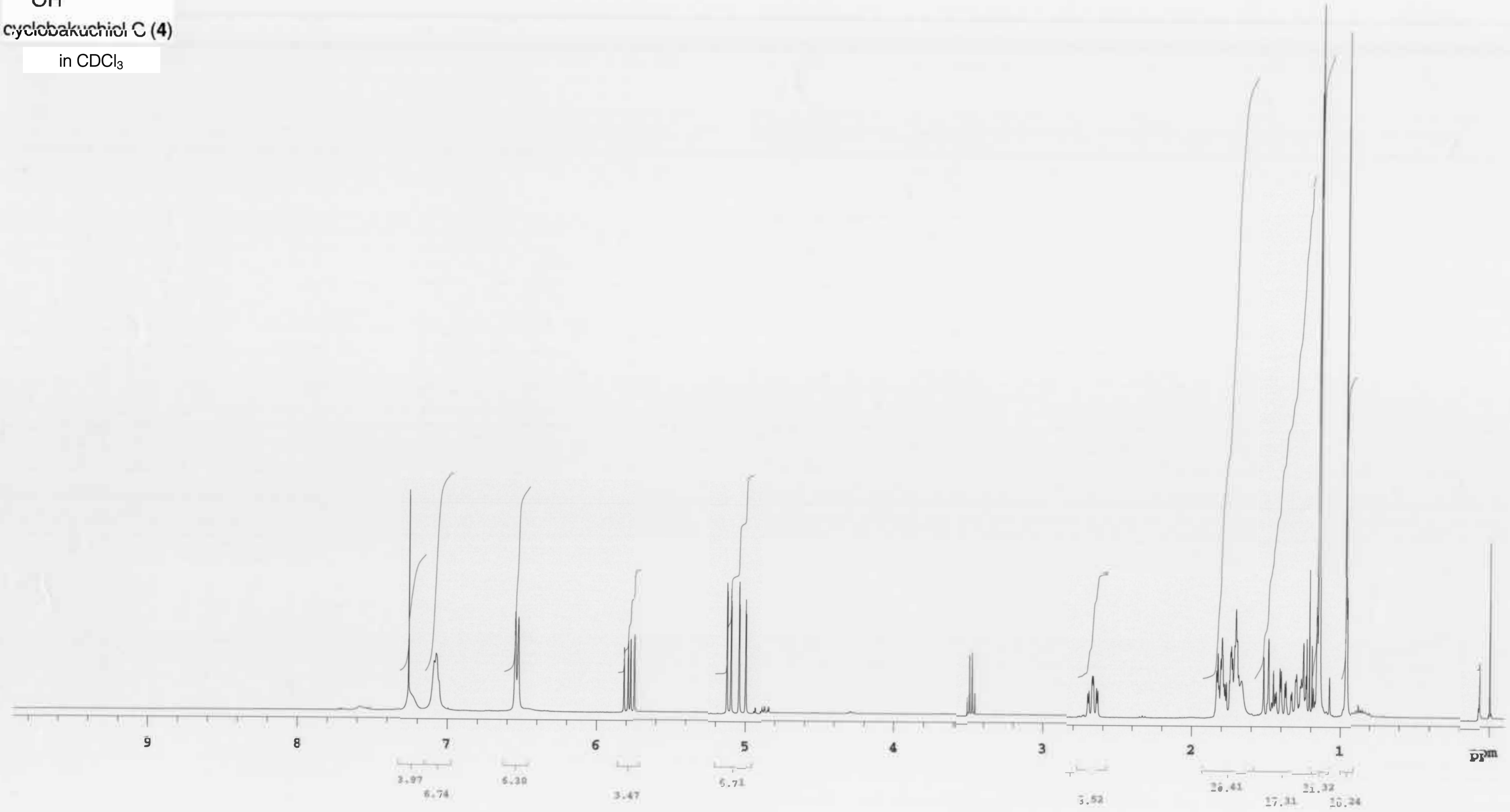

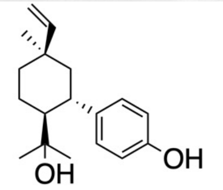
cyclobakuchiol C (**4**)
in CD_3OD

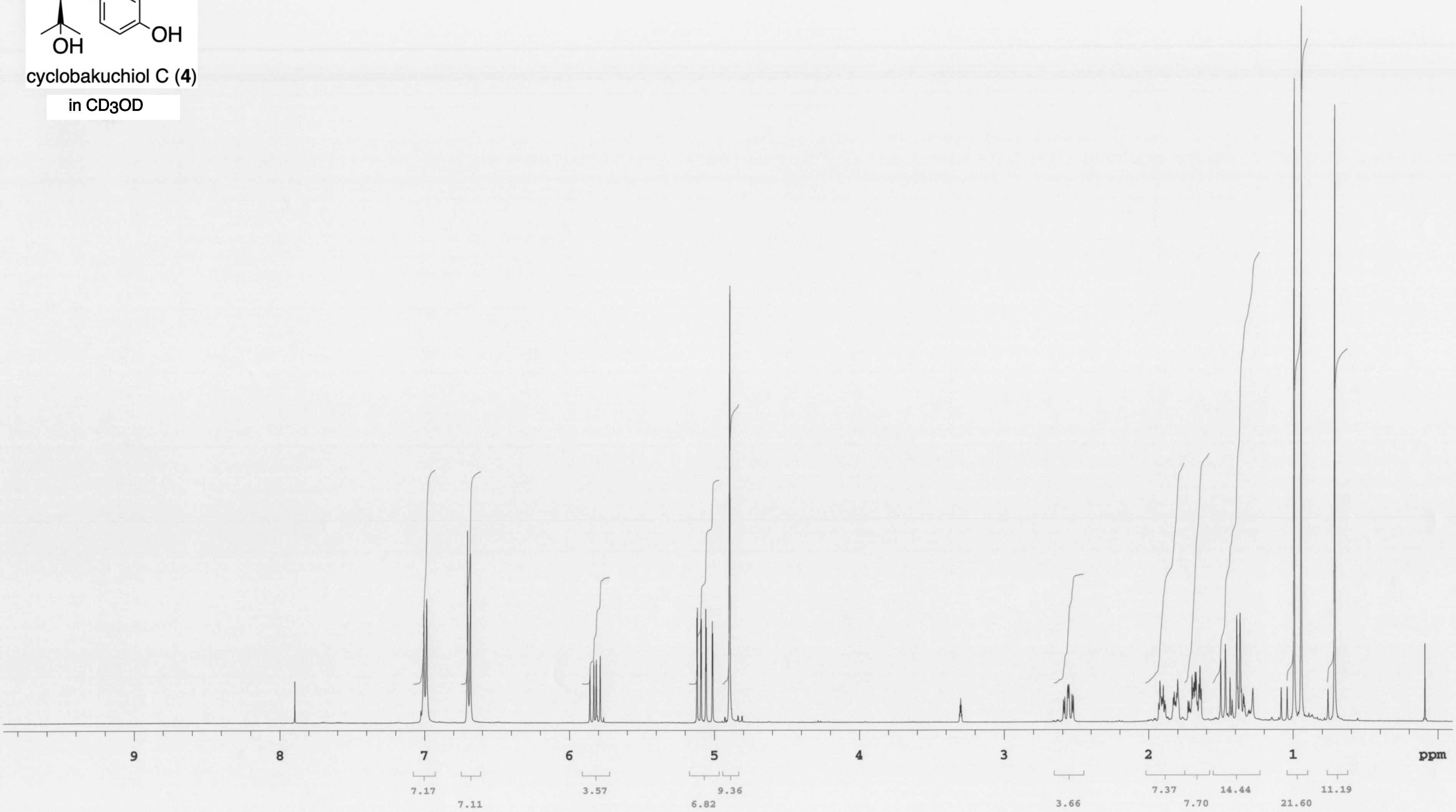

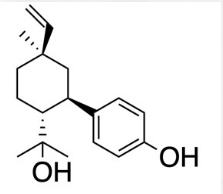

cyclobakuchiol D (**5**)

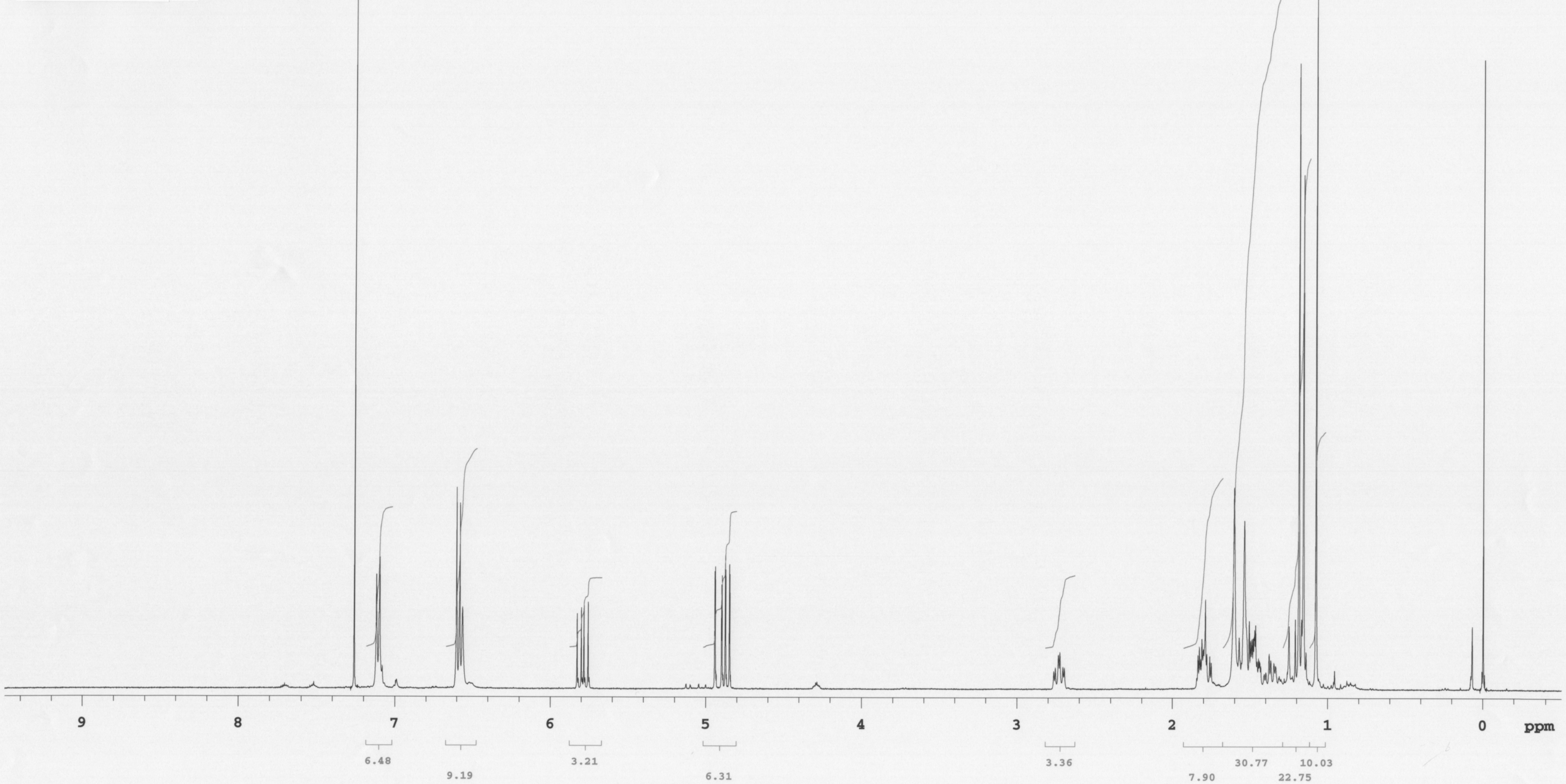

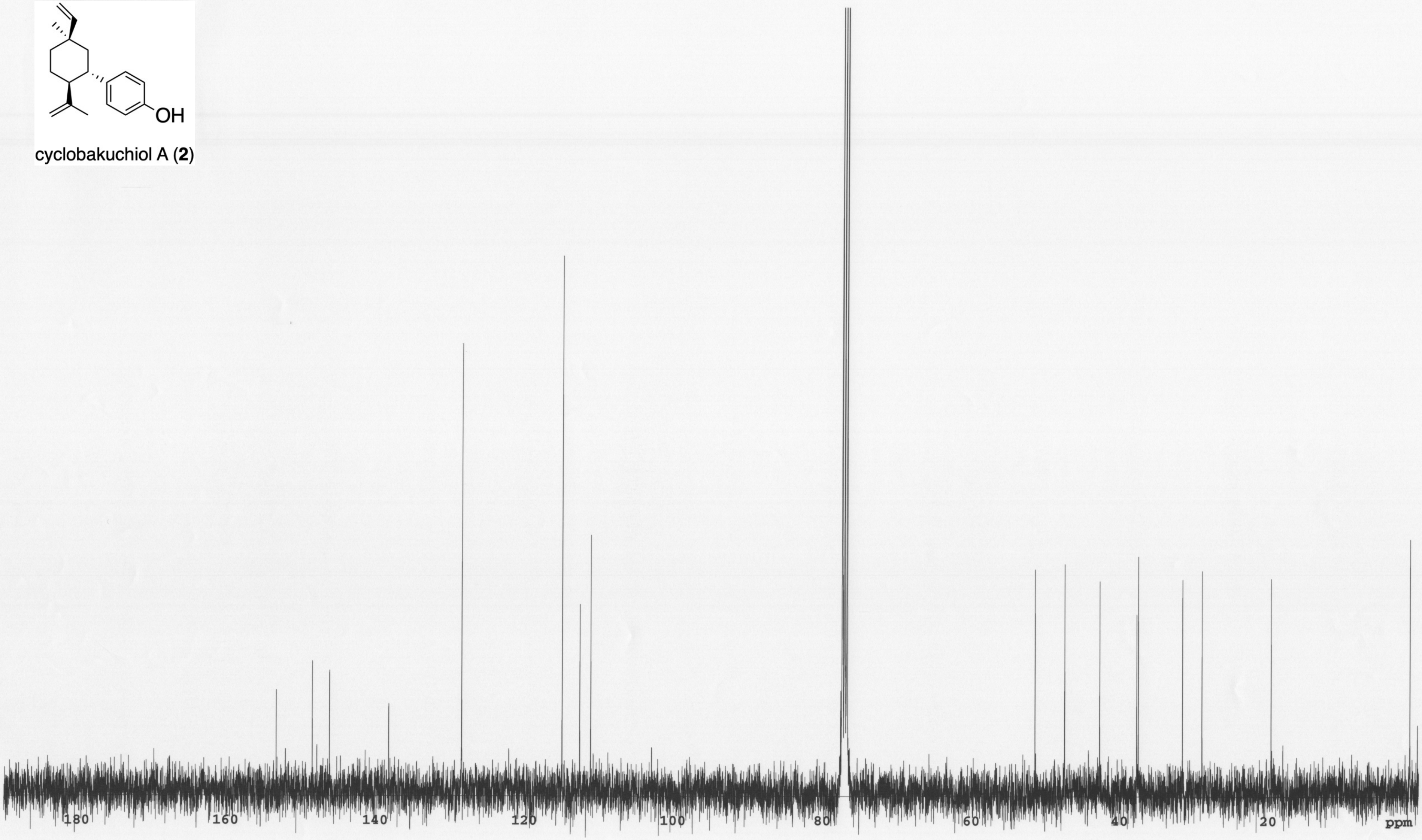

cyclobakuchiol A (**2**)
180
160
140
120
100
80
60
40
20
ppm

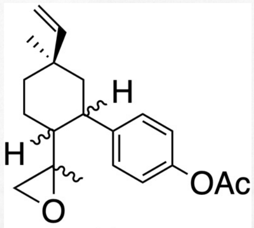

7

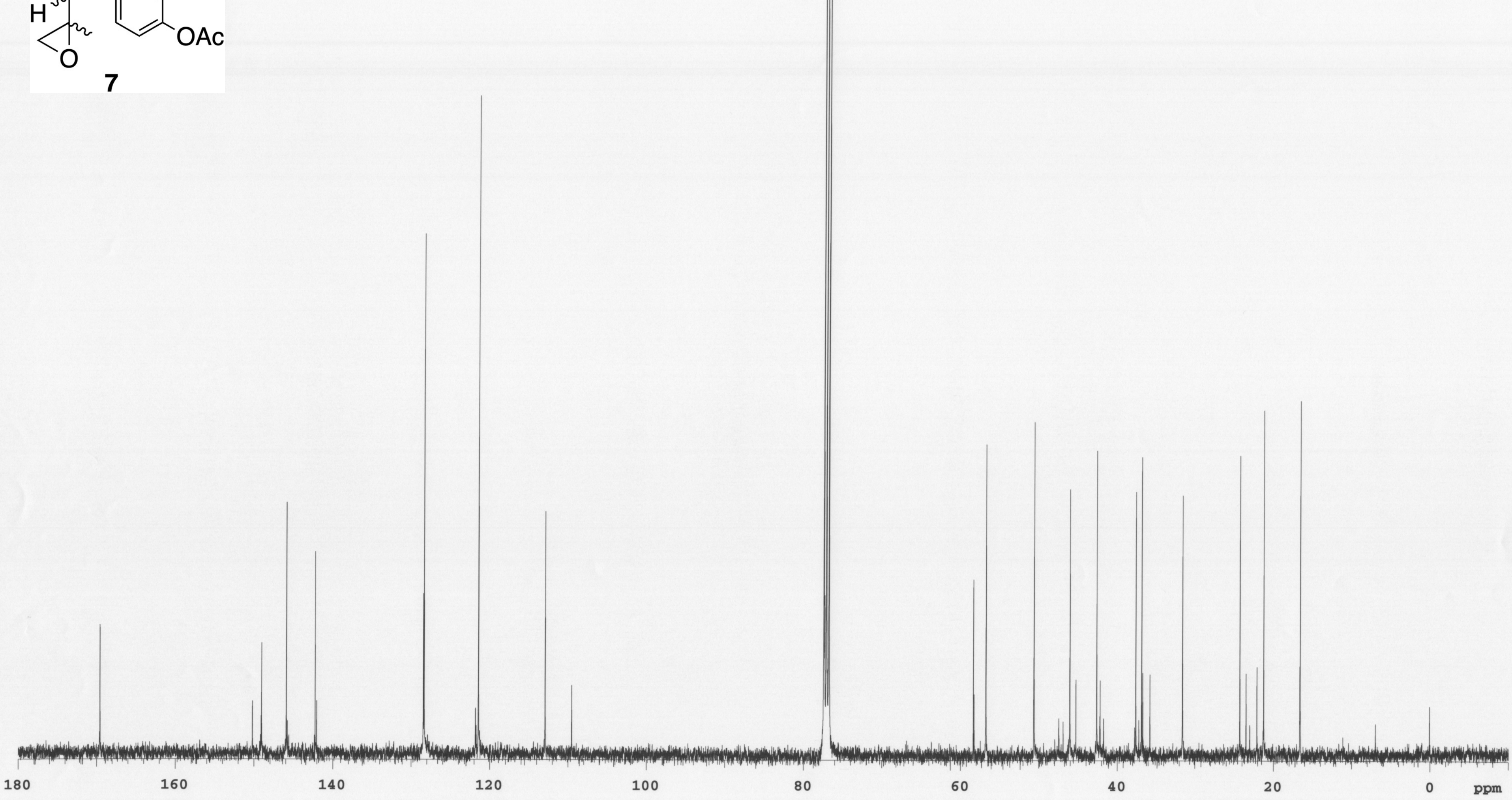

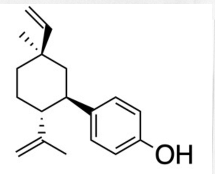

cyclobakuchiol B (**3**)

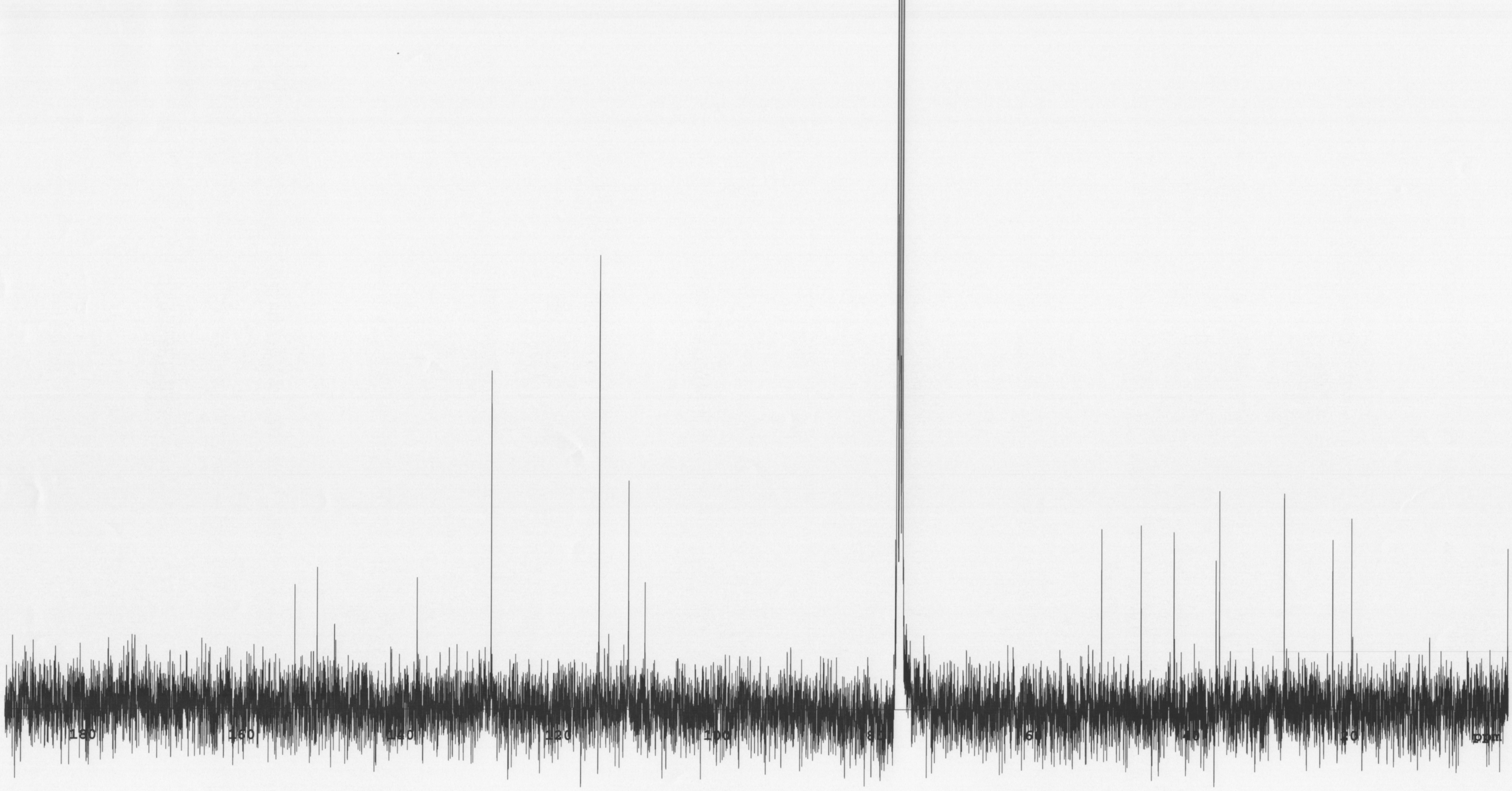

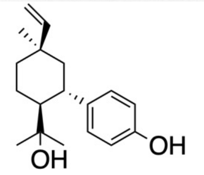

cyclobakuchiol C (**4**)

in $CDCl_3$

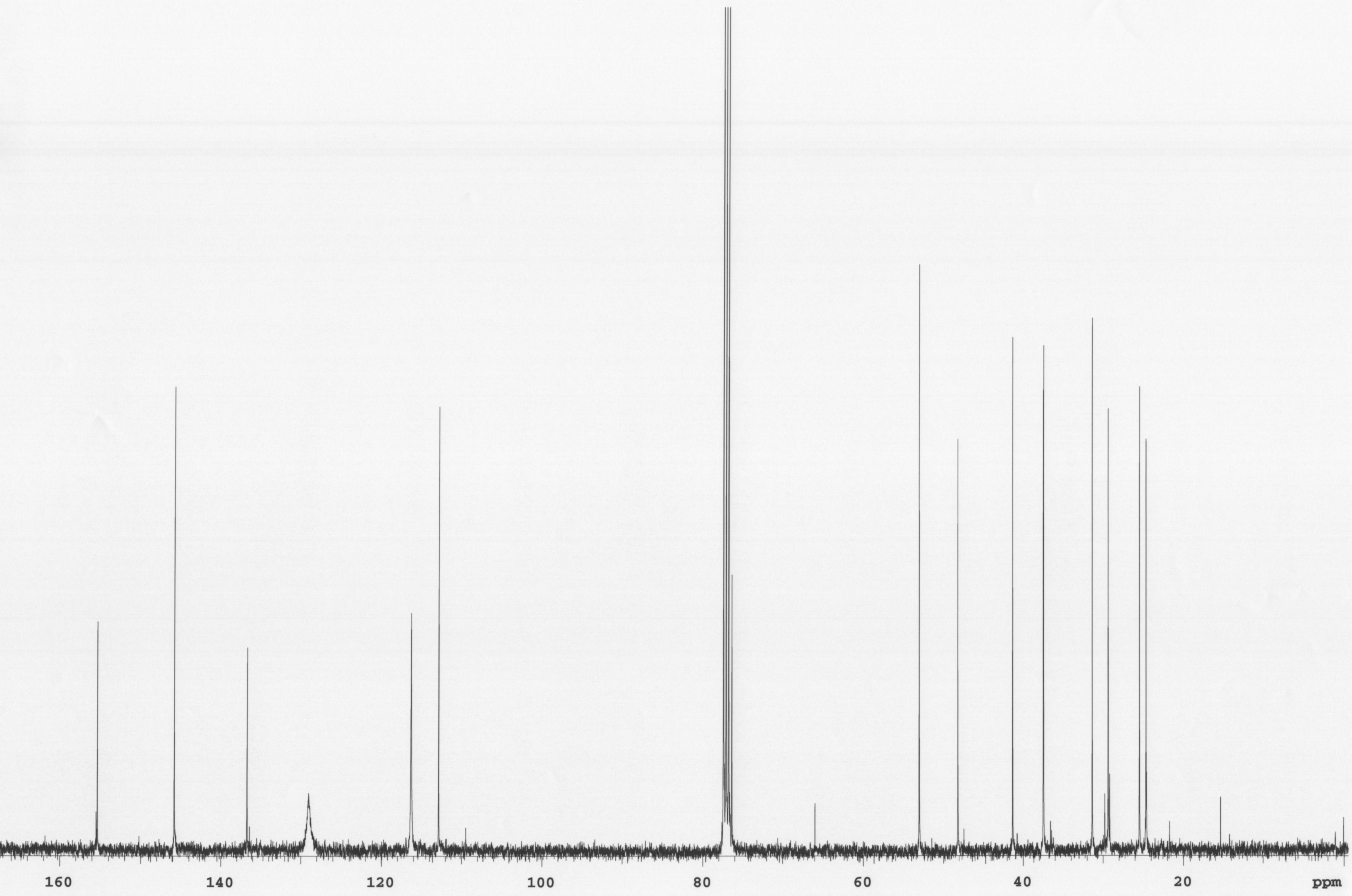

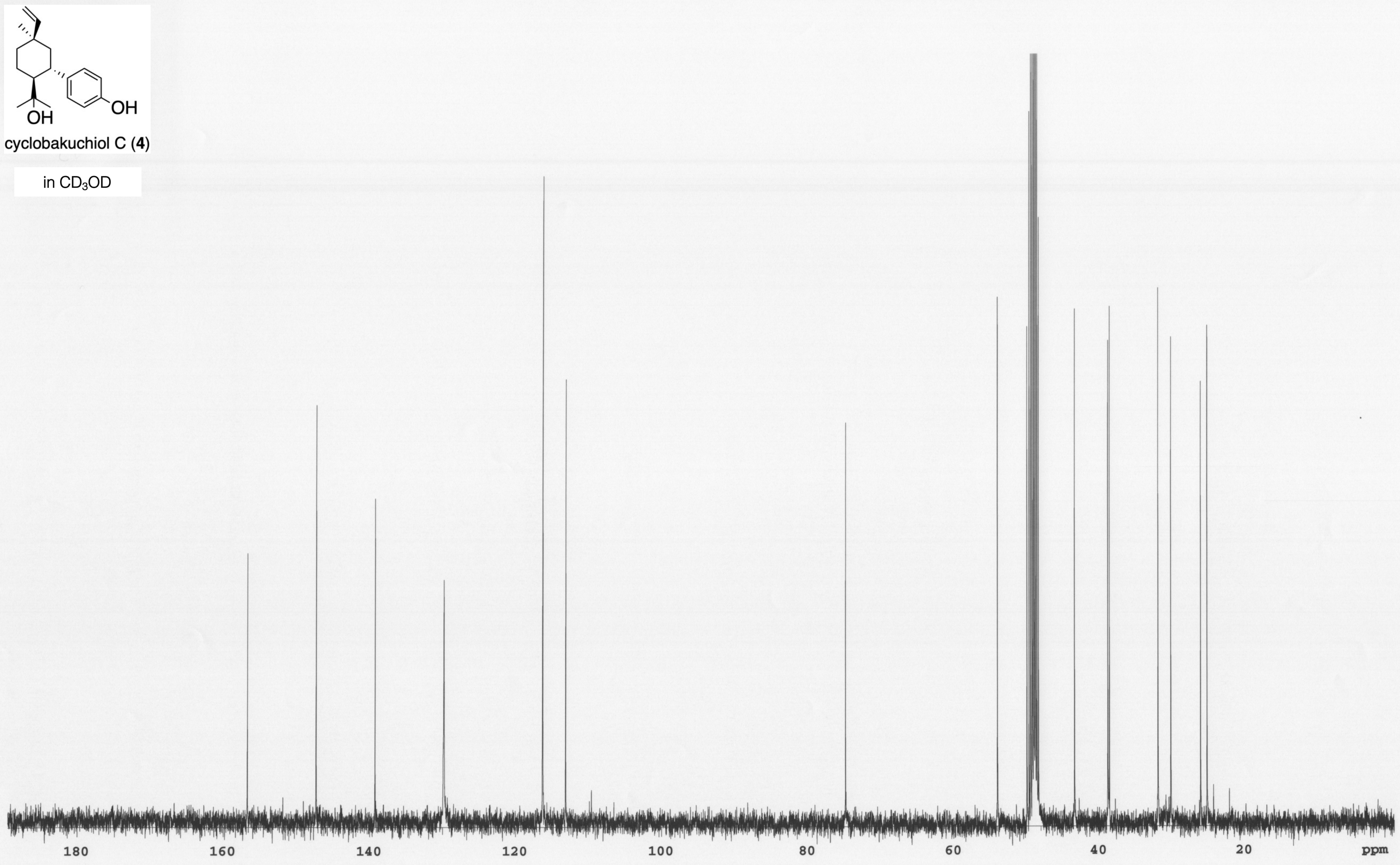

OH
OH
cyclobakuchiol C (4)
in CD3OD
180
160
140
120
100
80
60
40
20
ppm

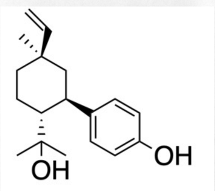

cyclobakuchiol D (**5**)

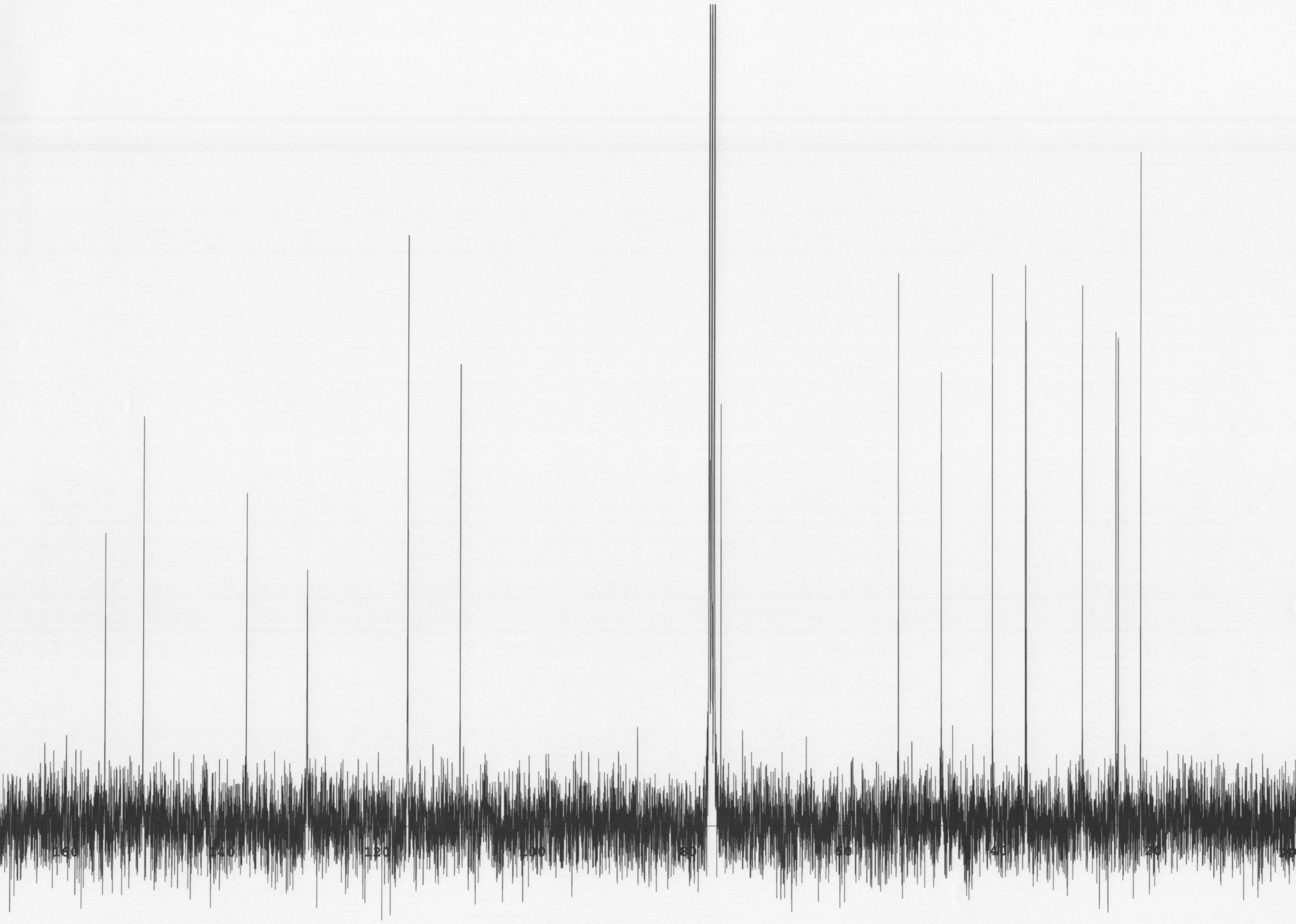

Supplement: S1 Fig — (PDF) [file pone.0248960.s001.pdf]
