# Supplementary figures and images for: Organic synthesis and anti-influenza A virus activity of cyclobakuchiols A, B, C, and D
Source: PLoS One. 2021 Mar 26;16(3):e0248960. doi: 10.1371/journal.pone.0248960 (PMC7997032; doi:10.1371/journal.pone.0248960)

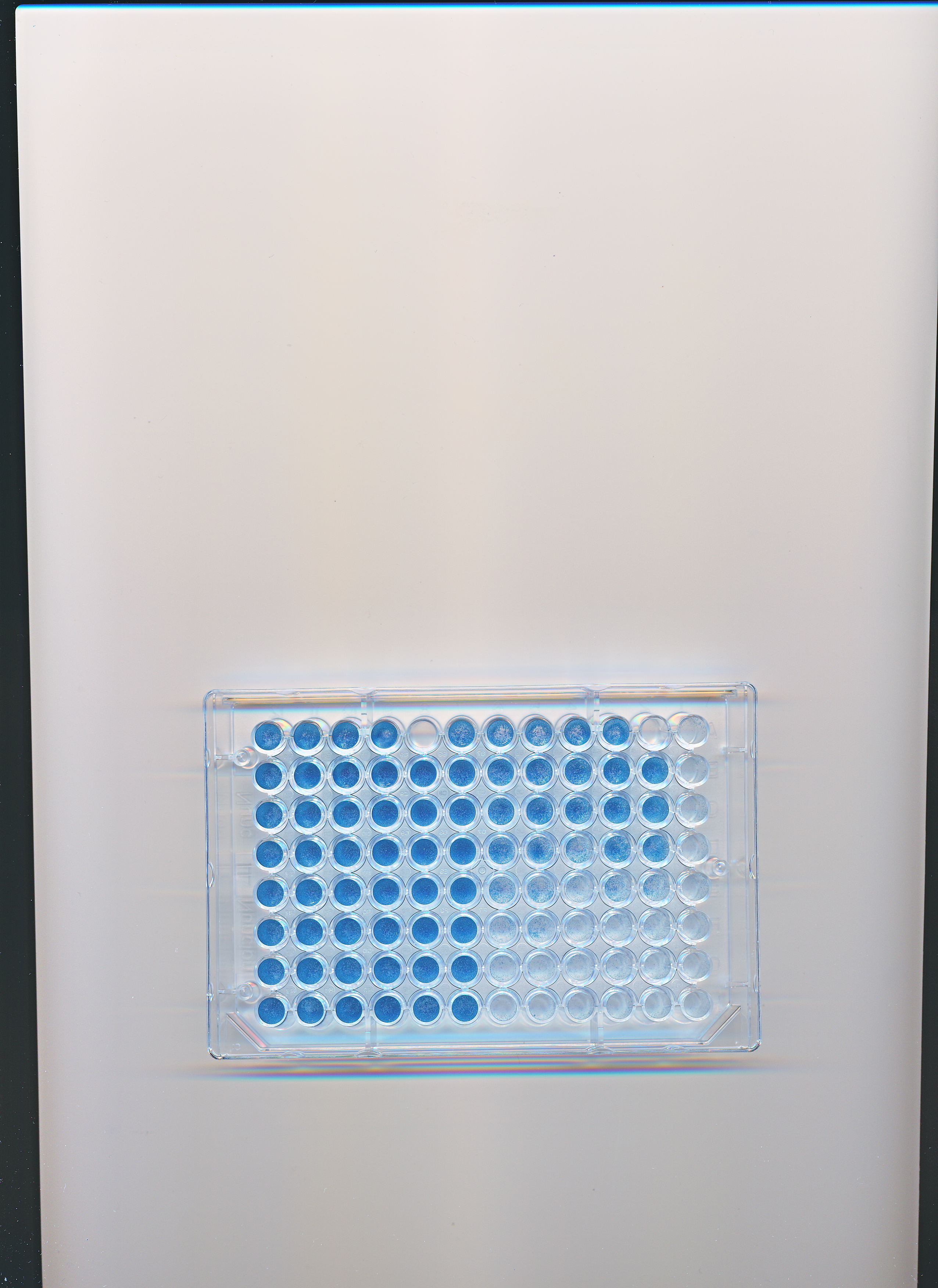

Supplement: S2 File — (ZIP) [file pone.0248960.s004.zip › Figure 3 ABC raw data/Figure 3A raw data.jpg]

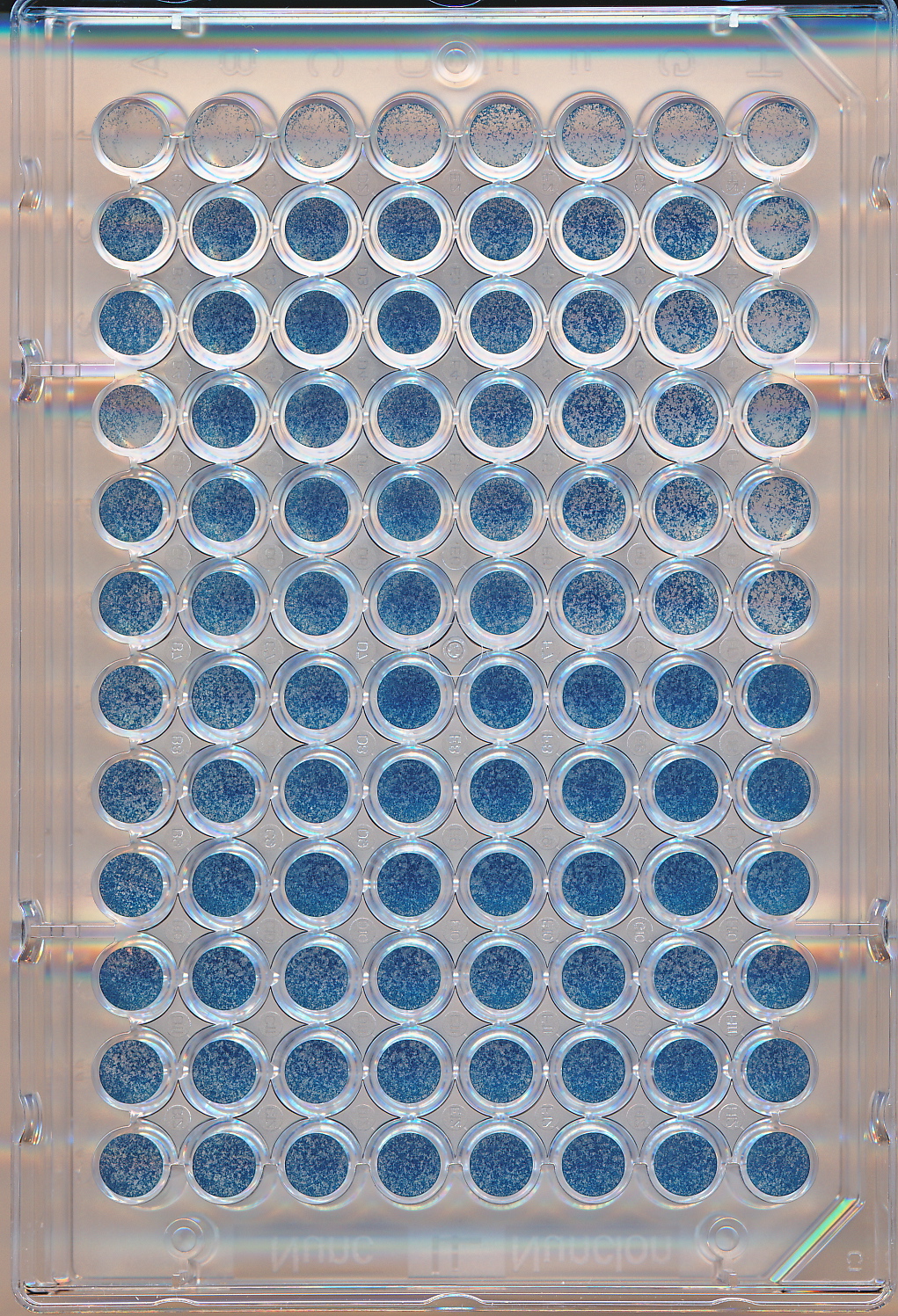

Supplement: S2 File — (ZIP) [file pone.0248960.s004.zip › Figure 3 ABC raw data/Figure 3B raw data.jpg]

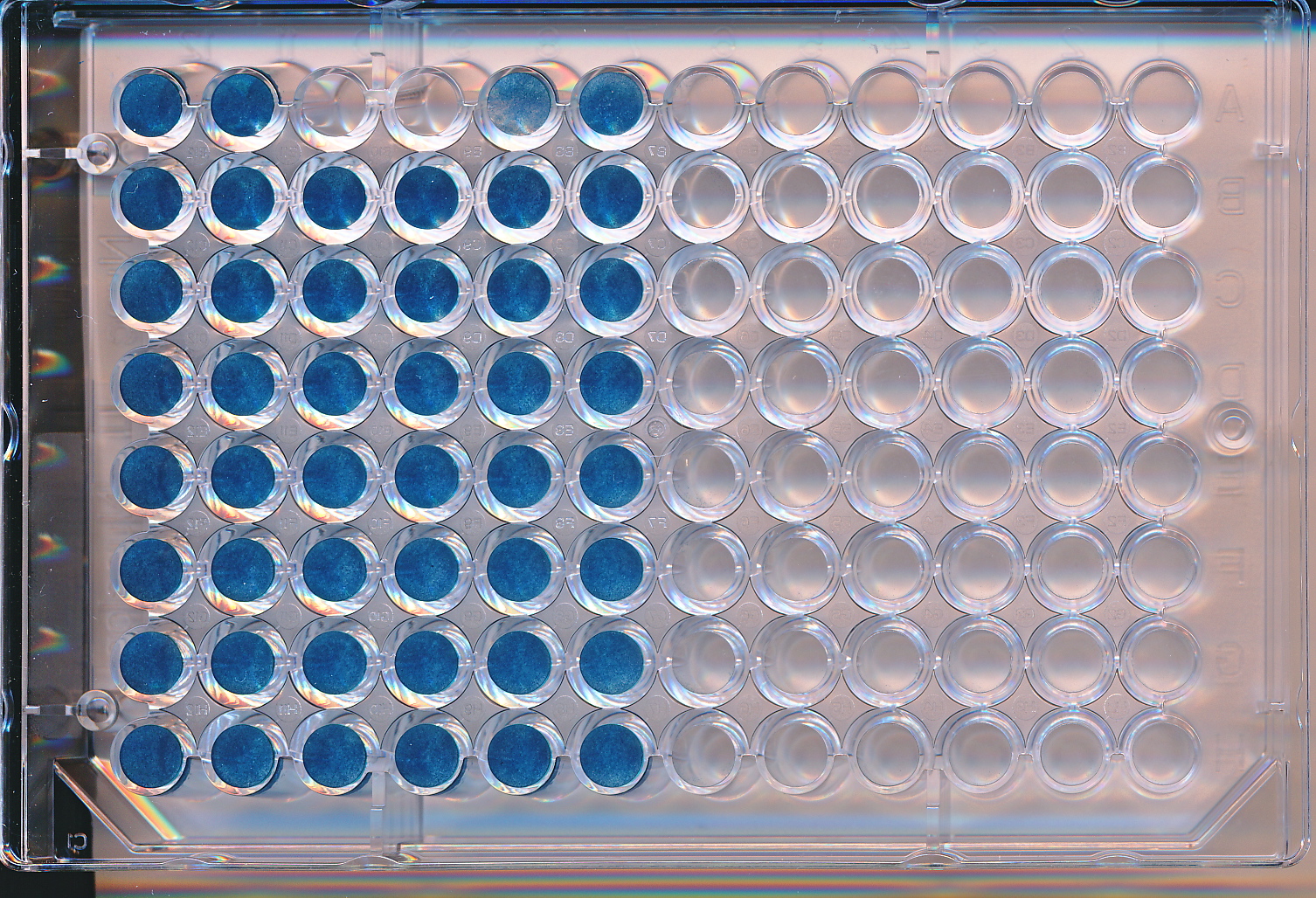

Supplement: S2 File — (ZIP) [file pone.0248960.s004.zip › Figure 3 ABC raw data/Figure 3C raw data.jpg]

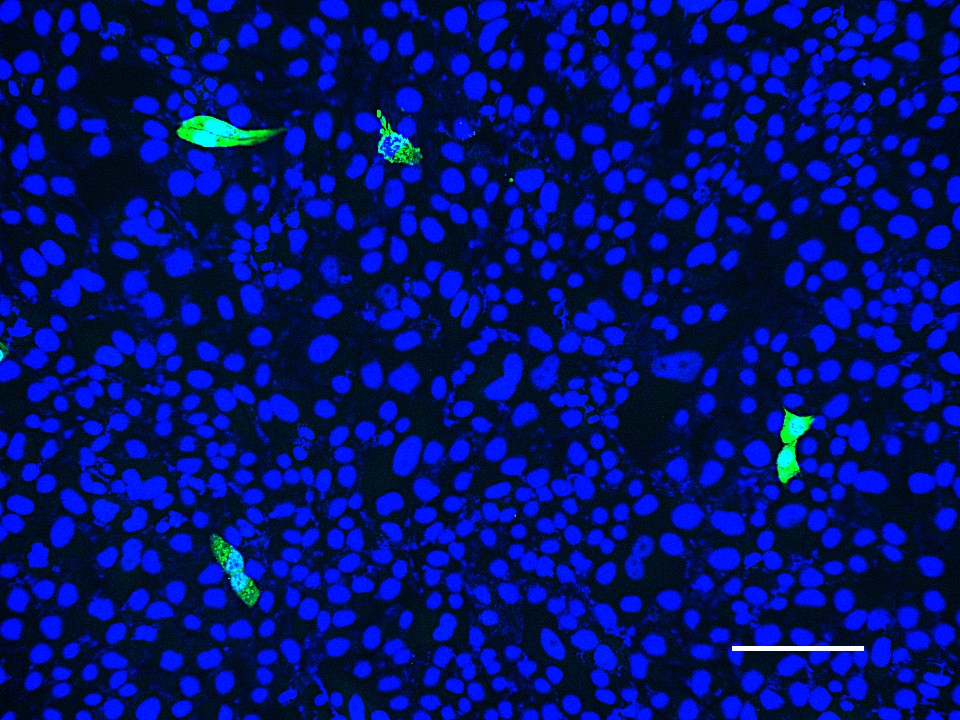

Supplement: S5 File — (ZIP) [file pone.0248960.s007.zip › Figure 4 PR8 raw data tif files/HR_200303 cycloC 12uM No1_Image_Overlay.tif]

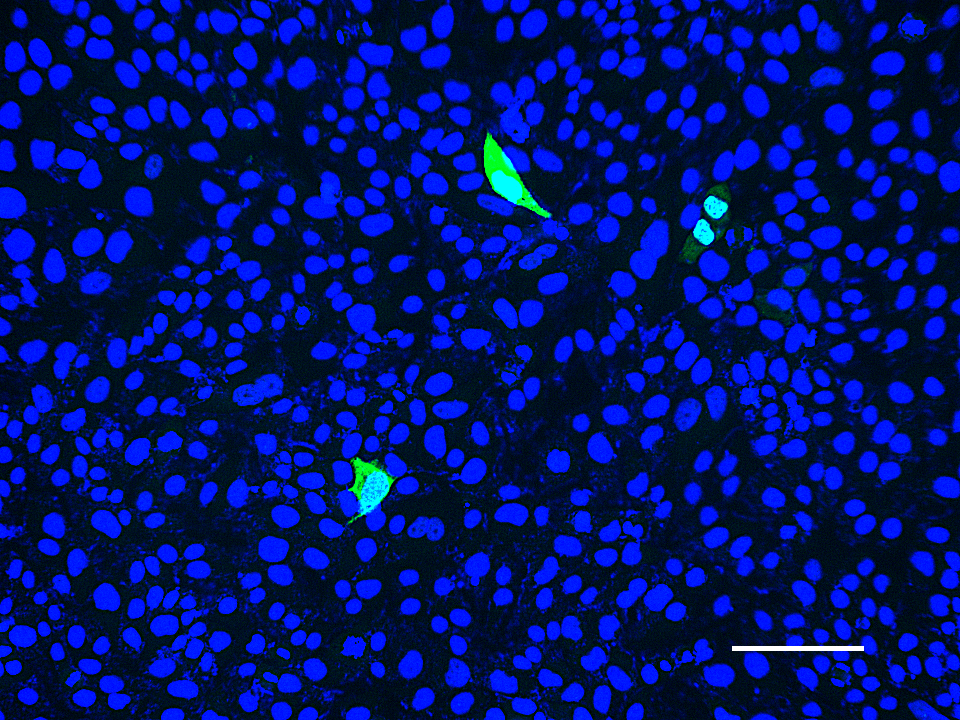

Supplement: S5 File — (ZIP) [file pone.0248960.s007.zip › Figure 4 PR8 raw data tif files/HR_200303 CycloB 12uM No2_Image_Overlay.tif]

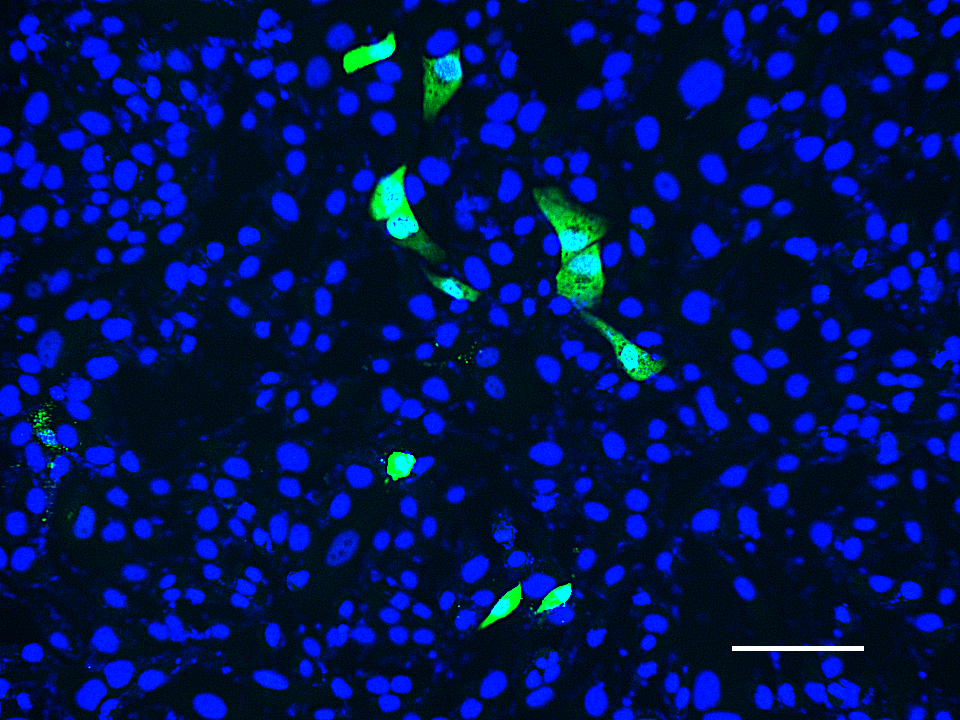

Supplement: S5 File — (ZIP) [file pone.0248960.s007.zip › Figure 4 PR8 raw data tif files/HR_200303 CycloC 6uM No2_Image_Overlay.tif]

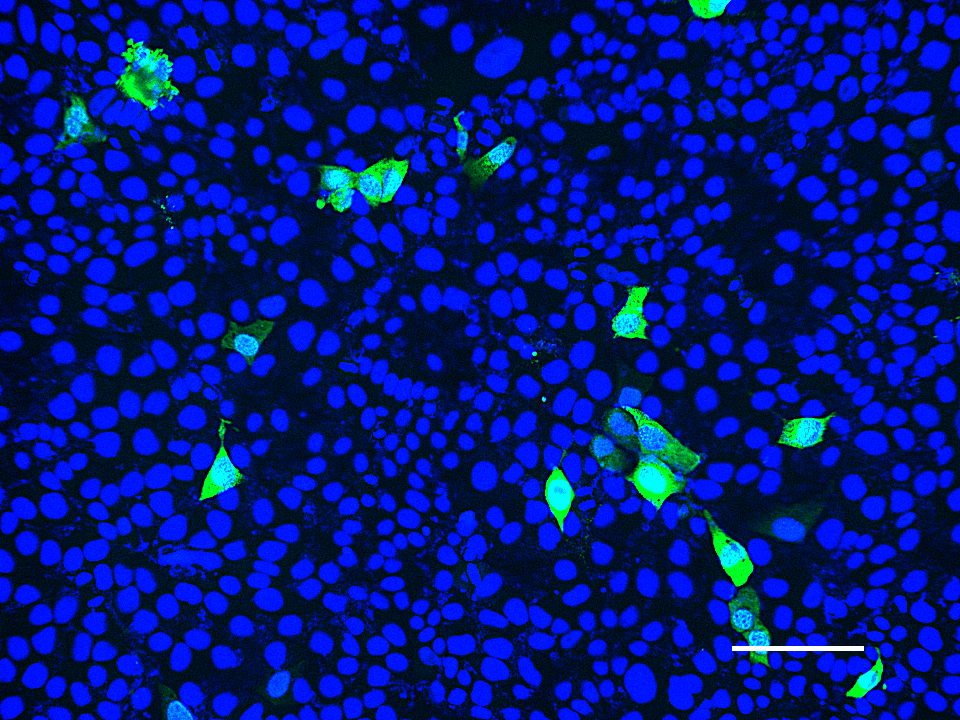

Supplement: S5 File — (ZIP) [file pone.0248960.s007.zip › Figure 4 PR8 raw data tif files/HR_200303 CycloD 6uM No2_Image_Overlay.tif]

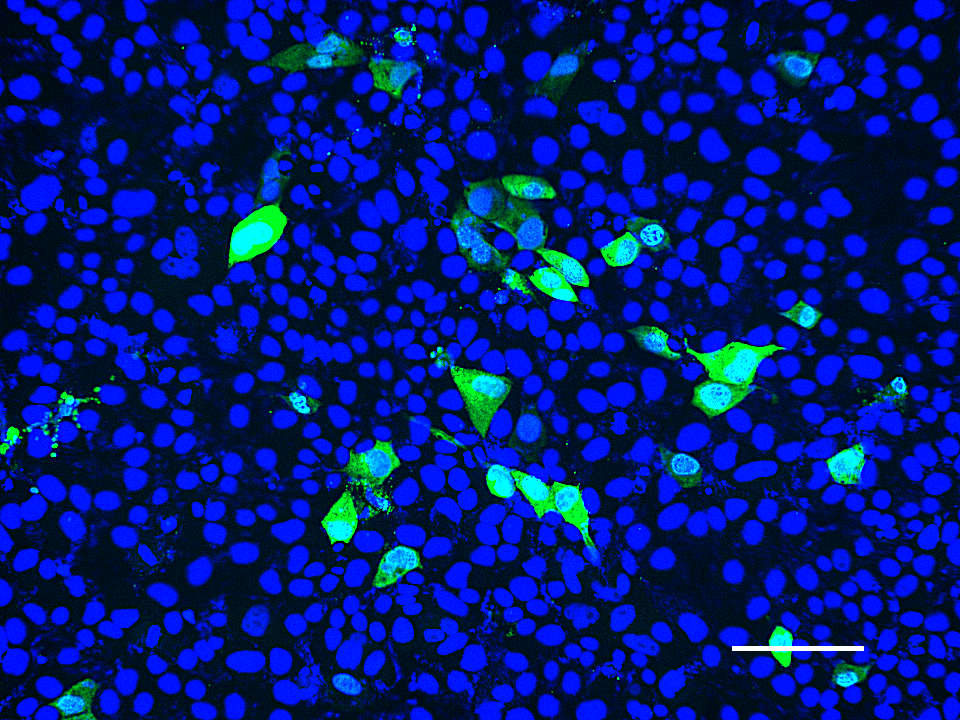

Supplement: S5 File — (ZIP) [file pone.0248960.s007.zip › Figure 4 PR8 raw data tif files/HR_200303 DMSO 00625% No2_Image_Overlay.tif]

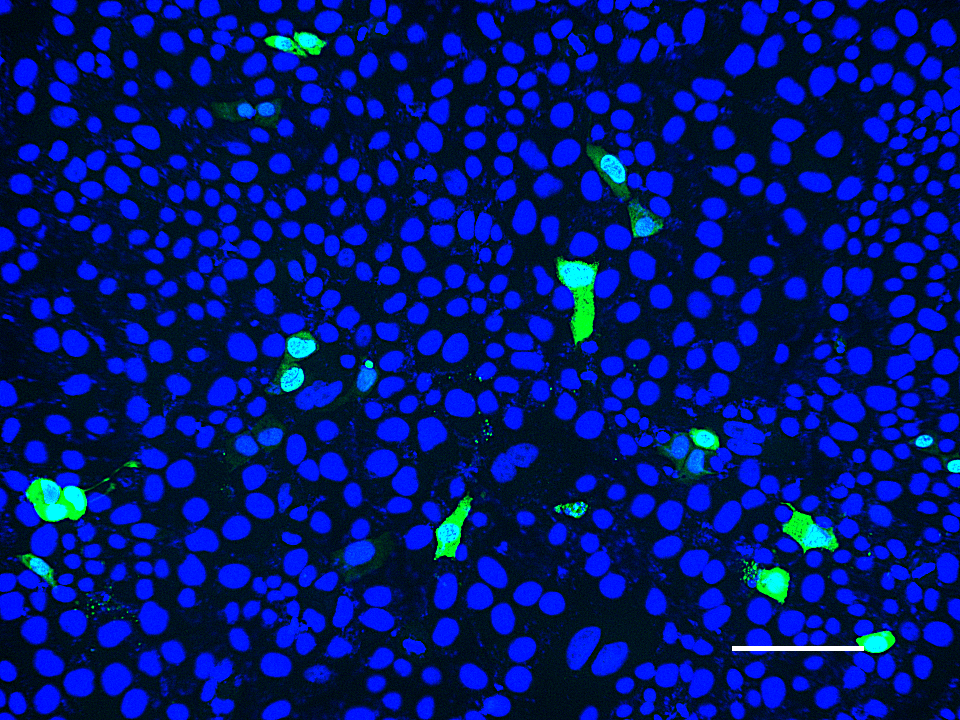

Supplement: S5 File — (ZIP) [file pone.0248960.s007.zip › Figure 4 PR8 raw data tif files/HR_200303 cycloB 3uM No3_Image_Overlay.tif]

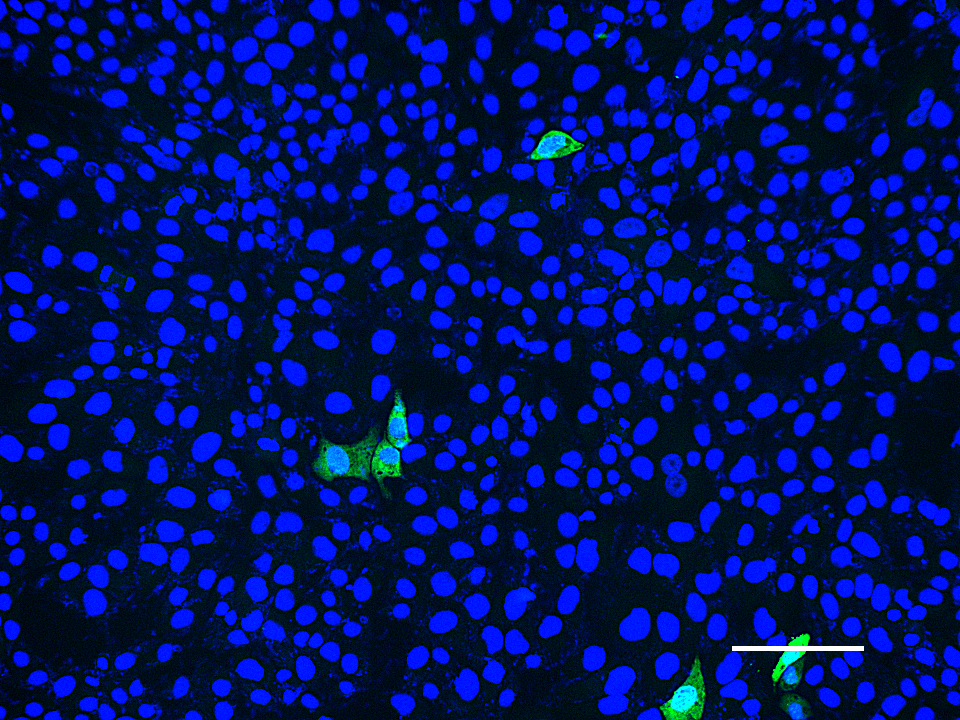

Supplement: S5 File — (ZIP) [file pone.0248960.s007.zip › Figure 4 PR8 raw data tif files/HR_200303 CycloD 12uM No2_Image_Overlay.tif]

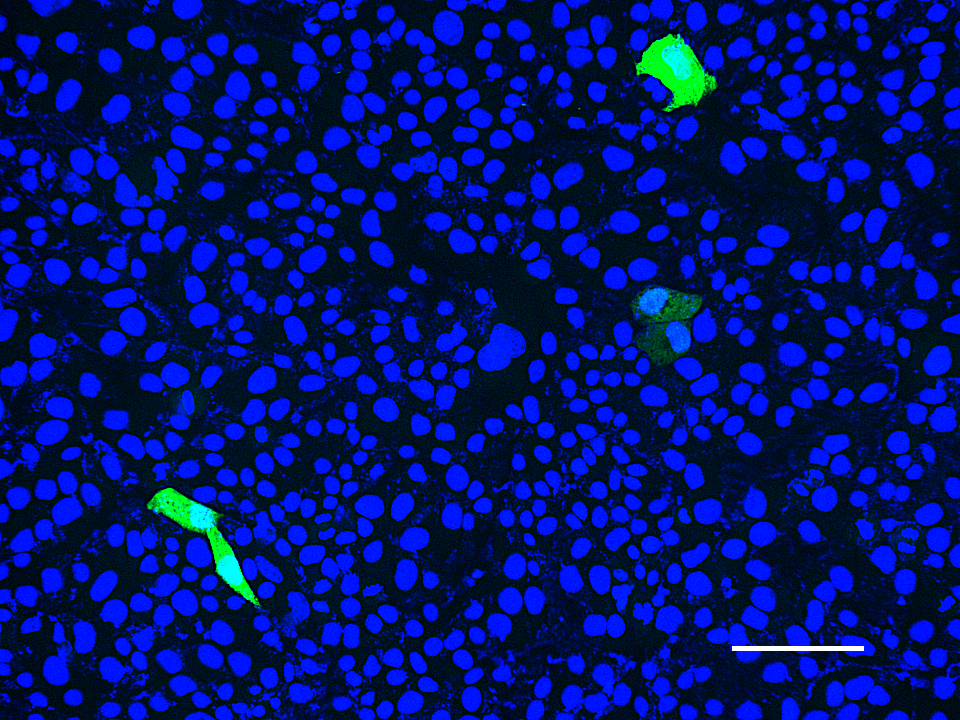

Supplement: S5 File — (ZIP) [file pone.0248960.s007.zip › Figure 4 PR8 raw data tif files/HR_200303 S 6uM No3_Image_Overlay.tif]

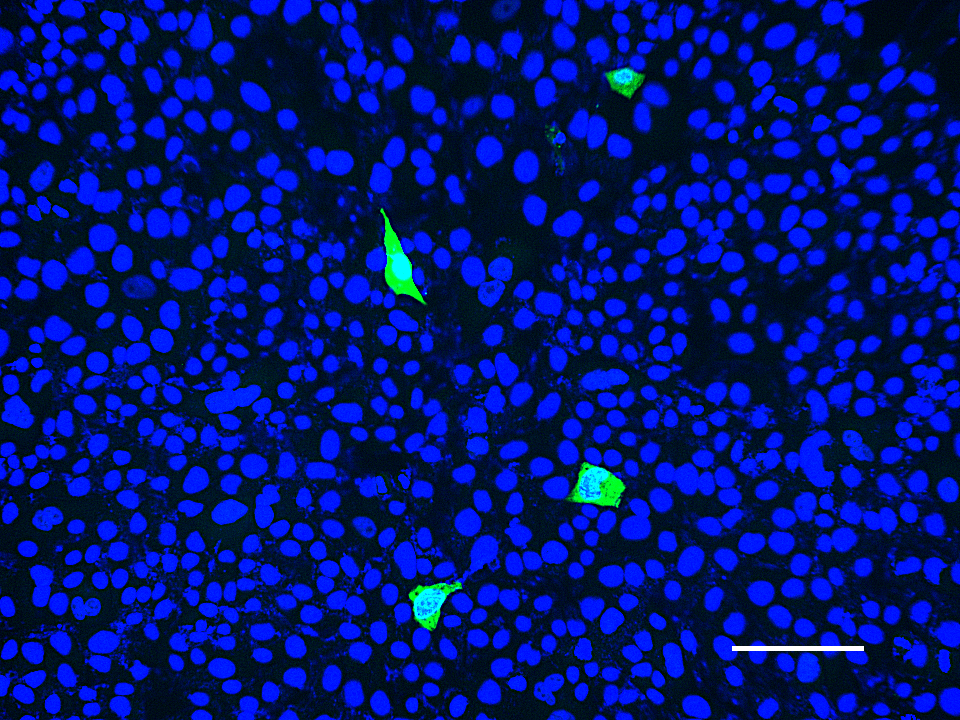

Supplement: S5 File — (ZIP) [file pone.0248960.s007.zip › Figure 4 PR8 raw data tif files/HR_200303 CycloA 6uM No2_Image_Overlay.tif]

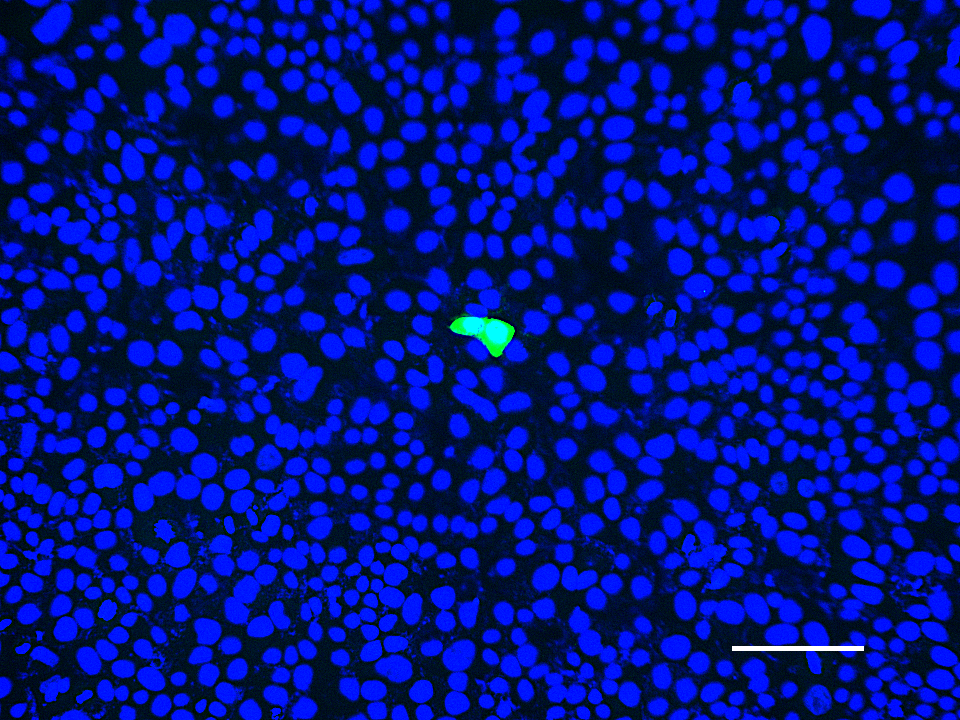

Supplement: S5 File — (ZIP) [file pone.0248960.s007.zip › Figure 4 PR8 raw data tif files/HR_200303 S 12uM No2_Image_Overlay.tif]

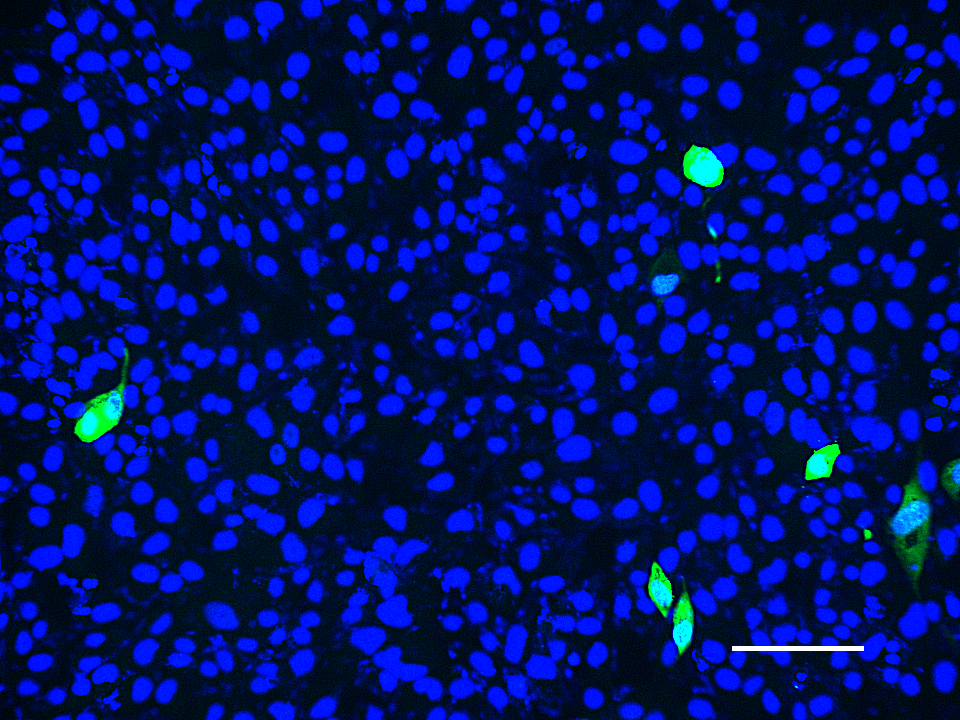

Supplement: S5 File — (ZIP) [file pone.0248960.s007.zip › Figure 4 PR8 raw data tif files/HR_200303 S 3uM No1_Image_Overlay.tif]

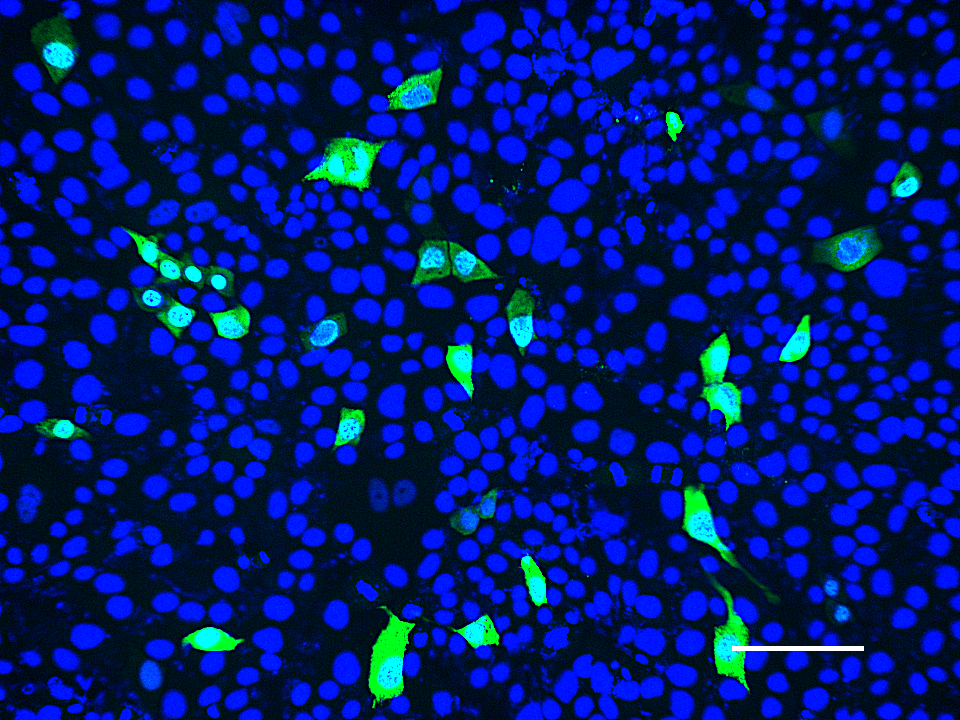

Supplement: S5 File — (ZIP) [file pone.0248960.s007.zip › Figure 4 PR8 raw data tif files/HR_200303 cycloD 3uM No3_Image_Overlay.tif]

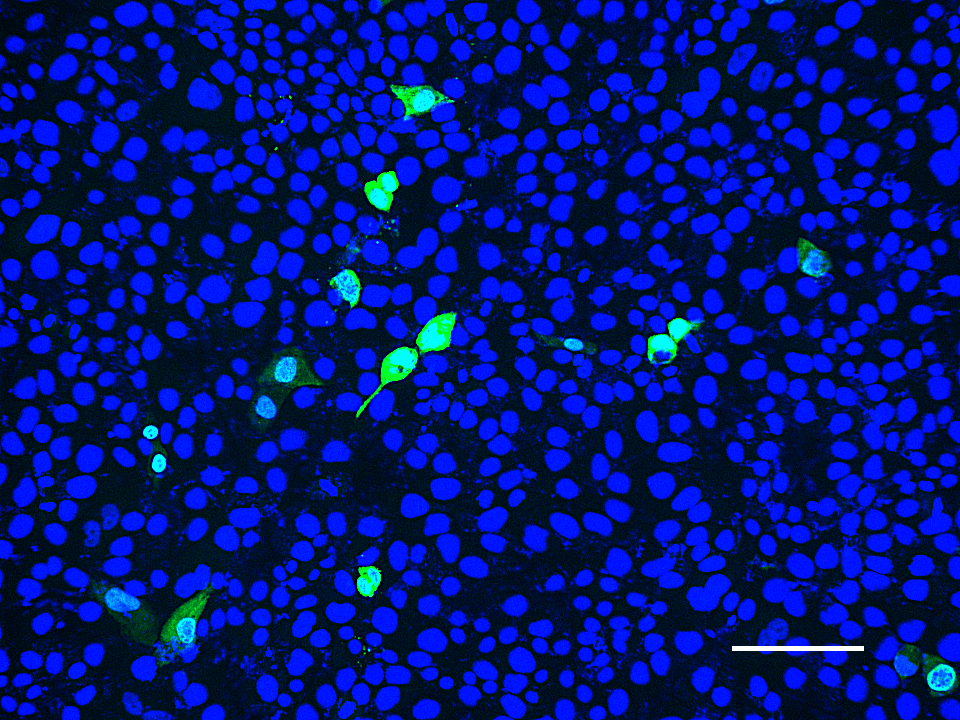

Supplement: S5 File — (ZIP) [file pone.0248960.s007.zip › Figure 4 PR8 raw data tif files/HR_200303 CycloC 3uM No3_Image_Overlay.tif]

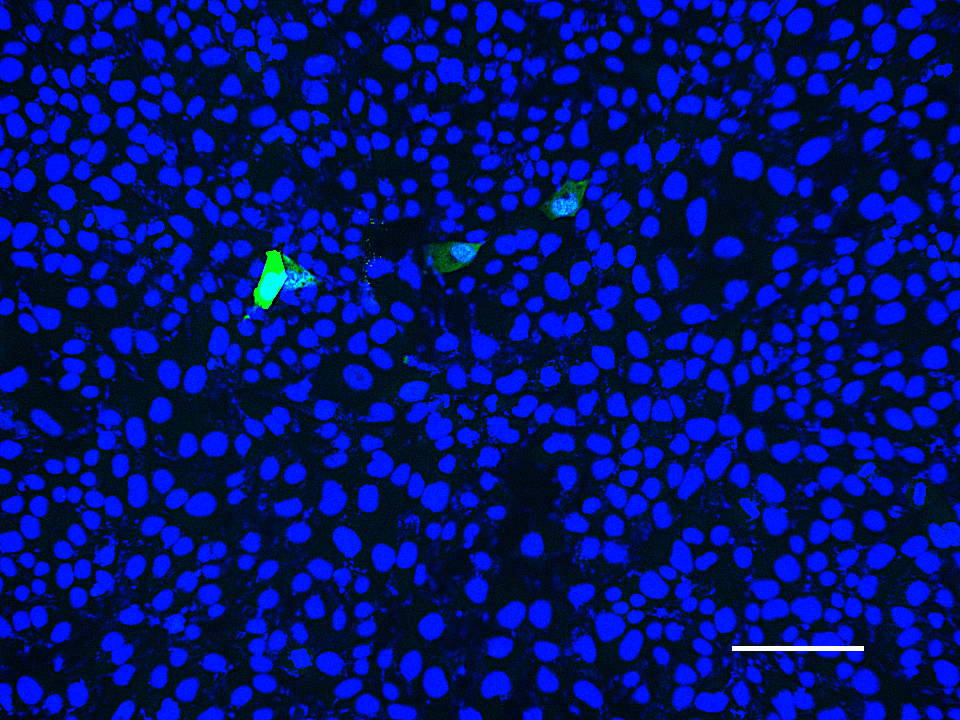

Supplement: S5 File — (ZIP) [file pone.0248960.s007.zip › Figure 4 PR8 raw data tif files/HR_200303 cycloA 12uM No1_Image_Overlay.tif]

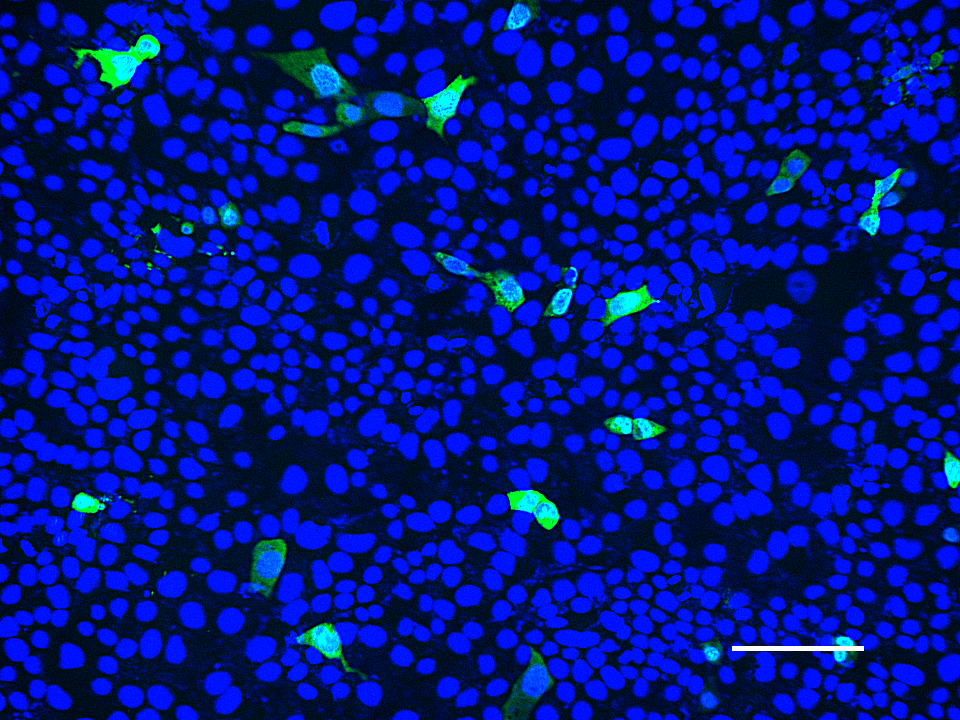

Supplement: S5 File — (ZIP) [file pone.0248960.s007.zip › Figure 4 PR8 raw data tif files/HR_200303 S 3uM No3_Image_Overlay.tif]

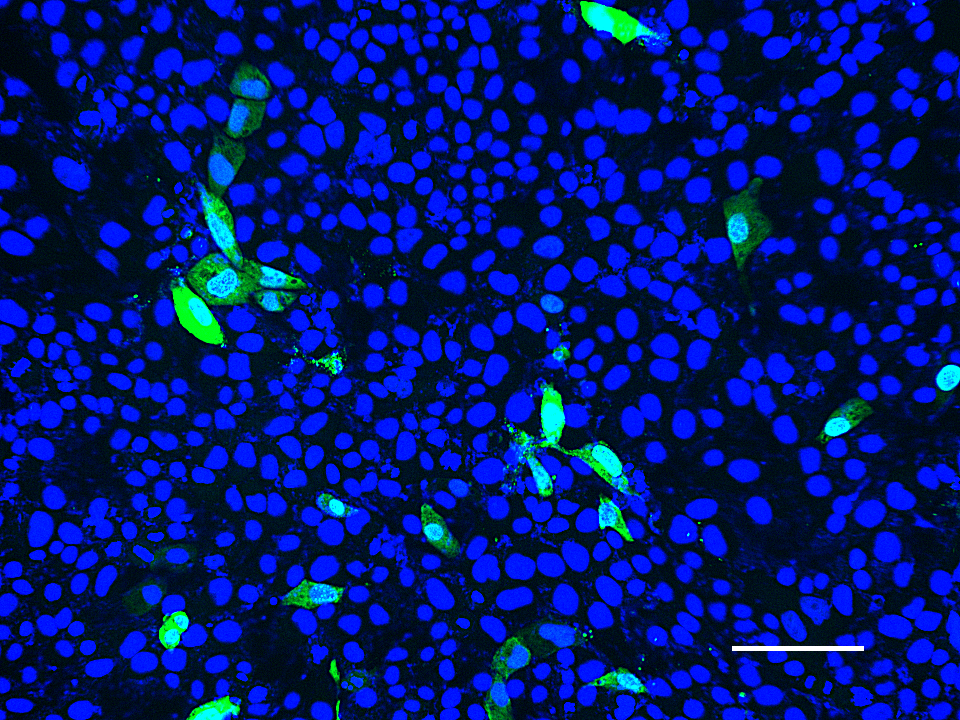

Supplement: S5 File — (ZIP) [file pone.0248960.s007.zip › Figure 4 PR8 raw data tif files/HR_200303 DMSO 0125% No2_Image_Overlay.tif]

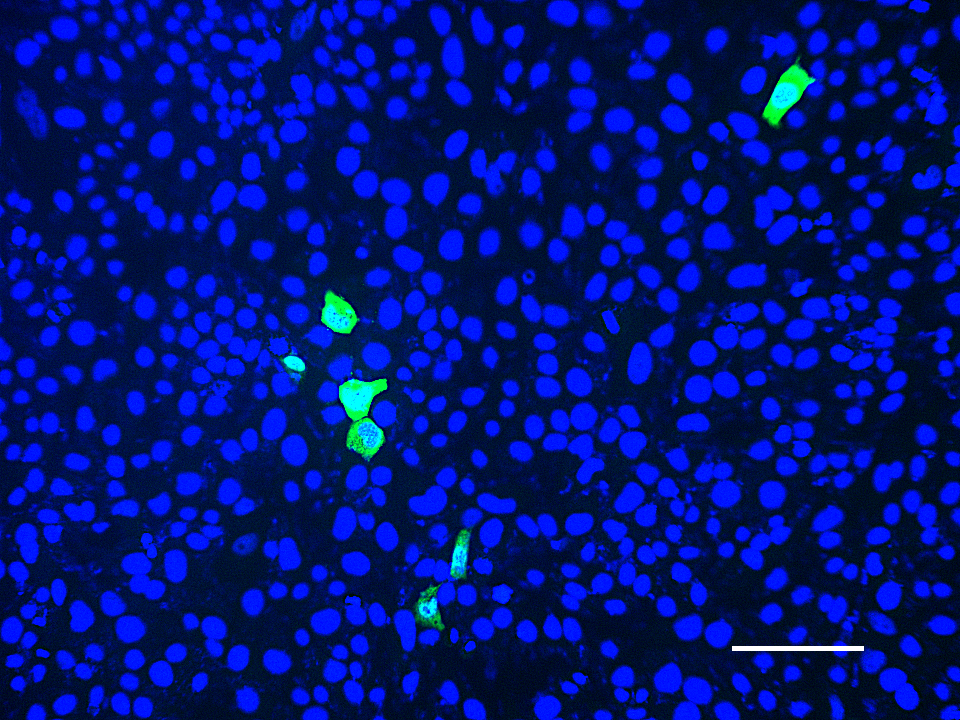

Supplement: S5 File — (ZIP) [file pone.0248960.s007.zip › Figure 4 PR8 raw data tif files/HR_200303 CycloB 6uM No2_Image_Overlay.tif]

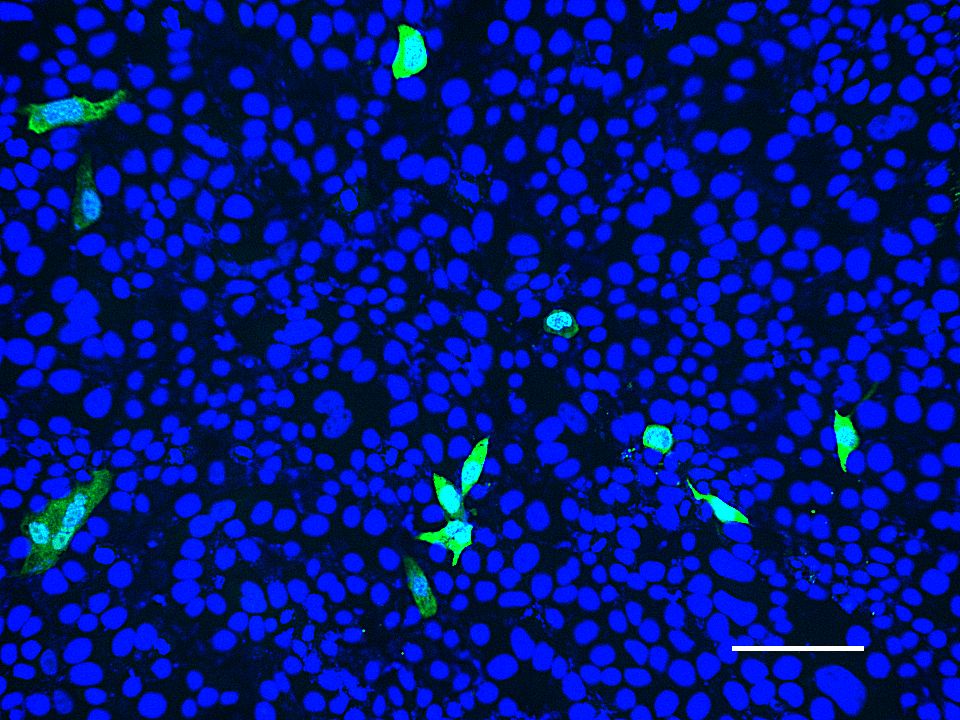

Supplement: S5 File — (ZIP) [file pone.0248960.s007.zip › Figure 4 PR8 raw data tif files/HR_200303 cycloA 3uM No3_Image_Overlay.tif]

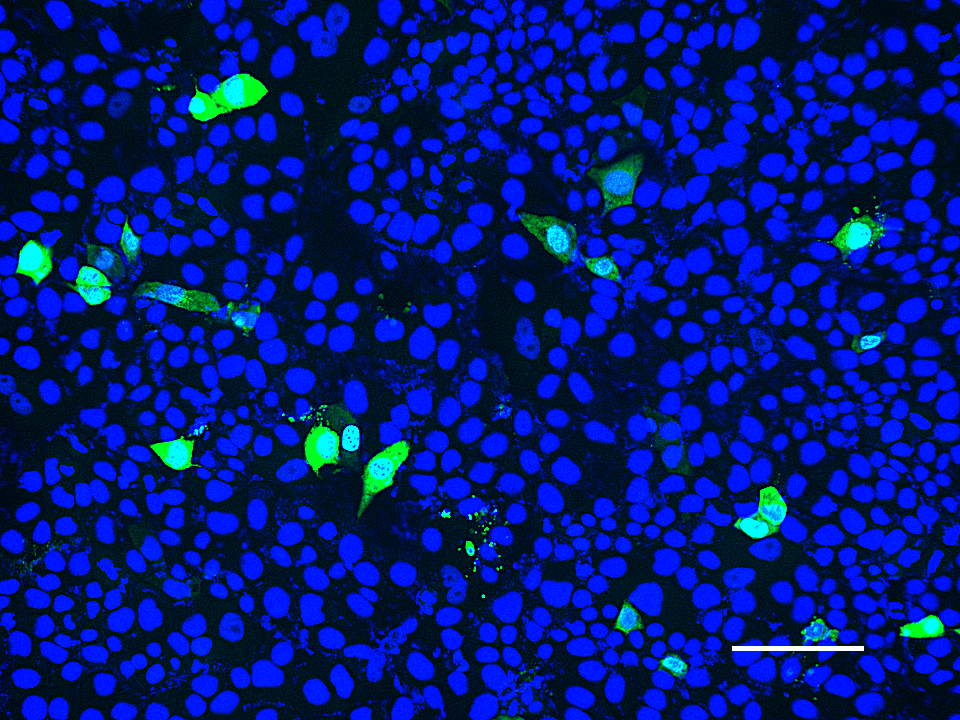

Supplement: S5 File — (ZIP) [file pone.0248960.s007.zip › Figure 4 PR8 raw data tif files/HR_200303 DMSO 003125% No2_Image_Overlay.tif]

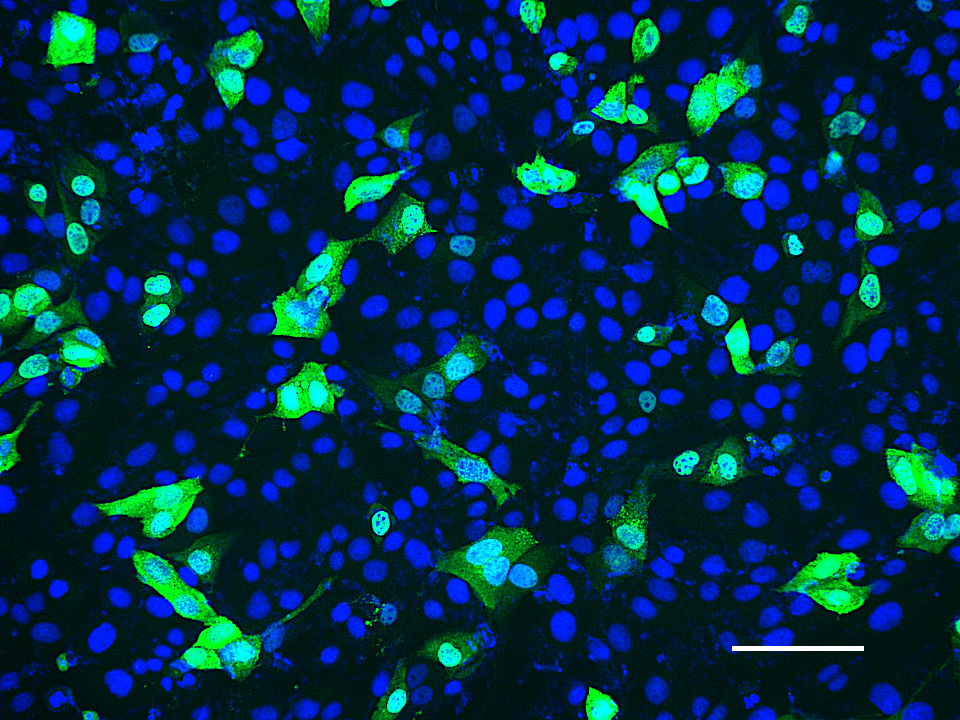

Supplement: S6 File — (ZIP) [file pone.0248960.s008.zip › Figure 4 WSN raw data tif files/2_bakuB 625uM_20.tif]

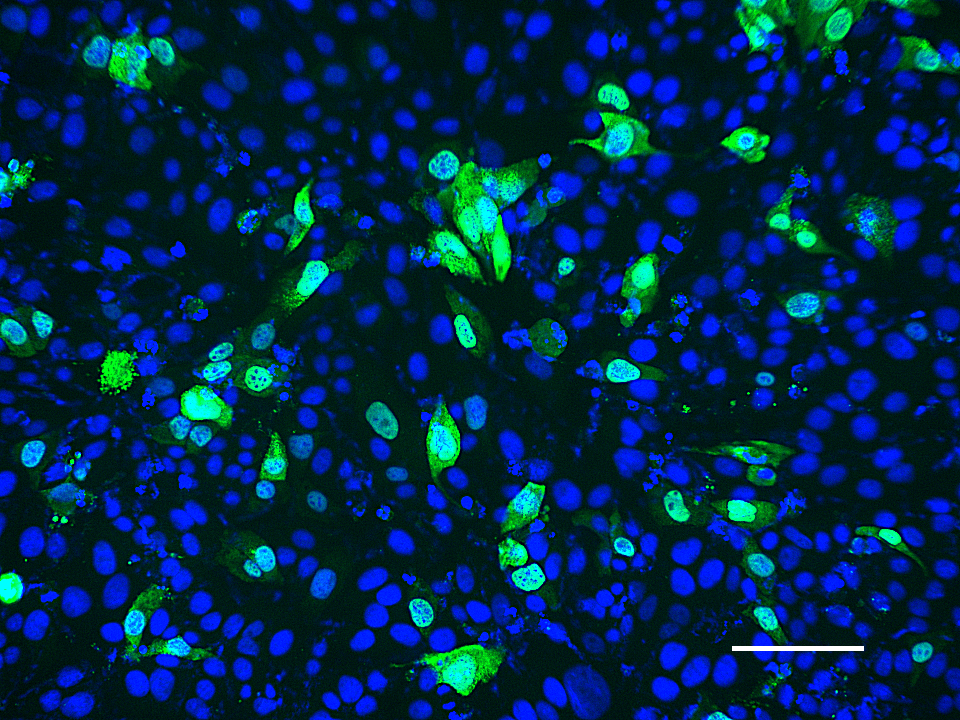

Supplement: S6 File — (ZIP) [file pone.0248960.s008.zip › Figure 4 WSN raw data tif files/3_bakuC 125uM_20.tif]

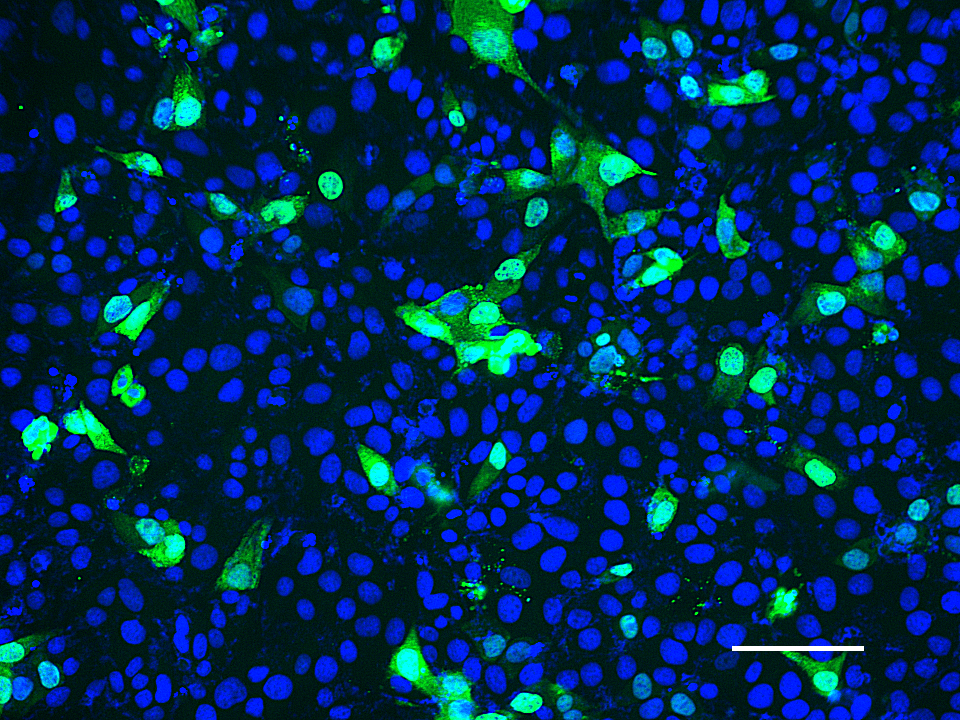

Supplement: S6 File — (ZIP) [file pone.0248960.s008.zip › Figure 4 WSN raw data tif files/3_bakuD 625uM_20.tif]

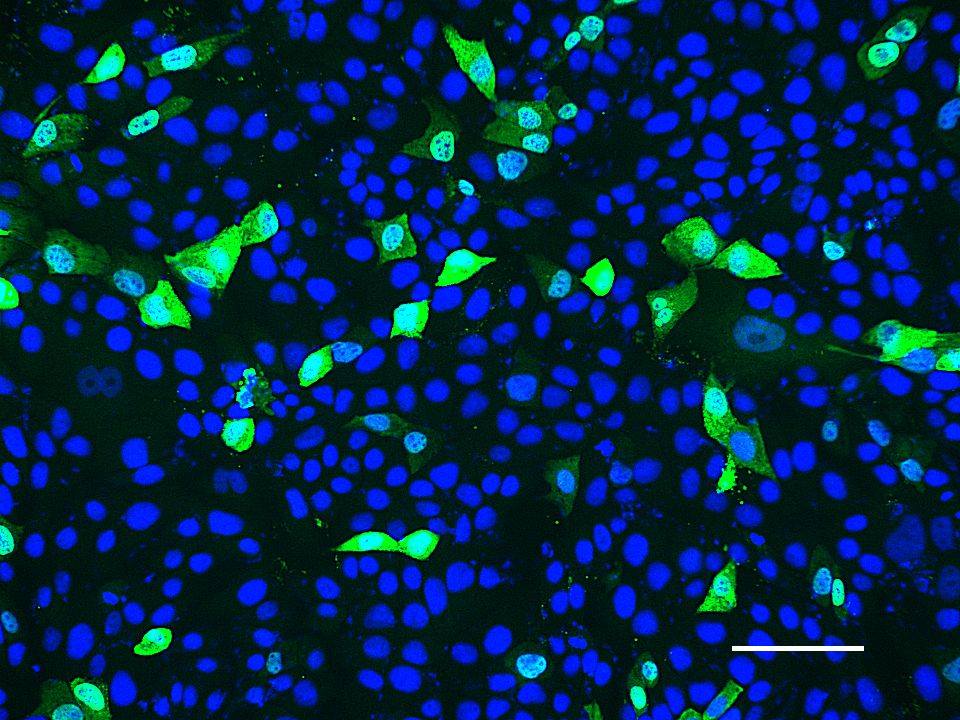

Supplement: S6 File — (ZIP) [file pone.0248960.s008.zip › Figure 4 WSN raw data tif files/CycloBakuA 125uM_1 _20_51.tif]

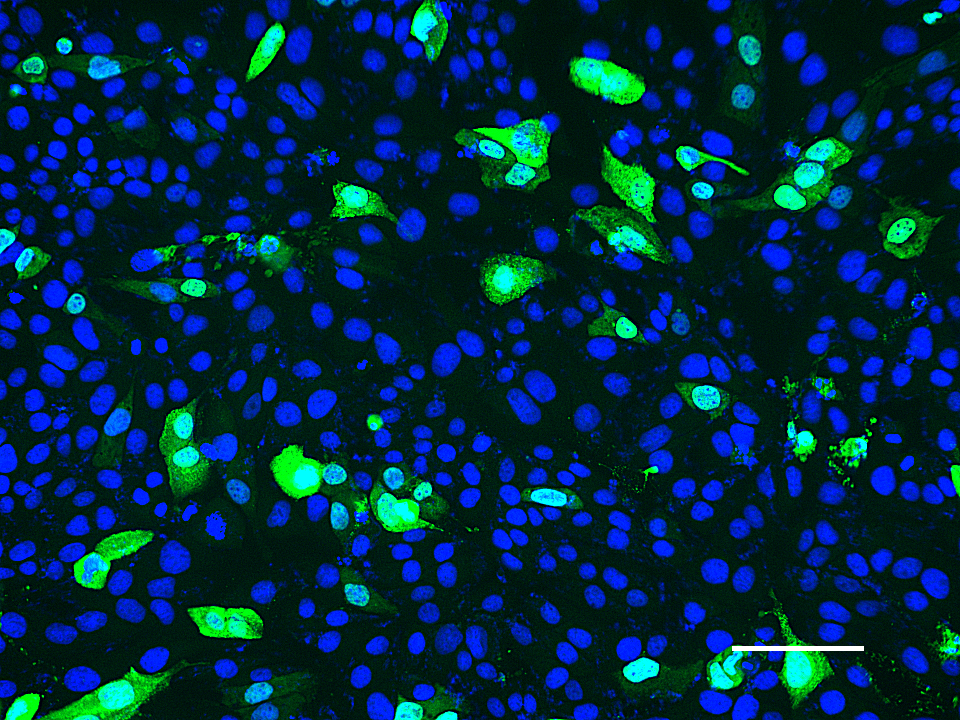

Supplement: S6 File — (ZIP) [file pone.0248960.s008.zip › Figure 4 WSN raw data tif files/CycloBakuA 3125uM 3_20_04.tif]

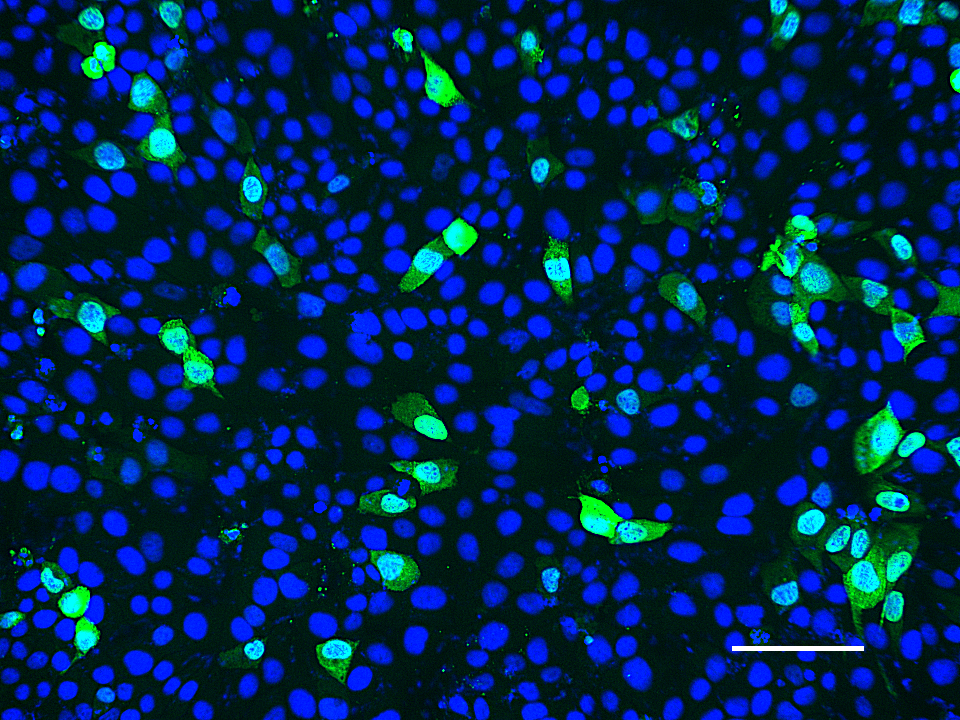

Supplement: S6 File — (ZIP) [file pone.0248960.s008.zip › Figure 4 WSN raw data tif files/CycloBakuA 625uM 3_20_02.tif]

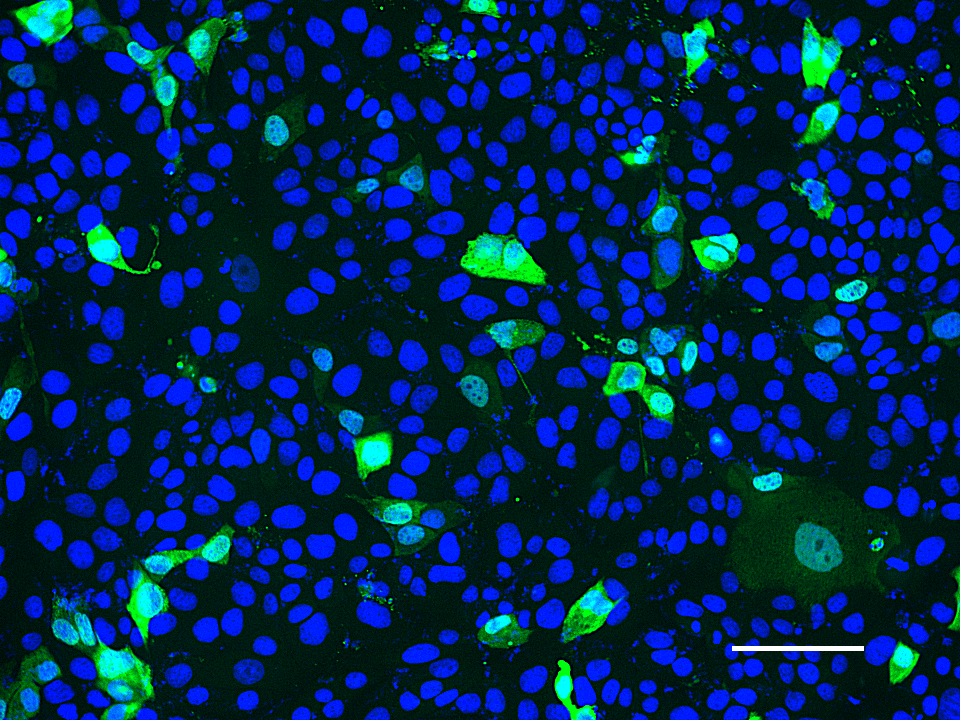

Supplement: S6 File — (ZIP) [file pone.0248960.s008.zip › Figure 4 WSN raw data tif files/CycloBakuB 125uM 1 _20_08.tif]

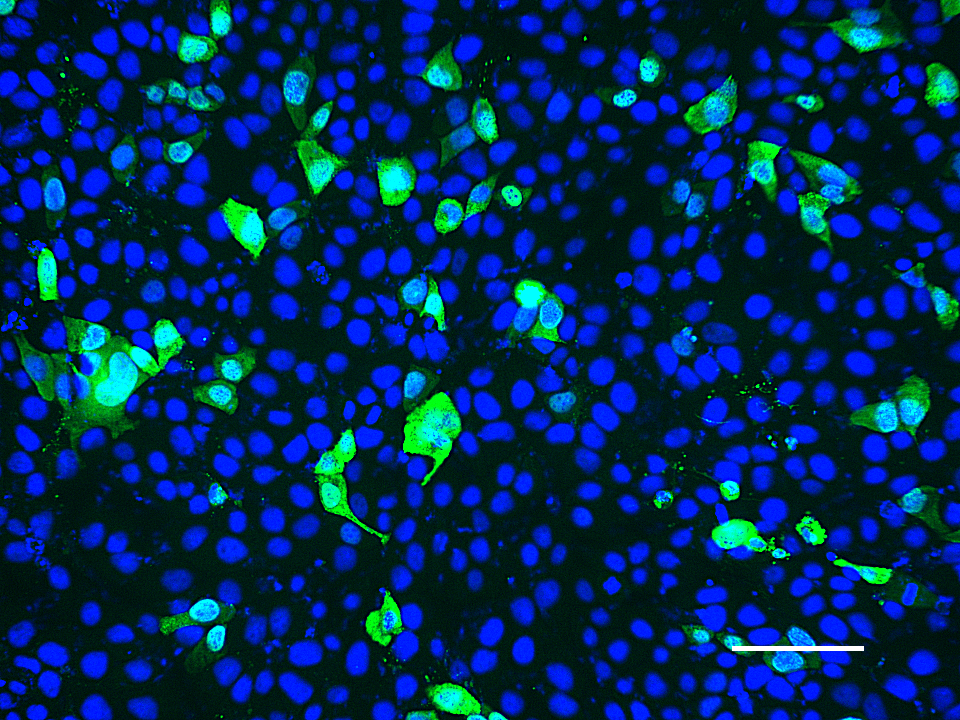

Supplement: S6 File — (ZIP) [file pone.0248960.s008.zip › Figure 4 WSN raw data tif files/CycloBakuB 3125uM 1_20_12.tif]

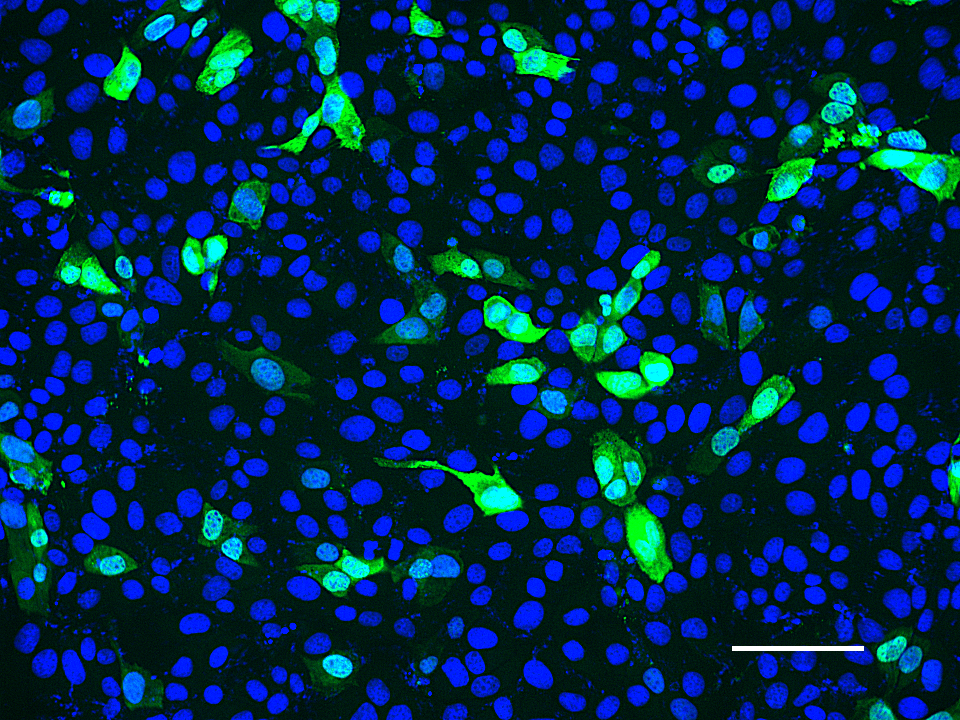

Supplement: S6 File — (ZIP) [file pone.0248960.s008.zip › Figure 4 WSN raw data tif files/CycloBakuC 125uM 1_20_32.tif]

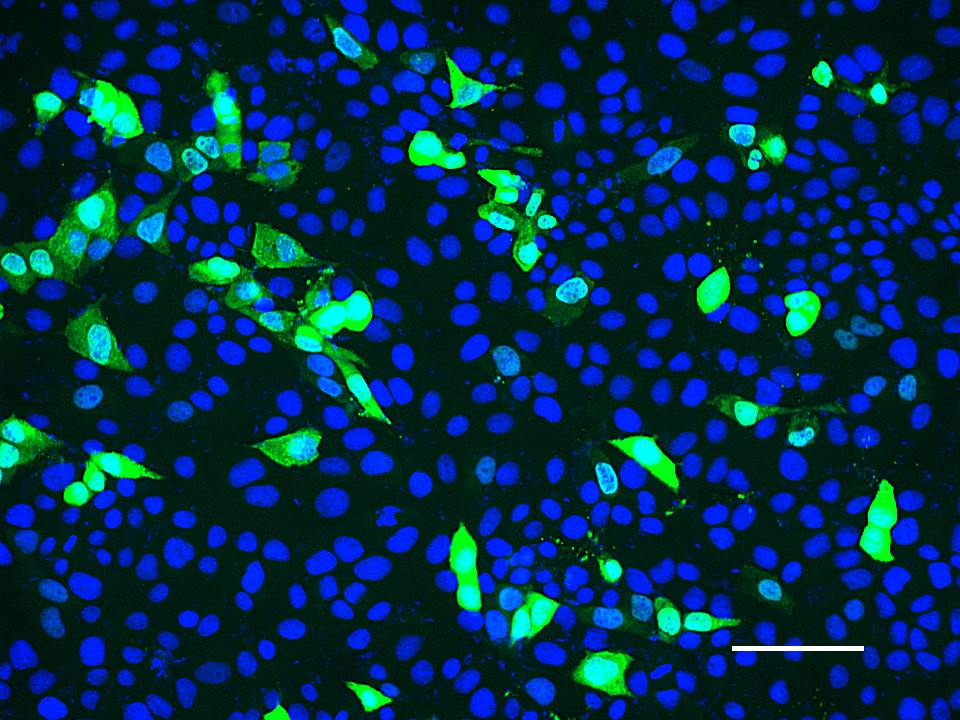

Supplement: S6 File — (ZIP) [file pone.0248960.s008.zip › Figure 4 WSN raw data tif files/CycloBakuC 3125uM 2_20_44.tif]

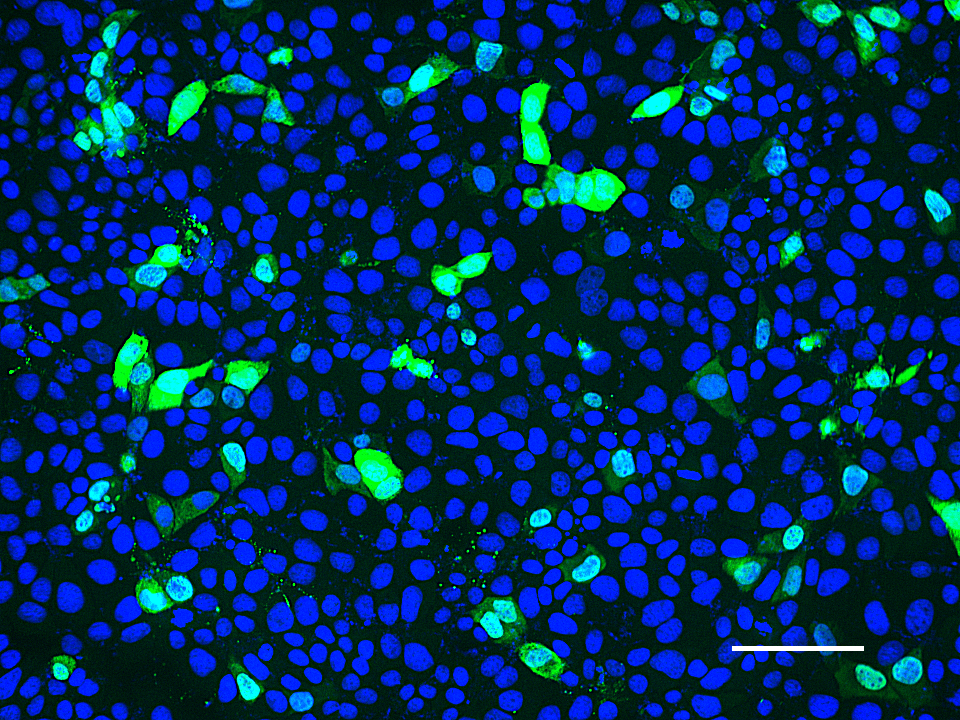

Supplement: S6 File — (ZIP) [file pone.0248960.s008.zip › Figure 4 WSN raw data tif files/CycloBakuC 625uM 2_20_42.tif]

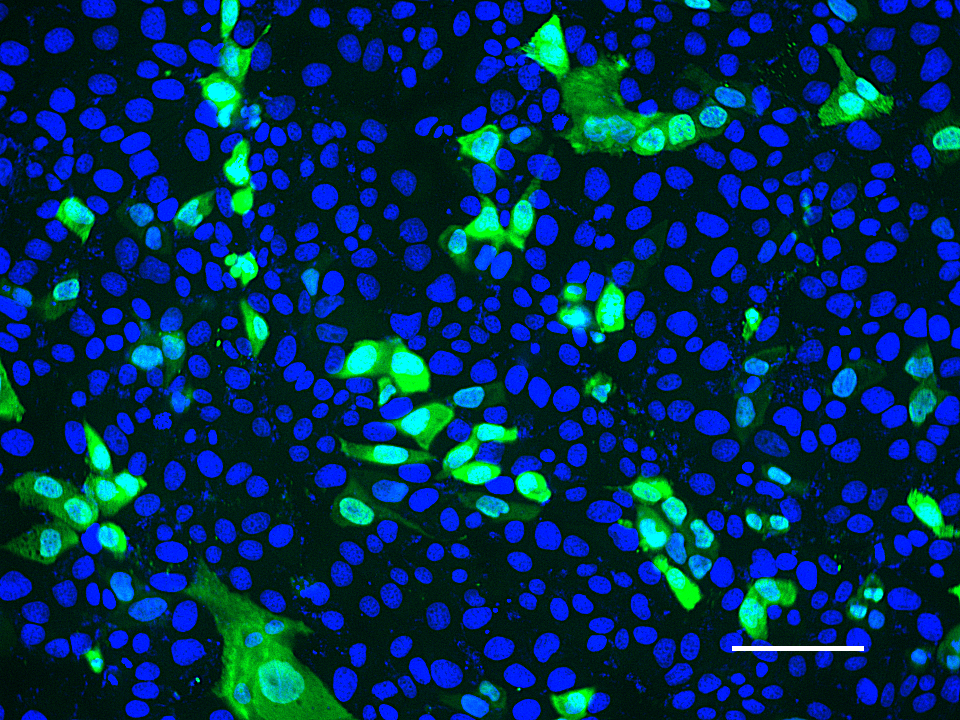

Supplement: S6 File — (ZIP) [file pone.0248960.s008.zip › Figure 4 WSN raw data tif files/CycloBakuD 125uM 1_20_56.tif]

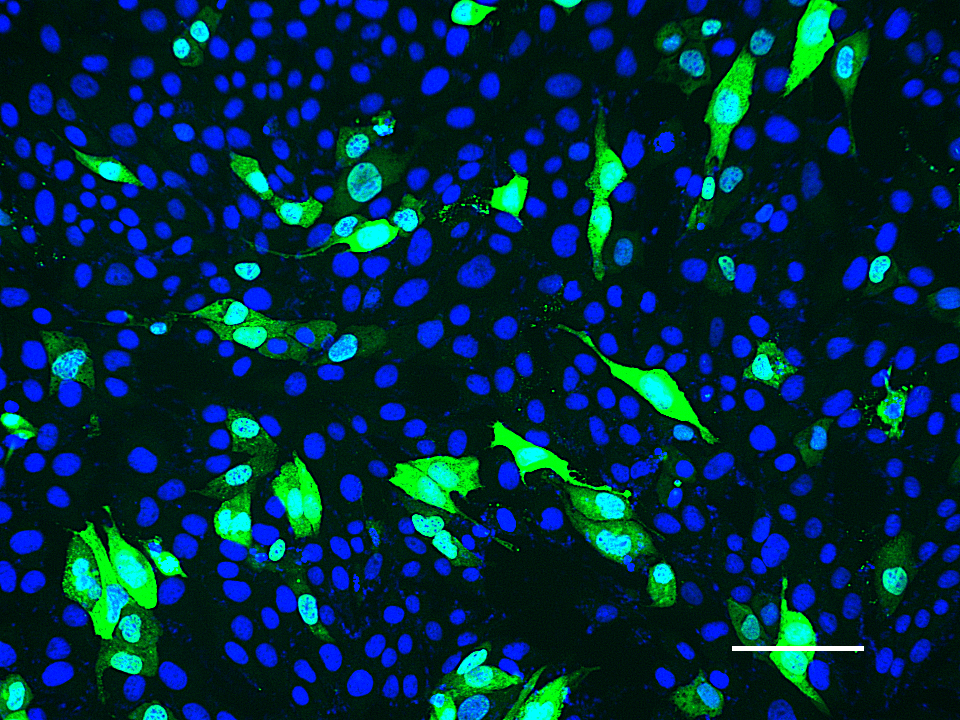

Supplement: S6 File — (ZIP) [file pone.0248960.s008.zip › Figure 4 WSN raw data tif files/CycloBakuD 3125uM 3_20_76.tif]

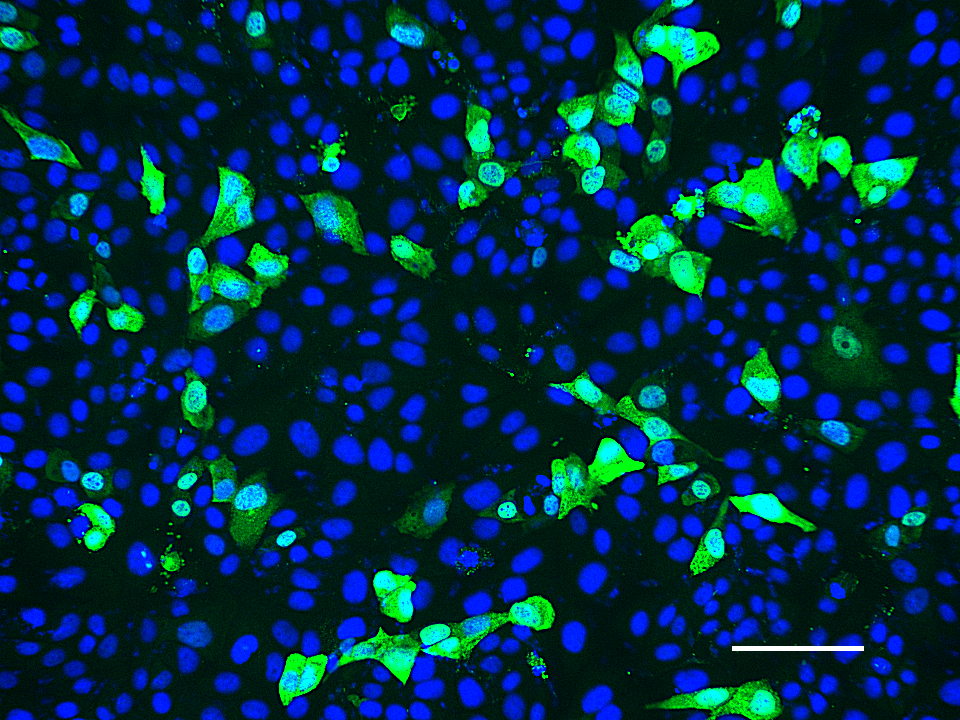

Supplement: S6 File — (ZIP) [file pone.0248960.s008.zip › Figure 4 WSN raw data tif files/DMSO 003125% 3_20_23.tif]

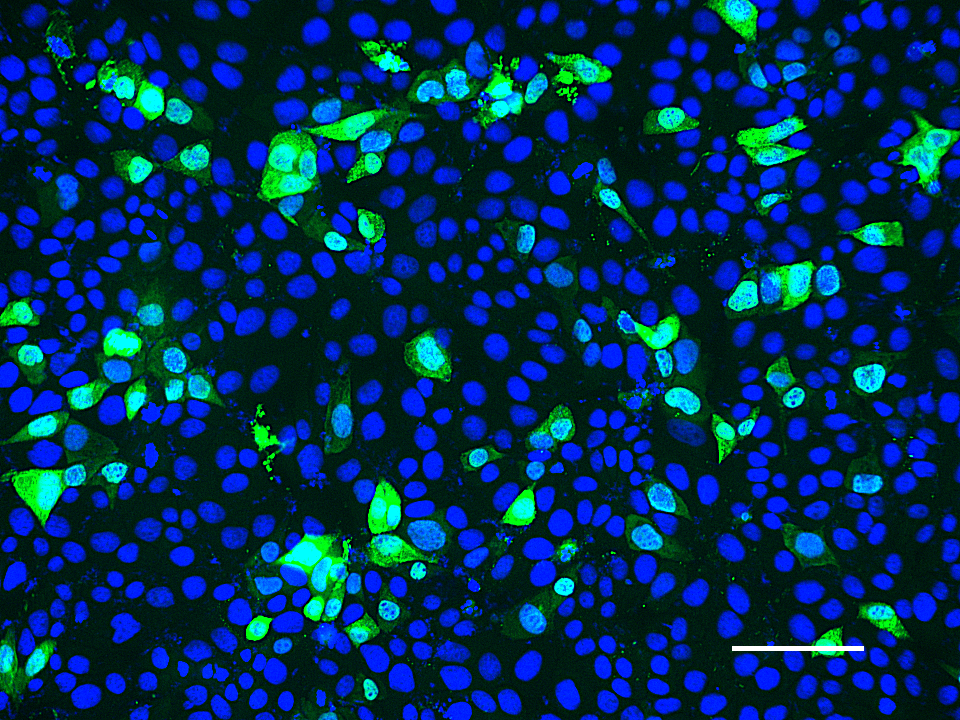

Supplement: S6 File — (ZIP) [file pone.0248960.s008.zip › Figure 4 WSN raw data tif files/DMSO 00625% 3_20_21.tif]

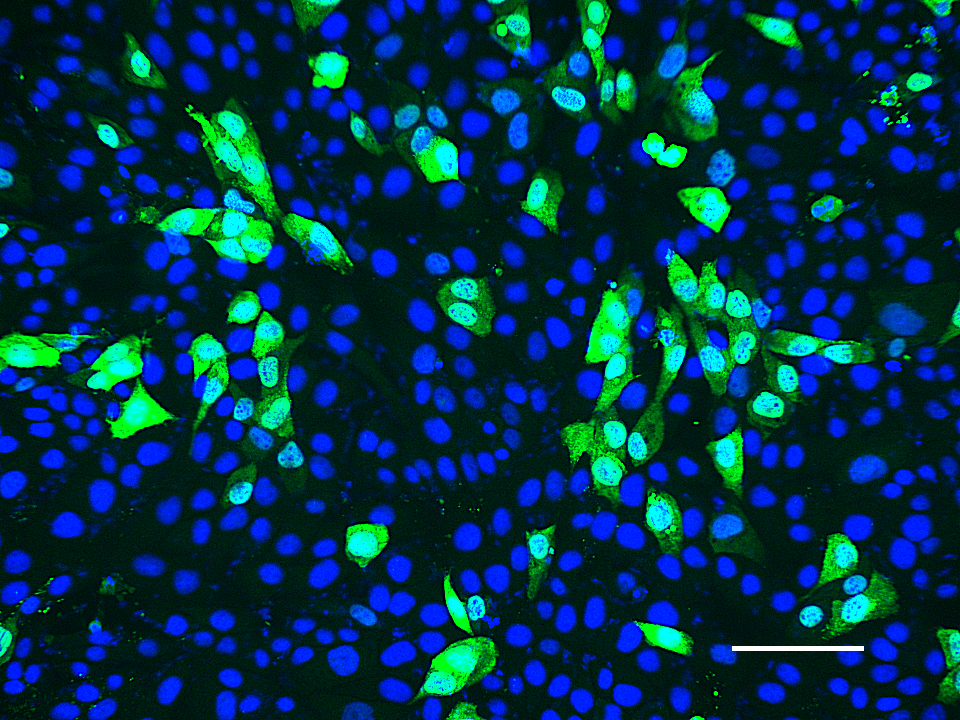

Supplement: S6 File — (ZIP) [file pone.0248960.s008.zip › Figure 4 WSN raw data tif files/DMSO 0125% 3_20_19.tif]

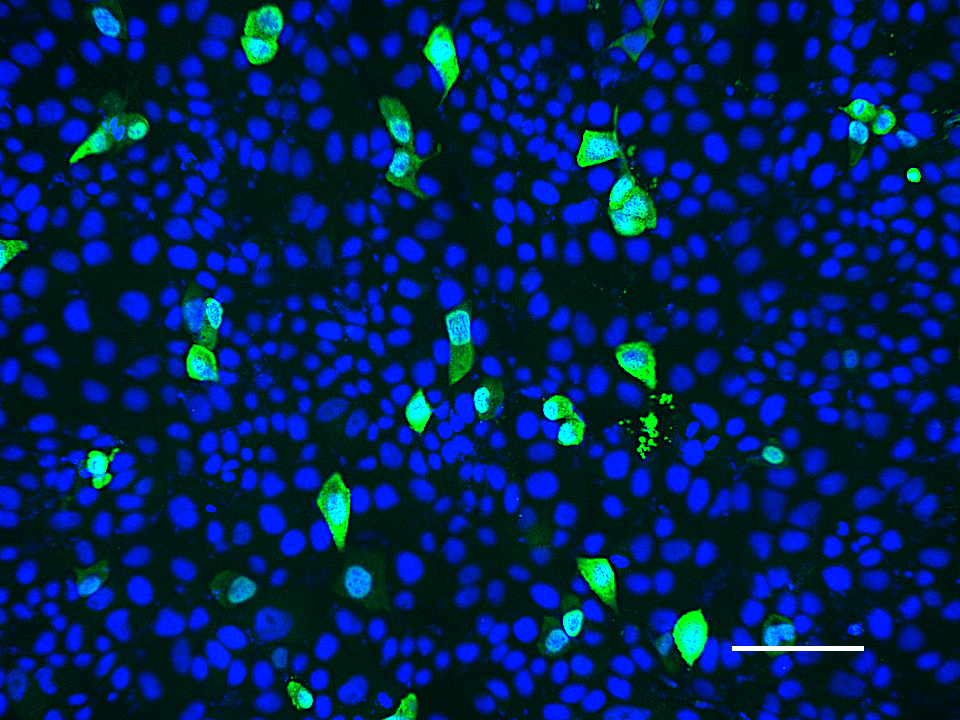

Supplement: S6 File — (ZIP) [file pone.0248960.s008.zip › Figure 4 WSN raw data tif files/S Baku 125â╩M 2_20_35.tif]

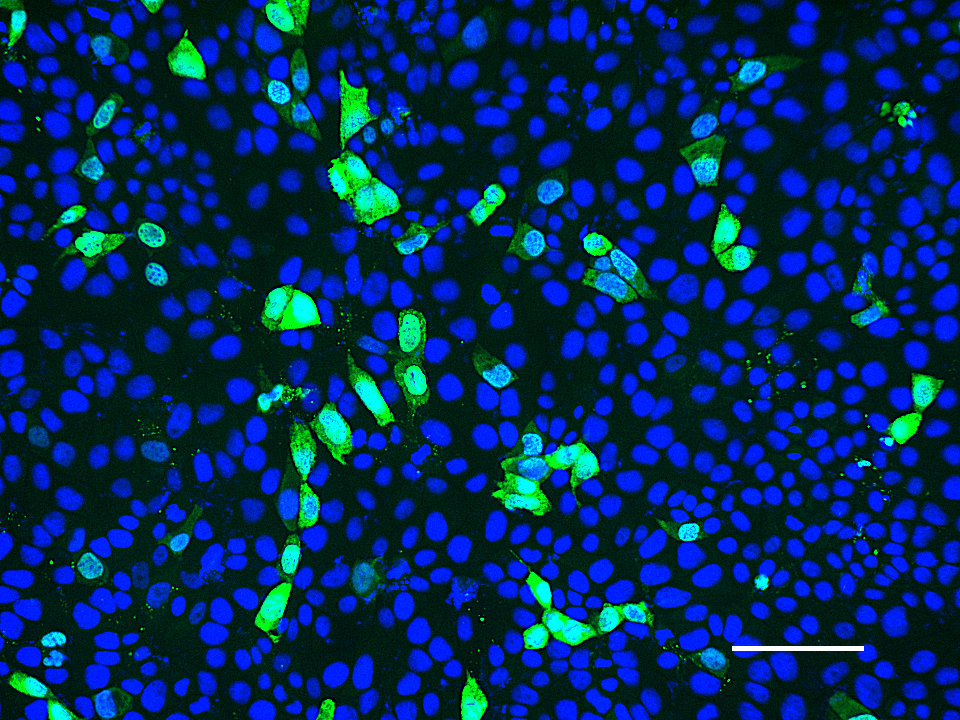

Supplement: S6 File — (ZIP) [file pone.0248960.s008.zip › Figure 4 WSN raw data tif files/S Baku 3125â╩M 3_20_47.tif]

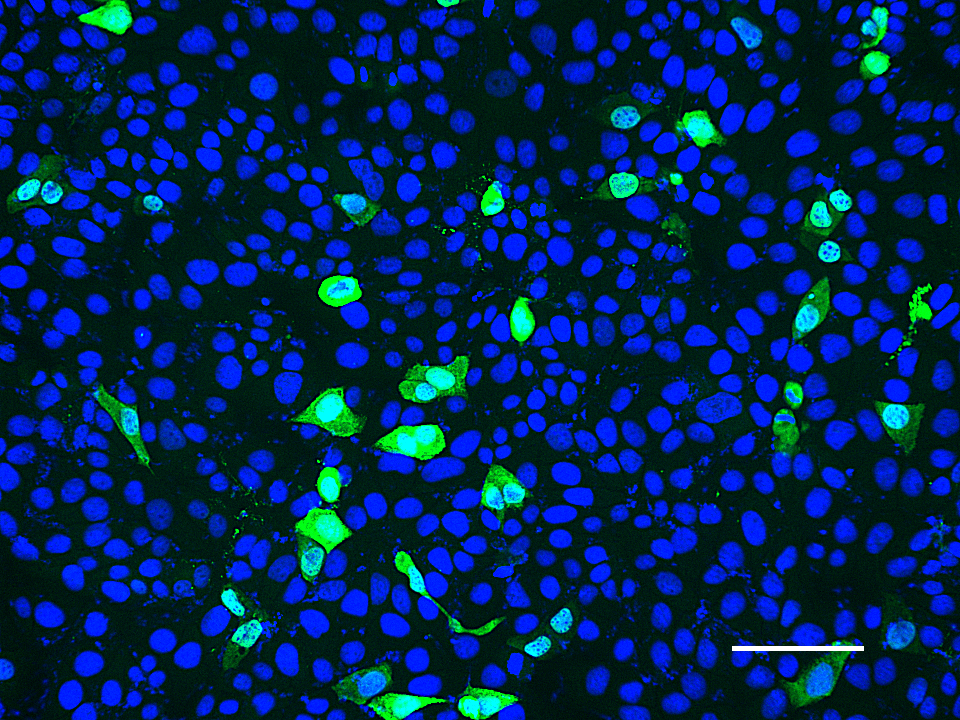

Supplement: S6 File — (ZIP) [file pone.0248960.s008.zip › Figure 4 WSN raw data tif files/S Baku 625â╩M 1_20_29.tif]

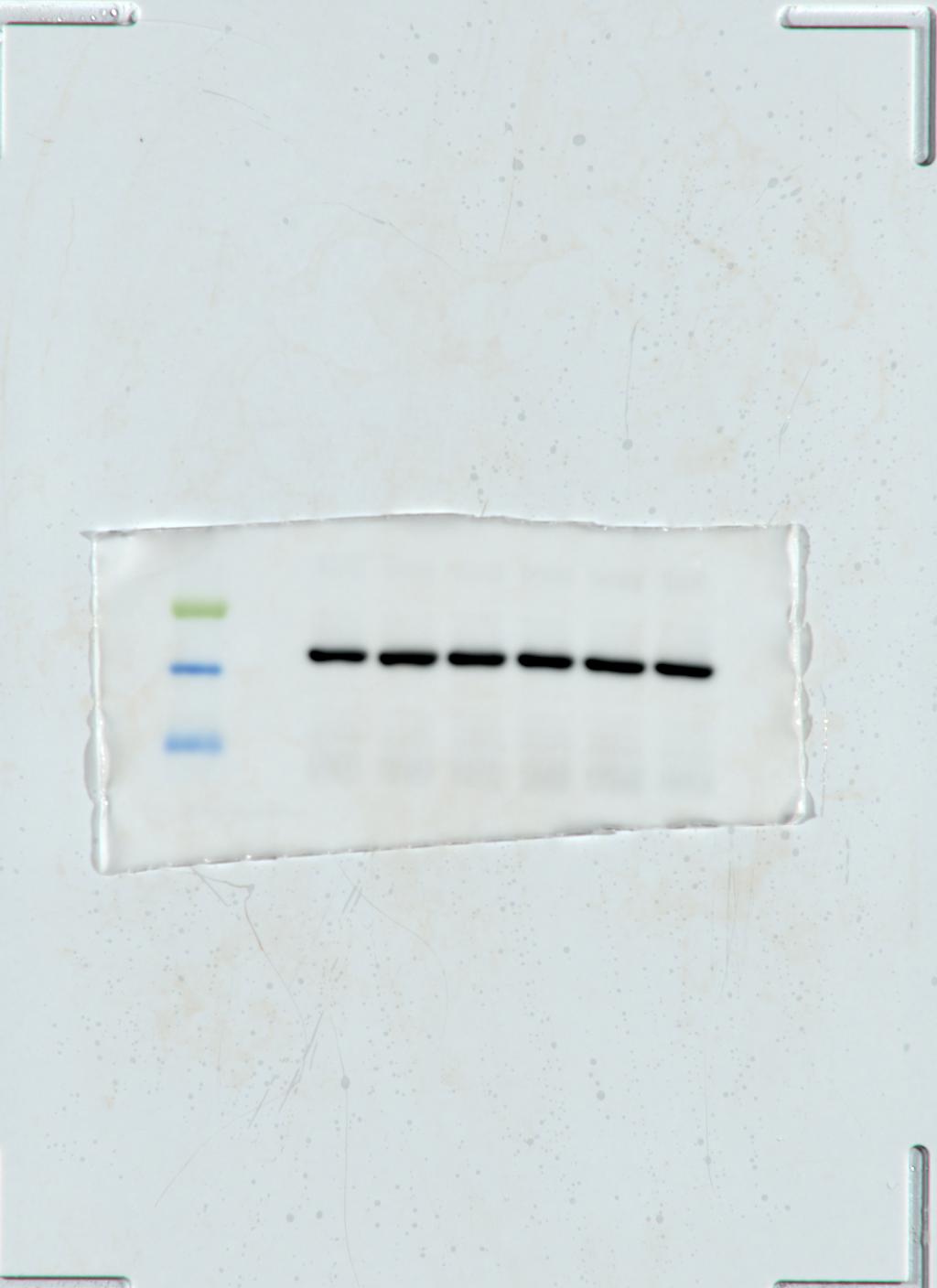

Supplement: S10 File — (ZIP) [file pone.0248960.s012.zip › Figure 7 cyclobakuchiol PR8 WB raw data/cyclobakuchiol 24h b semi.jpg]

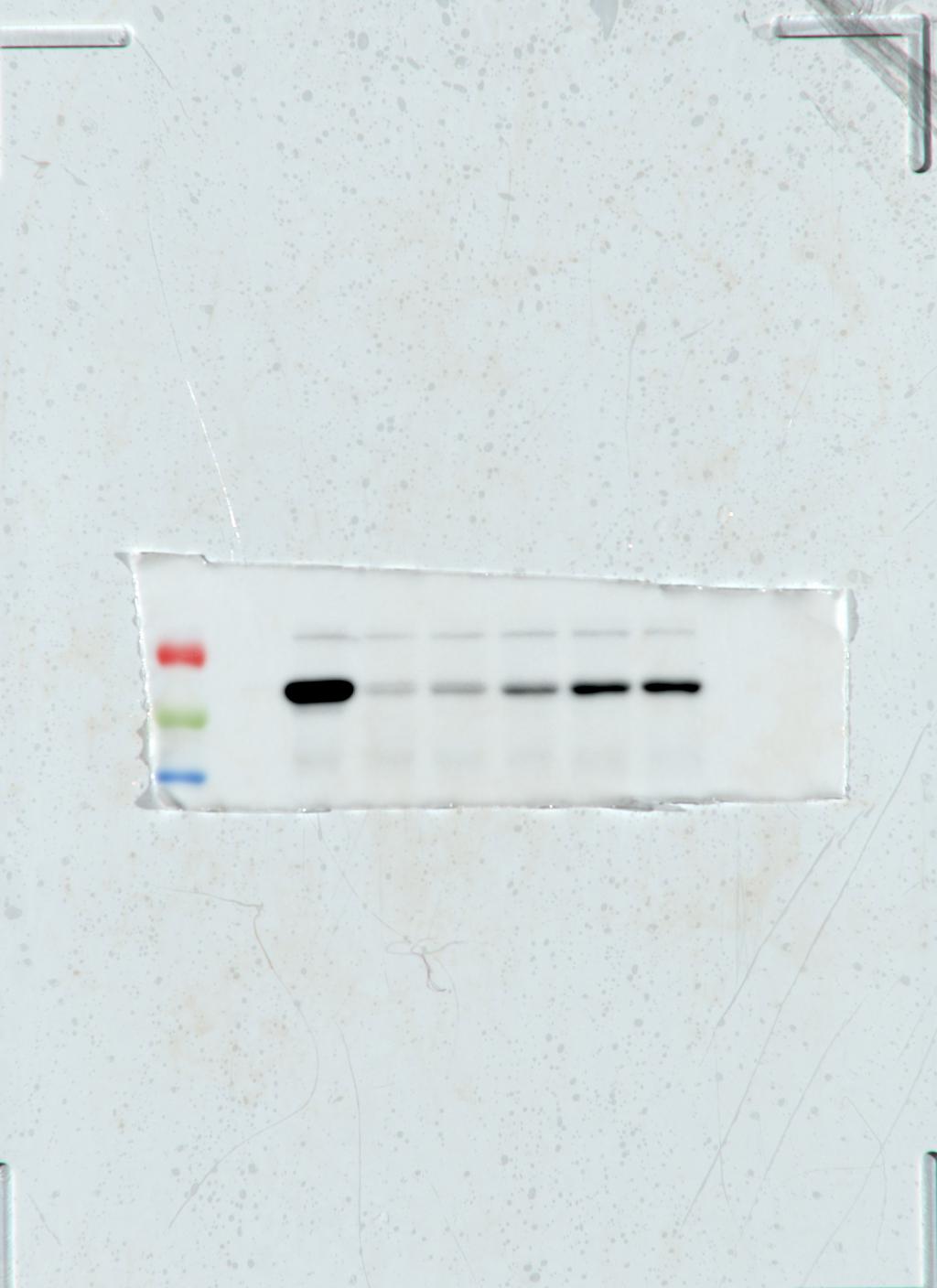

Supplement: S10 File — (ZIP) [file pone.0248960.s012.zip › Figure 7 cyclobakuchiol PR8 WB raw data/cyclobakuchiol 24h np semi.jpg]

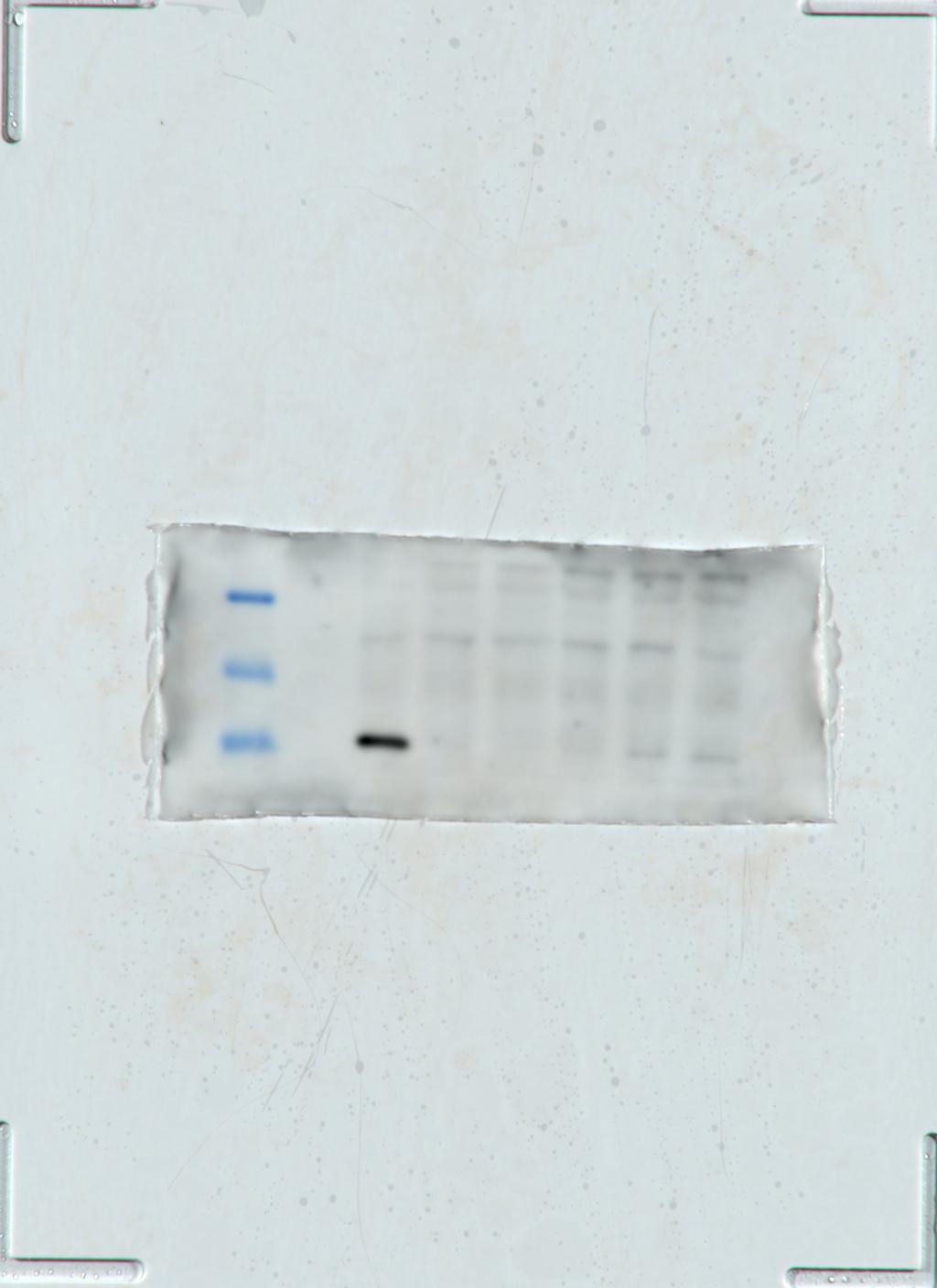

Supplement: S10 File — (ZIP) [file pone.0248960.s012.zip › Figure 7 cyclobakuchiol PR8 WB raw data/cyclobakuchiol 24h ns1.jpg]

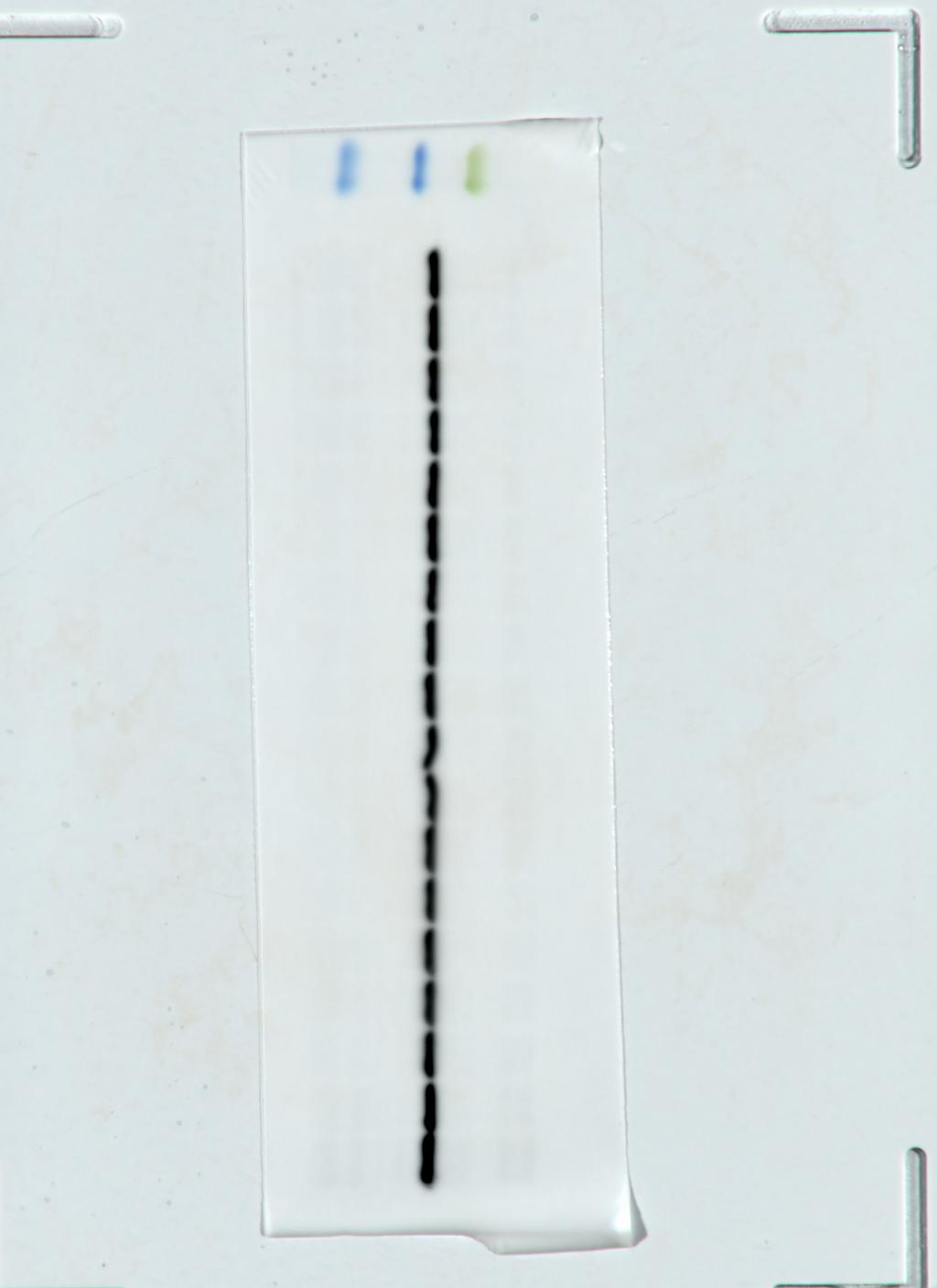

Supplement: S10 File — (ZIP) [file pone.0248960.s012.zip › Figure 7 cyclobakuchiol PR8 WB raw data/cyclobakuNo3 4-12h b semi.jpg]

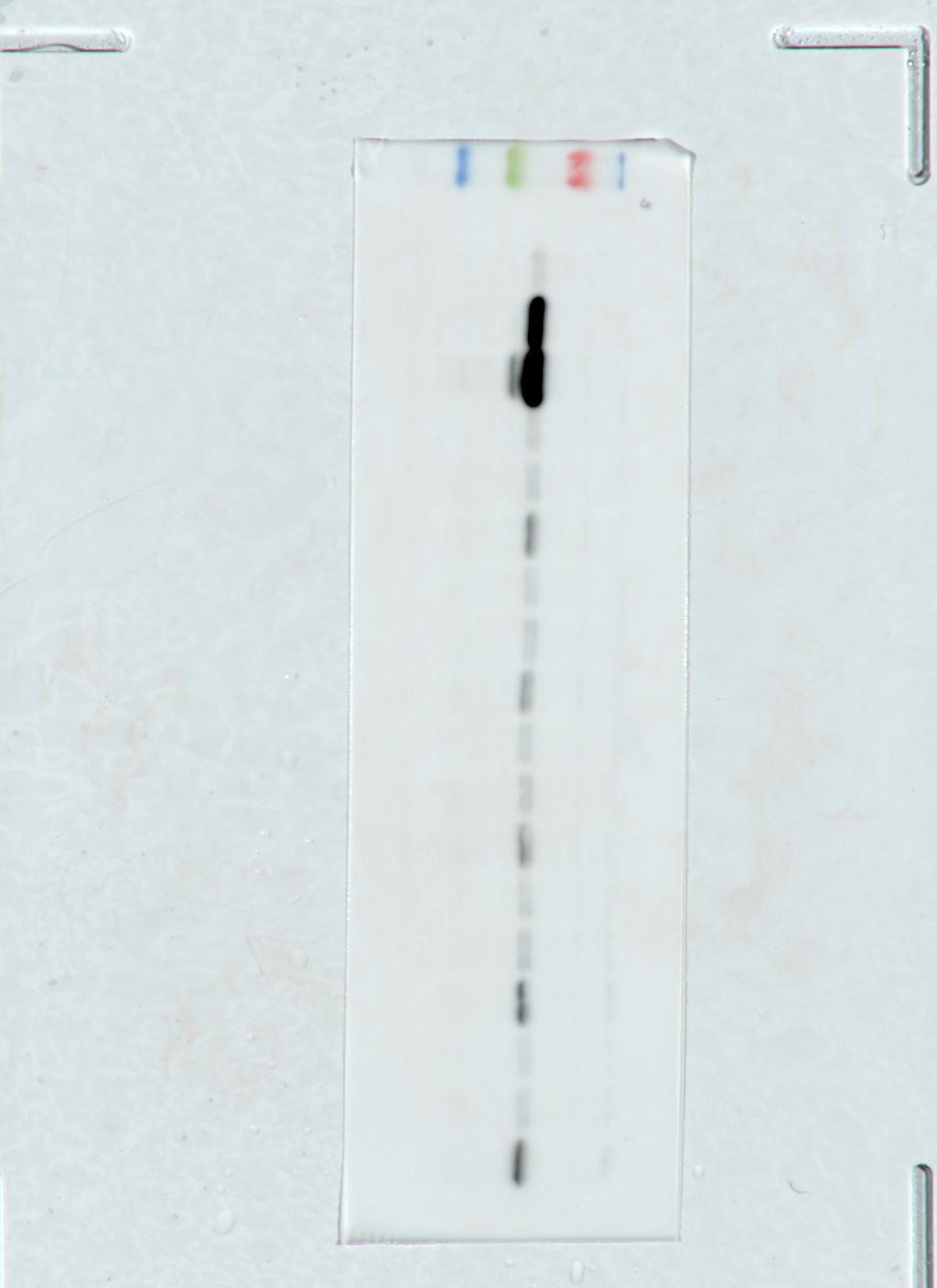

Supplement: S10 File — (ZIP) [file pone.0248960.s012.zip › Figure 7 cyclobakuchiol PR8 WB raw data/cyclobakuNo3 4-12h NP semi.jpg]

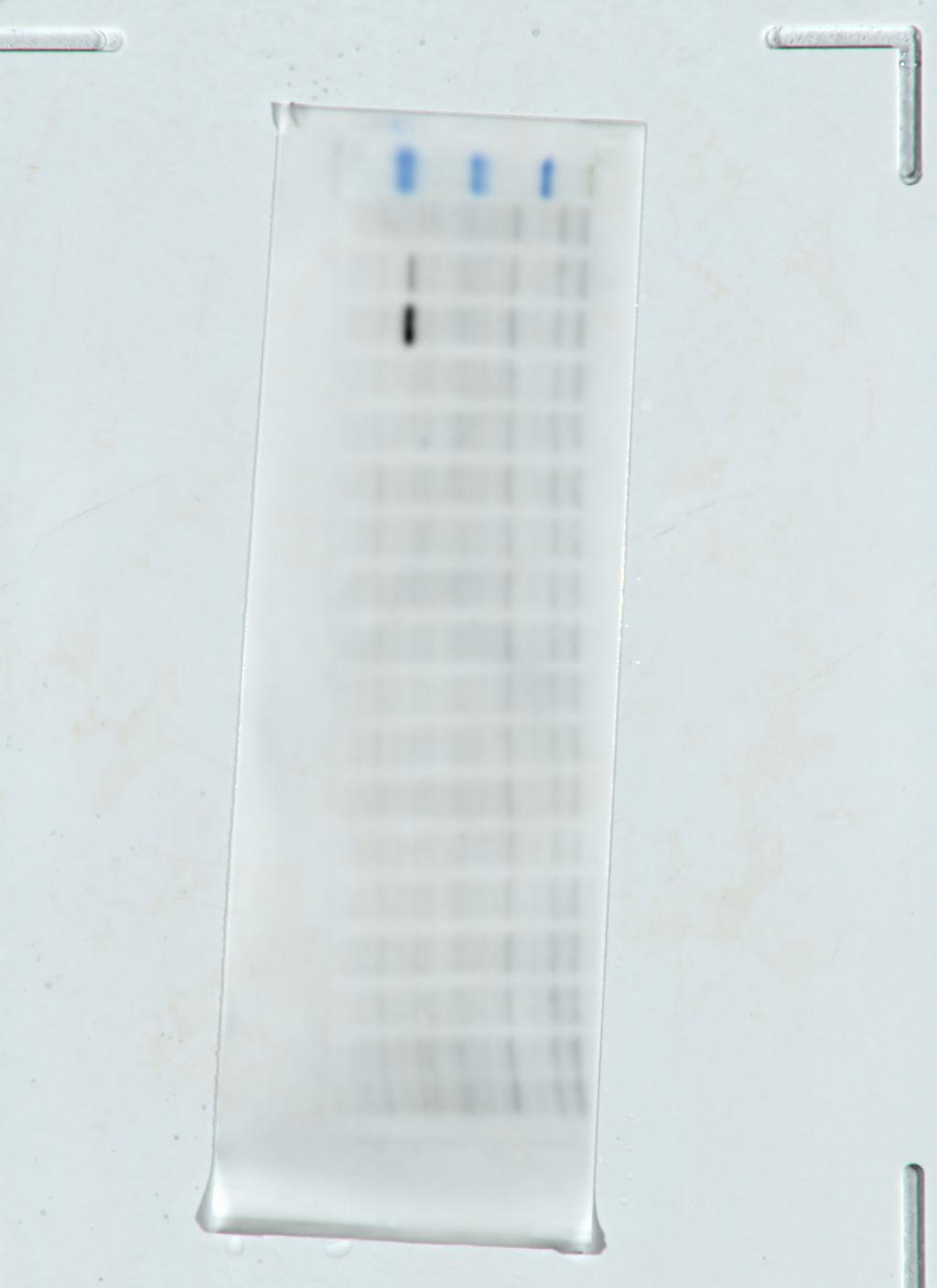

Supplement: S10 File — (ZIP) [file pone.0248960.s012.zip › Figure 7 cyclobakuchiol PR8 WB raw data/cyclobakuNo3 4-12h NS1 3m.jpg]
